# Updated Cardiovascular Prevention Guideline of the Brazilian Society of Cardiology - 2019

**DOI:** 10.5935/abc.20190204

**Published:** 2019-10

**Authors:** Dalton Bertolim Précoma, Gláucia Maria Moraes de Oliveira, Antonio Felipe Simão, Oscar Pereira Dutra, Otávio Rizzi Coelho, Maria Cristina de Oliveira Izar, Rui Manuel dos Santos Póvoa, Isabela de Carlos Back Giuliano, Aristóteles Comte de Alencar Filho, Carlos Alberto Machado, Carlos Scherr, Francisco Antonio Helfenstein Fonseca, Raul Dias dos Santos Filho, Tales de Carvalho, Álvaro Avezum Jr., Roberto Esporcatte, Bruno Ramos Nascimento, David de Pádua Brasil, Gabriel Porto Soares, Paolo Blanco Villela, Roberto Muniz Ferreira, Wolney de Andrade Martins, Andrei C. Sposito, Bruno Halpern, José Francisco Kerr Saraiva, Luiz Sergio Fernandes Carvalho, Marcos Antônio Tambascia, Otávio Rizzi Coelho-Filho, Adriana Bertolami, Harry Correa Filho, Hermes Toros Xavier, José Rocha Faria-Neto, Marcelo Chiara Bertolami, Viviane Zorzanelli Rocha Giraldez, Andrea Araújo Brandão, Audes Diógenes de Magalhães Feitosa, Celso Amodeo, Dilma do Socorro Moraes de Souza, Eduardo Costa Duarte Barbosa, Marcus Vinícius Bolívar Malachias, Weimar Kunz Sebba Barroso de Souza, Fernando Augusto Alves da Costa, Ivan Romero Rivera, Lucia Campos Pellanda, Maria Alayde Mendonça da Silva, Aloyzio Cechella Achutti, André Ribeiro Langowiski, Carla Janice Baister Lantieri, Jaqueline Ribeiro Scholz, Silvia Maria Cury Ismael, José Carlos Aidar Ayoub, Luiz César Nazário Scala, Mario Fritsch Neves, Paulo Cesar Brandão Veiga Jardim, Sandra Cristina Pereira Costa Fuchs, Thiago de Souza Veiga Jardim, Emilio Hideyuki Moriguchi, Jamil Cherem Schneider, Marcelo Heitor Vieira Assad, Sergio Emanuel Kaiser, Ana Maria Lottenberg, Carlos Daniel Magnoni, Marcio Hiroshi Miname, Roberta Soares Lara, Artur Haddad Herdy, Cláudio Gil Soares de Araújo, Mauricio Milani, Miguel Morita Fernandes da Silva, Ricardo Stein, Fernando Antonio Lucchese, Fernando Nobre, Hermilo Borba Griz, Lucélia Batista Neves Cunha Magalhães, Mario Henrique Elesbão de Borba, Mauro Ricardo Nunes Pontes, Ricardo Mourilhe-Rocha

**Affiliations:** 1 Pontifícia Universidade Católica do Paraná (PUC-PR), Curitiba, PR - Brazil; 2 Sociedade Hospitalar Angelina Caron, Campina Grande do Sul, PR - Brazil; 3 Universidade Federal do Rio de Janeiro (UFRJ), Rio de Janeiro, RJ - Brazil; 4 Instituto de Cardiologia de Santa Catarina, São José, SC - Brazil; 5 Instituto de Cardiologia do Rio Grande do Sul, Porto Alegre, RS - Brazil; 6 Universidade Estadual de Campinas (UNICAMP), Campina, SP - Brazil; 7 Universidade Federal de São Paulo (UNIFESP), São Paulo, SP - Brazil; 8 Universidade Federal de Santa Catarina (UFSC), Florianópolis, SC - Brazil; 9 Universidade Federal do Amazonas (UFAM), Manaus, AM - Brazil; 10 Ministério da Saúde, Brasília, DF - Brazil; 11 Instituto do Coração (InCor) do Hospital das Clínicas da Faculdade de Medicina da Universidade de São Paulo (USP), São Paulo, SP - Brazil; 12 Hospital Israelita Albert Einstein, São Paulo, SP - Brazil; 13 Clínica Cardiosport de Prevenção e Reabilitação, Florianópolis, SC - Brazil; 14 Departamento de Ergometria e Reabilitação Cardiovascular da Sociedade Brazileira de Cardiologia (DERC/SBC), Rio de Janeiro, RJ - Brazil; 15 Universidade do Estado de Santa Catarina (UDESC), Florianópolis, SC - Brazil; 16 Hospital Alemão Oswaldo Cruz, São Paulo, SP - Brazil; 17 Universidade do Estado do Rio de Janeiro (UERJ), Rio de Janeiro, RJ - Brazil; 18 Hospital Pró-Cardíaco, Rio de Janeiro, RJ - Brazil; 19 Hospital das Clínicas da Universidade Federal de Minas Gerais, Belo Horizonte, MG - Brazil; 20 Faculdade de Ciências Médicas de Minas Gerias (CMMG) da Fundação Educacional Lucas Machado (FELUMA), Belo Horizonte, MG - Brazil; 21 Hospital Universitário Ciências Médicas (HUCM), Belo Horizonte, MG - Brazil; 22 Universidade Federal de Lavas (UFLA), Lavras, MG - Brazil; 23 Universidade de Vassouras, Vassouras, RJ - Brazil; 24 Hospital Universitário Clementino Fraga Filho da Universidade Federal do Rio de Janeiro (UFRJ), Rio de Janeiro, RJ - Brazil; 25 Hospital Samaritano, Rio de Janeiro, RJ - Brazil; 26 Universidade Federal Fluminense (UFF), Niterói, RJ - Brazil; 27 Complexo Hospitalar de Niterói, Niterói, RJ - Brazil; 28 Universidade de São Paulo (USP), São Paulo, SP - Brazil; 29 Saraiva & Berlinger LTDA, São Paulo, SP - Brazil; 30 Instituto Dante Pazzanese de Cardiologia, São Paulo, SP - Brazil; 31 Pronto Cardio, Santos, SP - Brazil; 32 Real Hospital Português de Beneficência, Recife, PE - Brazil; 33 Universidade Federal do Pará (UFPA), Belém, PA - Brazil; 34 Liga Hipertensão de Porto Alegre, Porto Alegre, RS - Brazil; 35 Liga de Hipertensão Arterial da Faculdade de Medicina da Universidade Federal de Goiás (UFG), Goiânia, GO - Brazil; 36 FGM Clínica Paulista de Doenças Cardiovasculares, São Paulo, SP - Brazil; 37 Universidade Federal de Alagoas (UFAL), Maceió, AL - Brazil; 38 Universidade Federal de Ciências da Saúde de Porto Alegre (UFCSPA), Porto Alegre, RS - Brazil; 39 Fundação Universitária de cardiologia do RS (ICFUC), Porto Alegre, RS - Brazil; 40 Universidade Federal do Rio Grande do Sul (UFRGS), Porto Alegre, RS - Brazil; 41 Secretaria de Estado da Saúde do Paraná, Curitiba, PR - Brazil; 42 Instituto de Cardiologia Preventiva de São Caetano do Sul, São Caetano do Sul, SP - Brazil; 43 Hospital do Coração (HCor), São Paulo, SP - Brazil; 44 Faculdade de Medicina de São José do Rio Preto, São José do Rio Preto, SP - Brazil; 45 Instituto de Moléstias Cardiovasculares, São José do Rio Preto, SP - Brazil; 46 Universidade Federal de Mato Grosso (UFMT), Cuiabá, MT - Brazil; 47 Universidade Federal de Goiás (UFG), Goiânia, GO - Brazil; 48 SOS Cardio, Florianópolis, SC - Brazil; 49 Universidade do Sul de SC (Unisul), Florianópolis, SC - Brazil; 50 Instituto Nacional de Cardiologia do Rio de Janeiro, Rio de Janeiro, RJ - Brazil; 51 Laboratório de Lípides (LIM10), Hospital das Clínicas da Faculdade de Medicina da Universidade de São Paulo (HCFMUSP), São Paulo, São Paulo, SP – Brazil; 52 Instituto de Nutrição Roberta Lara, Itu, SP - Brazil; 53 Diadia Nutrição e Gastronomia, Itu, SP - Brazil; 54 CLINIMEX, Rio de Janeiro, RJ - Brazil; 55 Fitcordis Medicina do Exercício, Brasília, DF - Brazil; 56 Universidade Federal do Paraná (UFPR), Curitiba, PR - Brazil; 57 Santa Casa de Misericórdia de Porto Alegre, Porto Alegre, RS - Brazil; 58 Hospital Santa Joana Recife, Recife, PE - Brazil; 59 Hospital Agamenon Magalhães, Recife, PE - Brazil; 60 Universidade Federal da Bahia (UFBA), Salvador, BA - Brazil; 61 Cardio Clínica do Vale, Lajeado, RS - Brazil; 62 Hospital São Francisco, Porto Alegre, RS - Brazil

**Table:** In this update, grade of recommendations and level of evidence were applied in accordance with the following standards:

Grade of recommendation	
Grade I	Conditions for which there is conclusive evidence or, in the absence of conclusive evidence, general consensus that the procedure is safe and useful/effective
Grade IIa	Conditions for which there are conflicting evidence and/or divergent opinions regarding the procedure's safety and usefulness/effectiveness. Weight or evidence/opinion in favor of the procedure. The majority of studies/experts approve.
Grade IIb	Conditions for which there are conflicting evidence and/or divergent opinions regarding the procedure's safety and usefulness/effectiveness. Safety and usefulness/effectiveness less well established, with no prevailing opinions in favor.
Grade III	Conditions for which there is evidence and/or consensus that the procedure is not useful/effective and may, in some cases, be potentially harmful

**Table:** 

Level of evidence	
Level A	Data obtained from multiple concordant large randomized trials and/or robust meta-analysis of randomized clinical trials
Level B	Data obtained from less robust meta-analysis, from a single randomized trial, or from non-randomized (observational) trials
Level C	Data obtained through consensus of expert opinion

**Table:** 

Declaration of potential conflict of interest of authors/collaborators of the Updated Cardiovascular Prevention Guideline of the Brazilian Society of Cardiology - 2019 If the last three years the author/developer of the Statement:							
Names Members of the Updated	Participated in clinical studies and/or experimental trials supported by pharmaceutical or equipment related to the guideline in question	Has spoken at events or activities sponsored by industry related to the guideline in question	It was (is) advisory board member or director of a pharmaceutical or equipment	Committees participated in completion of research sponsored by industry	Personal or institutional aid received from industry	Produced scientific papers in journals sponsored by industry	It shares the industry
Adriana Bertolami	No	No	No	No	No	No	No
Aloyzio Cechella Achutti	No	No	No	No	No	No	No
Álvaro Avezum Júnior	No	No	No	No	No	No	No
Ana Maria Lottenberg	No	No	No	No	No	No	No
André Ribeiro Langowiski	No	No	No	No	Torrent, Boehringer	No	No
Andrei C. Sposito	Amgen, AstraZeneca	Amgen, Sanofi Aventis	Amgen, Sanofi Aventis	No	No	No	No
Andrea Araújo Brandão	Novartis	Abbott, Daiichi Sankyo, EMS, Libbs, Novartis, Medley, Merck, Servier	No	No	No	Abbott, Biolab, Chiesi, Daiichi Sankyo, Libbs, Medley, Novartis, Biolab, Boehringer, Servier	No
Antonio Felipe Simão	No	No	Daiichi Sankyo, Bayer, Schitech	No	No	No	No
Aristóteles Comte de Alencar Filho	No	No	No	No	No	No	No
Artur Haddad Herdy	No	No	No	No	No	No	No
Audes Diógenes de Magalhães Feitosa	No	No	No	No	No	No	No
Bruno Halpern	No	No	No	No	No	No	No
Bruno Ramos Nascimento	No	No	No	No	No	No	No
Carla Janice Baister Lantieri	No	No	No	No	No	No	No
Carlos Alberto Machado	No	No	No	No	No	No	No
Carlos Daniel Magnoni	No	Libbs	No	No	No	Libbs, Biolab, FQM	No
Carlos Scherr	No	No	No	No	No	No	No
Celso Amodeo	No	Novartis, Novonordisk, Pfizer, Biolab	No	Biolab, Servier	Novonordisk, Pfizer, Biolab, Daiichi Sankyo, Novartis	No	No
Claudio Gil Soares de Araujo	No	No	No	No	Inbramed	No	No
Dalton Bertolim Précoma	No	No	Servier, Bayer	Daiichi Sankyo	Servier, Bayer, Daiichi Sankyo	No	No
David de Pádua Brasil	Bayer	Libbs, Servier	No	Bayer	No	Libbs, Servier	No
Dilma do Socorro Moraes de Souza	No	No	No	No	No	No	No
Eduardo Costa Duarte Barbosa	No	EMS, Servier	No	No	Servier	EMS, Servier, Medley	No
Emilio Hideyuki Moriguchi	No	No	Daiichi Sankyo Brasil, Biolab	No	Biolab, Kowa	Baldacci, Novartis	No
Fernando Antonio Lucchese	No	No	No	No	No	No	No
Fernando Augusto Alves da Costa	No	No	No	No	No	No	No
Fernando Nobre	No	No	No	No	No	No	No
Francisco Antonio Helfenstein Fonseca	Pfizer, Amgen, Sanofi Aventis, Aché, Libbs, Novartis	Amgen, Sanofi Aventis, Aché, Biolab, EMS, Novartis, Abbott, Takeda, Novo Nordisk, Libbs, Sandoz	Amgen, Sanofi Aventis, Abbott, Biolab, Aché, Libbs, Novartis, Novo Nordisk, Takeda, Bayer	Novartis, Aegerion, Amgen	AstraZeneca	EMS, Biolab, Aché, Sandoz, Libbs	No
Gabriel Porto Soares	No	No	No	No	No	No	No
Glaucia Maria Moraes de Oliveira	No	No	No	No	No	No	No
Harry Correa Filho	No	No	No	No	No	No	No
Hermes Toros Xavier	No	Abbott, Aché, Aegerion, Amgen, Chiesi, MSD, Novartis, Sanofi Aventi, Torrent	Amgen, Torrent	No	No	Abbott, Aché, Amgen, Chiesi, Hypermarcas, Libbs, Merck, Supera, Torrent	No
Hermilo Borba Griz	No	No	No	No	No	No	No
Isabela de Carlos Back Giuliano	No	No	No	No	No	No	No
Ivan Romero Rivera	No	No	No	No	No	No	No
Jamil Cherem Schneider	No	No	No	No	No	No	No
Jaqueline Ribeiro Scholz	No	No	No	No	No	No	No
Jose Carlos Aidar Ayoub	No	No	No	No	No	No	No
José Francisco Kerr Saraiva	No	No	No	Pfizer, Novartis, Boehringer, Novonordisk, AstraZeneca	No	Pfizer, Novartis, Boehringer, Novonordisk, AstraZeneca	No
José Rocha Faria-Neto	No	Sanofi, AMGEM, Medley, MSD, Boehringer Ingelheim, AstraZeneca, Jansen, Pfizer, Novo Nordisk	Sanofi, MSD, Boehringer Ingelheim, AstraZeneca, Jansen, Novo Nordisk	No	No	No	No
Lucélia Batista Neves Cunha Magalhães	No	No	No	No	No	No	No
Lucia Campos Pellanda	No	No	No	No	No	No	No
Luiz Cézar Nazário Scala	No	No	No	No	No	No	No
Luiz Sérgio Fernandes de Carvalho	Astra Zeneca, Amgen	Roche, Amgen	No	No	No	Novo Nordisk, Libbs	No
Marcelo Chiara Bertolami	No	Abbott, Aché, Libbs, Merck, Marjan, Amgen, Sanofi Aventis	Sanofi Aventis	No	No	Abbott, Sanofi Aventis, Libbs, Aché	No
Marcelo Heitor Vieira Assad	No	No	No	No	No	No	No
Marcio Hiroshi Miname	Kowa, Amgen, Sanofi	Sanofi-Regeneron, Amgen	No	No	No	No	No
Marcos Antônio Tambasci	No	No	No	No	No	No	No
Marcus Vinícius Bolivar Malachias	No	Abbott, Biolab, Libbs, Novo Nordisk, Takeda	No	No	No	Abbott, Biolab,Libbs, Novo Nordisk	No
Maria Alayde Mendonçada Silva	No	No	No	No	No	No	No
Maria Cristina de Oliveira Izar	Amgen, Sanofi, Pfizer, Novartis, Akcea/Ionis	Amgen, Abbott, Aché, Libbs, Sanofi, EMS, NovoNordisk	No	No	AstraZeneca	Amgen, Sanofi, Libbs, Aché, Abbott, Farmoquímica, Eurofarma	No
Mario Fritsch Toros Neves	No	No	No	No	Servier	No	No
Mário Henrique Elesbão de Borba	No	No	No	No	No	No	No
Mauricio Milani	No	No	No	No	No	No	No
Mauro Ricardo Nunes Pontes	No	No	No	Boehringer, Takeda	AstraZeneca	No	No
Miguel Morita Fernandes da Silva	No	No	No	No	Novartis	No	No
Oscar Pereira Dutra	No	Sankyo, Sanofi Aventis, AstraZeneca, Amgen	Sankyo, Bayer	No	Sanofi Aventis, Bayer, AstraZeneca	Aché	No
Otávio Rizzi Coelho	No	Boehringer, AstraZeneca, Lilly, Takeda, Bayer, Novo Nordisk	Lilly, Sanofi Aventis	No	Boehringer, AstraZeneca, Lilly, Takeda, Bayer, Novo Nordisk	Libbs, Bayer	No
Otávio Rizzi Coelho-Filho	No	No	No	No	No	No	No
Paolo Blanco Villela	No	No	No	No	No	No	No
Paulo Cesar Brandão Veiga Jardim	No	No	No	No	Servier	Biolab, Servier, Libbs	No
Raul Dias dos Santos Filho	Amgen, Sanofi, Kowa, Pfizer	Amgen, Ache, AstraZeneca, Biolab, Novo Nordisk, MSD, Merck SA, Sanofi Aventis	Amgen, AstraZeneca, Akcea, Kowa, Novo Nordisk, Sanofi Aventis, Regeneron	Kowa, Pfizer	No	Biolab, Novo Nordisk	No
Ricardo Mourilhe-Rocha	No	No	No	No	No	No	No
Ricardo Stein	No	No	No	No	No	No	No
Roberta Soares Lara	No	No	No	No	No	No	No
Roberto Esporcatte	No	No	No	No	Bayer, Pfizer, Servier, Biosensors	No	No
Roberto Muniz Ferreira	No	No	No	No	No	No	No
Rui Manuel dos Santos Povoa	No	No	No	No	No	No	No
Sandra Cristina Pereira Costa Fuchs	No	No	No	No	No	No	No
Sergio Emanuel Kaiser	Sanofi Aventis	Amgen, Momenta Farma	No	No	No	Momenta Farma	No
Silvia Maria Cury Ismael	No	No	No	No	No	No	No
Tales de Carvalho	No	No	No	No	No	No	No
Thiago de Souza Veiga Jardim	No	AstraZeneca, Libbs, Torrent, Merck	No	No	Torrent, Bayer	Chiesi, Torrent	No
Viviane Zorzanelli Rocha Giraldez	No	No	No	No	No	No	No
Weimar Kunz Sebba Barroso de Souza	No	No	No	No	No	No	No
Wolney de Andrade Martins	No	No	No	No	Servier	Sanofi	No

## Introduction

Cardiovascular disease (CVD) is the leading cause of death worldwide and in Brazil, leading to increased morbidity and disability-adjusted life year (DALY). Despite the decrease in mortality rates and DALY standardized by age in Brazil, possibly as a result of successful health policies, their total number is increasing, mainly due to aging and illnesses in the population.^1^

Classical risk factors (hypertension, dyslipidemia, obesity, sedentary lifestyle, smoking, diabetes, and family history) raise the pre-test probability of CVD - particularly of coronary artery disease (CAD) - and determine primary and secondary prevention. Several other factors, including sociodemographic, ethnic, cultural, dietary, and behavioral aspects, can also explain the differences in CVD burden among populations and their trends over the decades. The implementation of health policies, among them, encouraging healthy lifestyle habits and providing access to primary and secondary CVD prevention measures, associated with the treatment of cardiovascular (CV) events are essential to control CVD in all countries, including Brazil.

The I Brazilian Cardiovascular Prevention Guideline of the Brazilian Society of Cardiology (*Sociedade Brasileira de Cardiologia* - SBC), published in 2013,^2^ aimed at helping reduce CV mortality, as established by the World Health Assembly in May 2012; SBC reaffirmed its commitment to decreasing the premature CVD mortality rate by 25%.^3^ However, the reduction in CVD mortality has reached a plateau in the past five years in Brazil, with significant regional variation, suggesting the need for renewing strategies to combat these diseases.^4^ With this purpose, SBC revisited its CV prevention guideline,^2^ proposing to update themes related to the primary prevention of CVD and suggesting strategies that could assist Brazilian cardiologists in reducing morbidity and mortality from these groups of causes.

The Brazilian Cardiovascular Prevention Guideline of the Brazilian Society of Cardiology - 2019 updates the strategies that address classical risk factors and discusses new concepts, such as the need to gather knowledge about emerging risk factors - for instance, spirituality -, socioeconomic and environmental factors, as well as additional strategies, like the use of vaccines.

We hope to contribute to renew the SBC commitment with the Brazilian society and the Strategic Action Plan for tackling Chronic Non-Communicable Diseases (NCD),^5^ of which CVD is the main component, with an instrument that will allow systematized access to the current literature, disseminating the knowledge necessary to resume the decreasing trend in CV mortality in Brazil.

## 1. Risk Stratification

### 1.1. Cardiovascular Risk Stratification to Prevent and Treat Atherosclerosis

The first manifestation of atherosclerotic disease in approximately half of the people who have this complication is an acute coronary event. Therefore, identifying asymptomatic individuals with higher predisposition is crucial for effective prevention associated with the correct definition of therapeutic targets. The so-called risk scores and algorithms based on regression analysis of population studies were created to estimate the severity of CVD, substantially enhancing the identification of overall risk. The Framingham gloal risk score (GRS)^6^ included the estimate of 10 years of coronary and cerebrovascular events, peripheral arterial disease, or heart failure (HF) and was the score adopted by the Department of Atherosclerosis of SBC (*Departamento de Aterosclerose da Sociedade Brasileira de Cardiologia* - SBC-DA).^7^

In addition, individuals who have multiple risk factors for CV, subclinical atherosclerosis, or already had manifestations of CVD have a high risk for events and can be classified differently.

Thus, the new CV risk stratification proposed by the SBC-DA defines four levels of CV risk:

- Very high risk
- High risk
- Moderate risk
- Low risk

Strategies for primary or secondary prevention of the disease are proposed based on the characterization of CV risk.

### 1.2. Very High Risk

Individuals who have a significant atherosclerotic disease (coronary, cerebrovascular, or peripheral vascular) with or without clinical events belong to this category (Chart 1.1).

**Chart 1.1:** Individuals with very high cardiovascular risk according to the Department of Atherosclerosis of the Brazilian Society of Cardiology^7^

Significant atherosclerosis (≥ 50% obstruction) with or without clinical events in the following territories
• Coronary
• Cerebrovascular
• Peripheral vascular

### 1.3. High Risk

Patients in primary prevention who present ORS > 20% (men) or > 10% (women) or aggravating risk conditions based on clinical data or subclinical atherosclerosis (Chart 1.2).

**Chart 1.2:** Individuals with high cardiovascular risk according to the Department of Atherosclerosis of the Brazilian Society of Cardiology^7^

• Men with overall risk score > 20%
• Women with overall risk score > 10%
• Subclinical atherosclerosis documented by:
- Carotid ultrasound with the presence of plaque
- ABI < 0.9
- CACS > 100 Agatston U
- Atherosclerotic plaques in coronary computed tomography angiography
• Abdominal aortic aneurysm
• CKD defined by Glomerular Filtration Rate < 60 mL/min in the non-dialysis stage
• Patients with LDL-c ≥ 190 mg/dL
• Type 1 or 2 diabetes, with LDL-c between 70 and 189 mg/dL, and presence of RS* or SAD**

ABI: Ankle-Brachial Index; CACS: Coronary Artery Calcium Score; CKD: chronic kidney disease; LDL-c: low-density lipoprotein-cholesterol; RS: Risk Stratifiers; SAD: Subclinical Atherosclerotic Disease.

* Age ≥ 48 years in men and ≥ 54 years in women; time to diabetes diagnosis > 10 years; family history of premature CVD (< 55 years for men and < 65 years for women) in first degree relative; smoking (at least one cigarette in the previous month); systemic arterial hypertension; metabolic syndrome, according to the International Diabetes Federation; albuminuria > 30 mg/g creatinine and/or retinopathy; glomerular filtration rate < 60 mL/min.

** Carotid ultrasound with presence of plaque > 1.5 mm; ABI < 0.9; coronary calcium score > 10 Agatston units; atherosclerotic plaques in coronary computed tomography angiography; LDL-c between 70 and 189 mg/dL, with overall risk score > 20% for males and > 10% for females.

### 1.4. Moderate Risk

The estimated risk for atherosclerotic disease results from the sum of the risk associated with each risk factor and the powering caused by synergisms between some of these factors. Given the complexity of these interactions, intuitive risk allocation often leads to under- or overestimation of higher or lower risk cases, respectively. Among the algorithms created to stratify CV risk, the last Updated Brazilian Guideline for Dyslipidemia and Atherosclerosis Prevention recommends the use of ORS, which estimates the risk for myocardial infarction, cerebrovascular accident (CVA), HF - fatal or non-fatal -, or peripheral vascular insufficiency in 10 years.

Based on this score, individuals with GRS ranging from 5 to 20% (males) and 5 to 10% (females) are classified as moderate risk. Patients with diabetes mellitus (DM) without SAD criteria or RS are also considered at moderate risk. Many middle-aged individuals belong to this risk category (Chart 1.3). Part of the latest recommendations leans towards inflammatory conditions and the use of coronary calcium to restratify patients at moderate risk.^8^

**Chart 1.3:** Moderate Risk according to the Department of Atherosclerosis of the Brazilian Society of Cardiology

• Male patients with GRS from 5 to 20%
• Female patients with GRS from 5 to 10%
• Diabetic patients without RS* or SAD** factors

GRS: overall risk score; RS: risk stratifiers; SAD: subclinical atherosclerotic disease.

* Age ≥ 48 years in men and ≥ 54 years in women; time to diabetes diagnosis > 10 years; family history of premature CVD (< 55 years for men and < 65 years for women) in first degree relative; smoking (at least one cigarette in the previous month); systemic arterial hypertension (SAH); metabolic syndrome, according to the International Diabetes Federation; albuminuria > 30 mg/g creatinine and/or retinopathy; glomerular filtration rate < 60 mL/min.

** Carotid ultrasound with presence of plaque > 1.5 mm; ABI < 0.9; coronary calcium score > 10 Agatston units; atherosclerotic plaques in coronary computed tomography angiography; LDL-c between 70 and 189 mg/dL, with ORS > 20% for males and > 10% for females.

### 1.5. Low Risk

Any estimated CV risk based on findings of observational studies inevitably has limitations related to calibration and discriminatory power: the attempt to allocate a certain risk percentage to each patient collides with individual aspects, not covered by risk prediction equations. The idea of restoring the concept of aggravating risk - understood as individual phenotypic expressions causally related to greater chances of a CV outcome - to improve somewhat the individualization of the algorithms created from large population samples has been gaining strength.^8^ However, in the low-risk population stratum, an aggravating risk in those with less than 5% chance of having a CV outcome in 10 years^6,8^ would hardly have a decisive influence in this relatively short time interval. On the other hand, as age is one of the most important determinants of risk for CV events, a man aged 62 years, without SAD, normotensive, non-smoker, non-diabetic, and with optimal levels of serum lipids would already be classified by ORS as moderate risk, even without any aggravating factor.^6^

Therefore, adults considered at low CV risk are those aged 30 to 74 years, of both genders, whose risk for CV events calculated by GRS is lower than 5% in 10 years^6,7^ (Chart 1.4).

**Chart 1.4:** Patients at low cardiovascular risk according to the Department of Atherosclerosis of the Brazilian Society of Cardiology^7^

• Men with an overall risk score < 5%
• Women with an overall risk score < 5%

Although the calcium score is not recommended for low-risk patients, non-diabetic individuals at moderate risk, without a family history of premature coronary disease, who have a zero calcium score can be considered at low risk and postpone the start of the cholesterol-lowering therapy with statins.^8^

Aggravating risk factors are not used in patients considered at low CV risk. The North American guideline of 2018 considers restratifying moderate risk to low in patients with zero calcium score (non-diabetics and without a family history of premature coronary disease).^8^

Charts 1.5, 1.6, 1.7, and 1.8 present the GRS for men and women in 10 years.

**Chart 1.5:** Score according to overall risk for women^2^

Score	Age (years)	HDL-c	TC	SBP (untreated)	SBP (treated)	Smoking	Diabetes	
-3				< 120				
-2		60+						
-1		50-59			< 120			
0	30-34	45-49	< 160	120-129		No	No	
1		35-44	160-199	130-139				
2	35-39	< 35		140-149	120-139			
3			200-239		130-139	Yes		
4	40-44		240-279	150-159			Yes	
5	45-49		280+	160+	140-149			
6					150-159			
7	50-54				160+			
8	55-59							
9	60-64							
10	65-69							
11	70-74							
12	75+							

HDL-c: high-density lipoprotein-cholesterol; SBP: systolic blood pressure; TC: total cholesterol.

**Chart 1.6:** Overall risk for women in 10 years^2^

Score	Risk (%)	Score	Risk (%)
≤ -2	< 1	13	10.0
-1	1.0	14	11.7
0	1.2	15	13.7
1	1.5	16	15.9
2	1.7	17	18.5
3	2.0	18	21.6
4	2.4	19	24.8
5	2.8	20	28.5
6	3.3	21+	> 30
7	3.9		
8	4.5		
9	5.3		
10	6.3		
11	7.3		
12	8.6		

**Chart 1.7:** Score according to overall risk for men^2^

Score	Age (years)	HDL-c	TC	SBP (untreated)	SBP (treated)	Smoking	Diabetes	
-2		60+		< 120				
-1		50-59						
0	30-34	45-49	< 160	120-129	< 120	No	No	
1		35-44	160-199	130-139				
2	35-39	< 35	200-239	140-159	120-139			
3			240-279	160+	130-139		Yes	
4			280+		140-159	Yes		
5	40-44				160+			
6	45-49							
7								
8	50-54							
9								
10	55-59							
11	60-64							
12	65-69							
13								
14	70-74							
15	75+							
Score								Total

HDL-c: high-density lipoprotein-cholesterol; SBP: systolic blood pressure; TC: total cholesterol.

**Chart 1.8:** Overall risk for men in 10 years^2^

Score	Risk (%)	Score	Risk (%)
≤ -3	< 1	13	15.6
-2	1.1	14	18.4
-1	1.4	15	21.6
0	1.6	16	25.3
1	1.9	17	29.4
2	2.3	18+	> 30
3	2.8		
4	3.3		
5	3.9		
6	4.7		
7	5.6		
8	6.7		
9	7.9		
10	9.4		
11	11.2		
12	13.2		

Table 1.1 summarizes the recommendations for cardiovascular risk stratification.

**Table 1.1:** Recommendations for cardiovascular risk stratification

Recommendation	Recommendation grade	Level of evidence	Reference
Routine evaluation of cardiovascular risk factors in adults aged 40 to 75 years, according to GRS for 10 years (Charts 1.5, 1.6, 1.7, 1.8; Figure 1.1)	I	B	^2,9,10^
Routine evaluation of cardiovascular risk factors in adults aged 20 to 39 years, according to GRS for each 4 to 6 years (Charts 1.5, 1.6, 1.7, 1.8; Figure 1.1)	IIa	B	^2,9,10^
For adults with borderline (5 to < 7.5%/10 years) or moderate (≥ 7.5 to < 20%/10 years) risk, including aggravating factors is recommended to guide therapeutic decisions	IIa	B	^2,9,10^
Adults with borderline (5 to < 7.5%/10 years) or moderate risk (≥ 7.5 to < 20%/10 years) can have their calcium score assessed to guide therapeutic decisions	IIa	B	^2,9,10^
The risk to life or for 30 years can be considered in adults aged 20 to 59 years with an estimated risk < 7.5%/10 years	IIb	B	^2,9,10^

GRS: global risk score.

**Figure 1.1:**
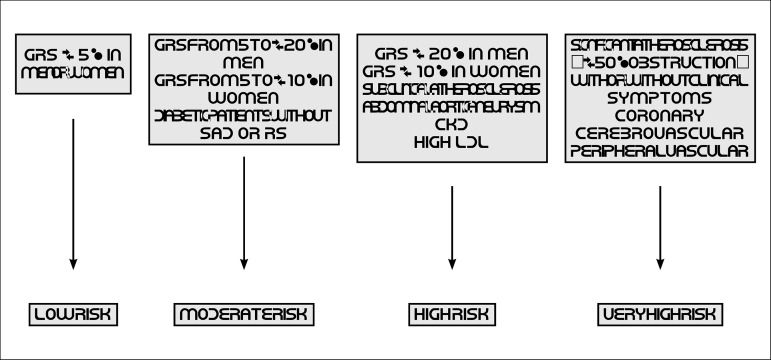
Cardiovascular risk stratification. CKD: chronic kidney disease (glomerular filtration rate < 60 ml/min/m^2^, non-dialysis); GRS: global risk score; RS: risk stratifiers; SAD: subclinical atherosclerotic disease.

## 2. Dyslipidemia

### 2.1. Introduction

Dyslipidemias represent an important CV risk factor, with low-density lipoprotein cholesterol (LDL-c) as the most relevant modifiable risk factor for CAD.^11^ Genetic^12^ and clinical studies with statins and other lipid-lowering drugs provide ample evidence that lower LDL-c levels are associated with the proportional decrease in CV outcomes, including myocardial infarction, CVA, and CV death.^13,14^

The 2017 Updated Brazilian Guideline for Dyslipidemia incorporated some changes in the approach of dyslipidemias compared to the previous version.^7^ One of the changes was that fasting was no longer mandatory for total cholesterol (TC) and high-density lipoprotein cholesterol (HDL-c) tests, provided that the laboratory specifies the situation in the report, without fasting or with 12-hour fasting. As for triglycerides (TG), it might increase in the absence of fasting. In hypertriglyceridemia, particularly with a value > 440 mg/dL, a new collection after 12-hour fasting is crucial.^15^ Apolipoprotein (ApoA1 and ApoB) levels can be determined in a sample without prior fasting, and moderately high TG levels do not influence immunochemical methods. The analytical performance of this methodology is good, and the levels can be measured in automated platforms with an immunoturbidimetry or nephelometry profile.

There is evidence of an independent association between elevated lipoprotein (a) [Lp(a)] and CVD risk in the general population,^16^ not only for the lipid content of Lp(a) but also for its prothrombotic and proinflammatory properties. The gold standard for quantification of plasma concentrations is the measurement of Apo(a) mass by turbidimetry, nephelometry, or chemiluminescence, using isoform-insensitive assays, which are little affected by the heterogeneity in Apo(a) isoforms. It does not require fasting and provides accurate data. Its analysis is not recommended for routine assessment of CVD risk in the general population, but it should be determined in the risk stratification of individuals with a family history of premature atherosclerotic disease and familial hypercholesterolemia (FH).^7^ Lp(a) values above 50 mg/dL, equivalent to 80%, are considered high; if the result is in nmol/L, it should be multiplied by 2.5, with Lp(a) values above 125 nmol/L classified as high.^7^

Table 2.1 reports the reference values of the lipid profile with and without fasting, according to the evaluation of CV risk in adults.

**Table 2.1:** Reference values, according to the evaluation of cardiovascular risk estimated for adults over 20 years of age

Lipids	With fasting (mg/dL)	Without fasting (mg/dL)	Risk category
Total cholesterol	< 190	< 190	Desired
HDL-c	> 40	> 40	Desired
Triglycerides	< 150	< 175	Desired
LDL-c*	< 130	< 130	Low
	< 100	< 100	Moderate
	< 70	< 70	High
	< 50	< 50	Very high
Non-HDL-c	< 160	< 160	Low
	< 130	< 130	Moderate
	< 100	< 100	High
	< 80	< 80	Very high

HDL-c: high-density lipoprotein-cholesterol; LDL-c: low-density lipoprotein-cholesterol.

* LDL-c values calculated by the Martin formula.^7,51^ Adapted from the Updated Guideline for Dyslipidemia and Atherosclerosis Prevention.^7^

The primary (LDL-c) and secondary (non-high-density lipoprotein cholesterol - non-HDL-c) therapeutic targets for lipid control are established following the risk stratification of patients (discussed in Chapter 1). This stratification considers the presence or absence of clinical or subclinical atherosclerotic disease, the presence of diabetes, and the GRS, with subsequent risk classification into four possible categories: low (< 5%), moderate (5-10% in women and 5-20% in men), high (> 10% in women and > 20% in men), and very high (clinical atherosclerotic cardiovascular disease, > 30%) risk. Chapter 1 presents the complete risk stratification. Specific targets for each category were defined in accordance with Table 2.1.^7^

The Updated Brazilian Guideline for Dyslipidemia and Atherosclerosis Prevention^7^ also included a change in CV risk stratification for individuals already using statins. Considering the imprecision of risk calculation in these patients, the guideline proposes using a correction factor for TC to estimate the risk score in this context, derived from studies that compared the efficacy of different statins in the doses used and that allowed an average LDL-c reduction of ~ 30% with the treatment.^17^ This situation applies to most patients who take moderate doses of statins. Given the average TC reduction of 30% with statins, patients who use these medicines should have their TC multiplied by 1.43.^17^ Moreover, in the initial approach, the target for individuals who are not on lipid-lowering treatment should be decreasing the percentage of LDL-c and non-HDL-c. For those already on lipid-lowering therapy, the recent guideline also established a reduction in absolute LDL-c and non-HDL-c values with the treatment, as shown in Table 2.2.

**Table 2.2:** LDL-c and non-HDL-c percentage reduction and absolute therapeutic targets in patients who use and do not use lipid-lowering drugs

Risk	Without lipid-lowering drugs	With lipid-lowering drugs	
	Reduction (%)	LDL-c target (mg/dL)	Non-HDL-c target (mg/dL)
Very high	> 50	< 50	< 80
High	> 50	< 70	< 100
Moderate	30-50	< 100	< 130
Low	> 30	< 130	< 160

LDL-c: low-density lipoprotein-cholesterol; non-HDL-c: non-high-density lipoprotein-cholesterol. Adapted from the Updated Brazilian Guideline for Dyslipidemia and Atherosclerosis Prevention.^7^

#### 2.1.1. Familial Hypercholesterolemia

FH is a genetic condition characterized by very high LDL-c levels and, therefore, increased risk for premature atherosclerotic disease, especially of a coronary event. However, despite its importance, this condition is still underdiagnosed and undertreated.^18,19^ This version of the guideline reinforces that greatly increased cholesterol values could indicate FH, after excluding secondary dyslipidemias. Adult individuals with TC values ≥ 310 mg/dL or children and adolescents with levels ≥ 230 mg/dL should be evaluated for this possibility. Among the clinical scores available for FH, we highlight the Dutch Lipid Clinic Network score, used in our field, and presented in Table 2.3. In addition to clinical scores, the genetic test for FH is a very useful, but not mandatory, tool to confirm suspected cases and screen relatives of established index cases.

**Table 2.3:** Diagnostic criteria for familial hypercholesterolemia (based on the Dutch Lipid Clinic Network criteria - Dutch MEDPED)

Parameter	Score
Family history First degree relative with premature vascular/coronary disease (men < 55 years, women < 60 years) OR First or second degree relative with TC > 290 mg/dL* First degree relative with tendon xanthoma and/or corneal arcus OR First degree relative < 16 years with TC > 260 mg/dL*	1 2
Clinical history Patient with premature CAD (men < 55 years, women < 60 years) Patient with premature cerebral or peripheral arterial disease (men < 55 years, women < 60 years)	2 1
Physical examination Tendon xanthoma Corneal arcus < 45 years	6 4
LDL-c Levels (mg/dL) ≥ 330 mg/dL 250 - 329 mg/dL 190 - 249 mg/dL 155 - 189 mg/dL	8 5 3 1
DNA analysis Functional mutation in the LDL receptor, the ApoB100, or the PCSK9* gene	8
FH diagnosis Confirmed if Potential if Possible if Not FH	> 8 points 6 - 8 points 3 - 5 points < 3 points

CAD: coronary artery disease; DNA: deoxyribonucleic acid; FH: familial hypercholesterolemia; LDL-c: low-density lipoprotein-cholesterol; TC: total cholesterol.

* Modified from the Dutch MEDPED, adopting a criterion from the Simon Broome Register Group proposal. Adapted from the Updated Guideline for Dyslipidemia and Atherosclerosis Prevention (5) and the I Brazilian Guidelines for Familial Hypercholesterolemia.^19^

### 2.2. Dyslipidemia Treatment

#### 2.2.1. Non-Pharmacological Therapy

Nutritional therapy, weight loss, and the practice of physical activity should be recommended for all patients. Table 2.4 describes the dietary recommendations for the treatment.

**Table 2.4:** Dietary recommendations for the treatment of dyslipidemia

Recommendations	LDL-c			Triglycerides	
	Within the target and without comorbidities* (%)	Above the target or with comorbidities* (%)	Borderline 150-199 mg/dL (%)	High 200-499 mg/dL (%)	Very high† > 500 mg/dL (%)
Weight loss	Maintaining a healthy weight	5-10	Up to 5	5-10	5-10
Carbohydrate (%TEV)	50-60	45-60	50-60	50-55	45-50
Added sugars (%TEV)	< 10	< 10	< 10	5-10	< 5
Protein (%TEV)	15	15	15	15-20	20
Fat (%TEV)	25-35	25-35	25-35	30-35	30-35
Trans fatty acids (%TEV)	Exclude from diet				
Saturated fatty acids (%TEV)	< 10	< 7	< 7	< 5	< 5
Monounsaturated fatty acids (%TEV)	15	15	10-20	10-20	10-20
Polyunsaturated fatty acids (%TEV)	5-10	5-10	10-20	10-20	10-20
Linolenic acid, g/day	1.1-1.6				
EPA and DHA, g	-	-	0.5-1.0	> 2.0	> 2.0
Fiber	25 g, with 6 g of soluble fiber				

DHA: docosahexaenoic acid; EPA: eicosapentaenoic acid; TEV: total energy value. The reassessment period after implementing lifestyle modification measures should be 3 to 6 months. Adapted from the Updated Brazilian Guideline for Dyslipidemia and Atherosclerosis Prevention.^7^

#### 2.2.2. Drug Treatment Focused on Hypercholesterolemia

Statins are the first treatment choice for hypercholesterolemia, due to the evidence showing that their use decreases all-cause mortality, coronary ischemic events, need for revascularization, and CVA. LDL-c reduction varies among statins, a difference closely related to the initial dose, as shown in Table 2.5.

**Table 2.5:** Intensity of the lipid-lowering treatment

	Low	Moderate	High
Expected LDL-c reduction with daily dose, %	< 30	30-50	≥ 50
Examples, daily doses in mg	Lovastatin 20 Simvastatin 10 Pravastatin 10-20 Fluvastatin 20-40 Pitavastatin 1	Lovastatin 40 Simvastatin 20-40 Pravastatin 40-80 Fluvastatin 80 Pitavastatin 2-4 Atorvastatin 10-20 Rosuvastatin 5-10	Atorvastatin 40-80 Rosuvastatin 20-40 Simvastatin 40/ Ezetimibe 10

Note: the use of Ezetimibe alone reduces LDL-c in 18-20%. LDL-c: low-density lipoprotein-cholesterol. Adapted from the Updated Brazilian Guideline for Dyslipidemia and Atherosclerosis Prevention.^7^

Chart 2.1 presents the recommendations for lipid management and the evidence that supports such recommendations.

**Chart 2.1:** Recommendations for blood lipid management, recommendation grade, and level of evidence

Recommendation	Recommendation grade	Level of evidence	Reference
Individuals at very high CV risk: LDL-c should be reduced to < 50 mg/dL and non-HDL-c to < 80 mg/dL	I	B	^7^
Individuals at high CV risk: LDL-c should be reduced to < 70 mg/dL and non-HDL-c to < 100 mg/dL	I	A	^7^
Individuals at high and very high CV risk: whenever possible and tolerated, give preference to high-intensity statins or Ezetimibe associated with statin (Simvastatin 40 mg or another statin at least as potent)	I	A	^7^
Individuals at moderate CV risk: LDL-c should be reduced to < 100 mg/dL and non-HDL-c to < 130 mg/dL	I	A	^7^
Individuals at moderate CV risk: whenever possible and tolerated, give preference to statins of at least moderate intensity	I	A	^7^
Individuals at low CV risk: the LDL-c target should be < 130 mg/dL and non-HDL-c < 160 mg/dL	I	A	^7^
Drug therapy to increase HDL-c levels is not recommended	III	A	^7^
Individuals with TG levels > 500 mg/dL should receive appropriate therapy to reduce the risk for pancreatitis	I	A	^7^
Individuals with TG levels between 150 and 499 mg/dL should receive therapy based on CV risk and associated conditions	IIa	B	^7^

CV: cardiovascular; HDL-c: high-density lipoprotein cholesterol; LDL-c: low-density lipoprotein cholesterol; TG: triglycerides. The reassessment period after the drug treatment must be of at least a month. Adapted from the Updated Brazilian Guideline for Dyslipidemia and Atherosclerosis Prevention.^7^

Side effects are rare in statin treatment, but among them, muscular effects are the most common and can occur weeks or years after the start of treatment. They range from myalgia, with or without elevation of creatine kinase (CK), to rhabdomyolysis. CK levels should be evaluated at the start of treatment or when the dose needs to be increased, in case of muscle symptoms (pain, tenderness, stiffness, cramps, weakness, and localized or generalized fatigue), and when introducing drugs that might interact with statin (Recommendation Grade: IIa, Level of Evidence: B). The baseline evaluation of liver enzymes alanine aminotransferase (ALT) and aspartate aminotransferase (AST) must be performed before the beginning of statin therapy. During the treatment, the liver function should be assessed in case of signs or symptoms suggesting hepatotoxicity (fatigue or weakness, loss of appetite, abdominal pain, dark urine, or jaundice) (Recommendation Grade: IIa, Level of Evidence: B).^7^ Repeated analyses of enzyme samples in asymptomatic patients lead to additional costs with no benefit to patients.

Table 2.6 describes the indications for the association of other lipid-lowering drugs.

**Table 2.6:** Indications for the association of other lipid-lowering drugs (non-statins)

Recommendation	Recommendation grade	Level of evidence	Reference
**Ezetimibe**			
When the statin treatment in the maximum tolerated dose does not reach the LDL-c target in very high-risk patients	I	B	^7^
When the statin treatment in the maximum tolerated dose does not reach the LDL-c target in patients in primary prevention	IIb	C	^7^
Alone or in combination with statins represents a therapeutic option for patients who do not tolerate the recommended doses of statins	IIa	C	^7^
Can be used in case of fatty liver disease	IIb	C	^7^
**Resins**			
Adding cholestyramine to the statin treatment can be recommended when the LDL-c target is not reached despite the use of potent statins in effective doses	IIa	C	^7^
**PCSK9 Inhibitors**			
Indicated for patients at high CV risk, on optimized statin treatment at the highest tolerated dose, associated or not with Ezetimibe, and who have not reached the recommended LDL-c or non-HDL-c targets*	IIa	A	^7^

CV: cardiovascular; HDL-c: high-density lipoprotein cholesterol; LDL-c: low-density lipoprotein cholesterol. In very high-risk patients and some high-risk situations, when the individuals already take statin at the highest tolerated dose and Ezetimibe, the addition of a PCSK9 inhibitor is reasonable, despite the lack of an established long-term safety (> 3 years) for this drug and its low cost-effectiveness according to current data.^20^ Adapted from the Updated Brazilian Guideline for Dyslipidemia and Atherosclerosis Prevention.^7^

#### 2.2.3. Drug Treatment Focused on Hypertriglyceridemia

Hypertriglyceridemia is an independent risk factor for CVD, particularly for CAD.^21^ However, it is not clear if hypertriglyceridemia causes atherosclerosis, since TG does not tend to accumulate in arterial walls, or if the abnormalities associated with it, such as low HDL-c,^22-24^ small and dense LDL particles,^25,26^ insulin resistance,^27,28^ and increased blood coagulability and hyperviscosity,^29-31^ predispose the individual to atherosclerosis. According to Table 2.7, drug treatment for hypertriglyceridemia should be considered after the exclusion of secondary causes for the increase in TG - diabetes, renal failure, excessive alcohol intake, and use of certain medicines - and adjustments for behavioral measures.

**Table 2.7:** Indication of medicines for the treatment of hypertriglyceridemia

Recommendation	Recommendation grade	Level of evidence	Reference
**Fibrates**			
TG levels above 500 mg/dL	I	A	^32,33^
Mixed dyslipidemia with a prevalence of hypertriglyceridemia	IIa	B	^32,33^
In patients with diabetes, TG > 200 mg/dL, and HDL-c < 35 mg/dL, the combination of fenofibrate and statin might be considered when changing the lifestyle have failed	IIa	B	^32,33^
**Nicotinic acid (niacin)**			
There is no evidence that the drug benefits patients with controlled LDL-c	III	A	^32,33^
Exceptionally, it can be administered to patients with isolated low HDL-c and as an alternative to fibrates and statins, or in combination with these drugs in patients with hypercholesterolemia, hypertriglyceridemia, or resistant mixed dyslipidemia	IIa	A	^32,33^
**Omega-3 fatty acids**			
Patients with severe hypertriglyceridemia who did not reach the desired levels with the treatment can take high doses (4 to 10 g/day) of omega-3 fatty acids in combination with other lipid-lowering drugs	I	A	^32,33^
Supplementation with an E-EPA (ethyl eicosapentaenoic acid) formulation (4 g/day) can be recommended for high-risk patients with elevated TG levels using statins, as it seems to reduce the risk for ischemic events, including CV death*	I	B	^32,33^

CV: cardiovascular; EPA: eicosapentaenoic acid; HDL-c: high-density lipoprotein cholesterol; LDL-c: low-density lipoprotein cholesterol; TG: triglycerides.

* This formulation is not commercially available in our country. Adapted from I Brazilian Guidelines on Fat Consumption and Cardiovascular Health.^32^

Table 2.8 presents the recommended doses of fibrates available in our country and their effects on lipid profile

**Table 2.8:** Fibrate doses and lipid abnormalities (mean percentages)*

Drugs	Dose (mg/day)	TG reduction (%)	HDL-c increase (%)	LDL-c reduction (%)
Bezafibrate	200-600	30-60	7-11	Varying
Bezafibrate retard	400	30-60	7-11	Varying
Gemfibrozil	600-1200	30-60	7-11	Varying
Gemfibrozil retard	500	30-60	7-11	Varying
Etofibrate	500	30-60	7-11	Varying
Fenofibrate	160-250	30-60	7-11	Varying
Ciprofibrate	100	30-60	7-11	Varying

* Effects depend on the dose used and the initial baseline TG value. HDL-c: high-density lipoprotein-cholesterol; LDL-c: low-density lipoprotein-cholesterol; TG: triglycerides. Adapted from the Updated Brazilian Guideline for Dyslipidemia and Atherosclerosis Prevention.^7^

## 3. Diabetes and Metabolic Syndrome

### 3.1. Myocardial Risk

Patients with DM2 have a 2 to 5 times greater risk for HF compared to non-diabetic individuals.^34^ As CAD patients are excluded, the incidence of HF in the diabetic population decreases but remains significantly higher than in non-diabetic individuals. In type 1 diabetes, above 7%, each 1% increment in glycated hemoglobin (HbA1c) was associated with a 30% increase in HF risk,^35^ while type 2 diabetes was associated with a 16% increase in the risk, regardless of other risk factors, including obesity, smoking, hypertension, dyslipidemia, and coronary disease.^36,37^

Diabetic cardiomyopathy is characterized by myocardial fibrosis and left ventricular hypertrophy with diastolic dysfunction, initially asymptomatic, and that progresses slowly to diastolic or systolic dysfunction, followed by HF with clinical repercussion.^38^

Occasionally, diabetic cardiomyopathy can manifest as arrhythmias and sudden death. Mechanisms involved in the pathophysiological process include mitochondrial dysfunction, oxidative stress, inflammation, dysfunction in the mitochondrial Ca^2+^ management, activation of the renin-angiotensin-aldosterone system (RAAS) and the sympathetic nervous system (SNS), cardiac autonomic neuropathy, endoplasmic reticulum stress, microvascular dysfunction, and disorders of cardiac energy metabolism.^39-41^

#### 3.1.1. Myocardial Risk Estimate

Despite the lack of an universally accepted method to estimate HF risk specifically in diabetic individuals, methods such as plasma brain natriuretic peptide (BNP), echocardiographic evaluation of diastolic dysfunction, and risk calculators such as the Health ABC Heart Failure Score and the Framingham Heart Failure Risk Score are often used to estimate the future risk for symptomatic HF.

Elaborating a standardized strategy to screen and intervene in patients at HF risk might be difficult due to its different definitions, the heterogeneity of its prevalence in various populations, its inconstant duration until the development of clinic HF or left ventricular dysfunction, and the varying interventions to modify or treat risk factors. As we shall see below, the Health ABC Heart Failure Score is the mechanism with the highest sensitivity and specificity and should be recommended as the primary strategy in risk stratification of symptomatic HF. Nonetheless, BNP can be used concomitantly to reclassify individuals at high risk for HF.

The evidence that supports the use of BNP in diabetic patients to predict the HF risk is based on two randomized controlled trials. As shown in Table 3.1, these programs recruited 1,674 patients without HF for randomization and identified a total of 29 subsequent events of hospitalization for HF. The combined statistical power of these studies is limited but provides the perspective for the potential benefit of screening based on biomarkers such as BNP.

**Table 3.1:** BNP screening to guide the primary prevention strategy for diabetes mellitus

	Study design and intervention	Study population	N without prior HF	Hospitalizations for HF/follow-up duration	Effect on hospitalization for HF	Effect on major CV events*
STOP-HF^42^	Randomized controlled trial with BNP screening versus usual primary treatment	Age > 40 years without HF but with CV disease or CV risk factors	1,374	21 / 4.2 years	OR 0.48 (95% confidence interval 0.20-1.20)	OR 0.60 (95% confidence interval 0.45-0.81)
PONTIAC^43^	Randomized controlled study, with treatment in a cardiology outpatient clinic for titration of RAAS inhibitors and beta-blockers associated with care in a DM treatment unit versus care in an isolated DM unit	DM2 without known CV disease and NT-proBNP > 125 pg/mL	300	8 / 2 years	HR 0.14 (95% confidence interval 0.02-1.14)	HR 0.35 (95% confidence interval 0.13-0.97)

BNP: brain natriuretic peptide; CV: cardiovascular; DM: diabetes mellitus; HF: heart failure; RAAS: renin-angiotensin-aldosterone system.

* Major CV events, defined as unplanned hospitalizations for CV causes and deaths.

***Diastolic dysfunction on the echocardiogram -*** Historically, experts disagree on recommendations for echocardiographic diagnosis of diastolic dysfunction, as shown in the 2009 guidelines of the American Society of Echocardiography and the European Association of Cardiovascular Imaging (ASE/EACVI) and the Canberra Study Criteria (CSC).^44,45^ Based on these recommendations, epidemiological studies and a meta-analysis^46,47^ suggest that preclinical diastolic dysfunction (Stage B HF), defined as diastolic dysfunction with normal systolic function and without HF symptoms, is common in DM, and that its presence increases by 61 to 70% the risk for developing symptomatic HF (stages C and D). Despite being simple and non-invasive,^46,47^ the echocardiographic diagnosis for patients at higher risk for HF does not seem to be as cost-effective as the measurement of BNP,^48,49^ although these data are not specifically available for the Brazilian population.

The diagnostic criteria became more specific and less sensitive in the 2016 ASE/EACVI guideline,^50,51^ despite the simplification. With these criteria, the prevalence of diastolic dysfunction in the general population ranges from 1 to 7%. However, no studies have been designed to focus on primary prevention based on this diagnostic criterion.

***Risk scores for future HF -*** The HF risk in patients with DM and metabolic syndrome (MS) can be predicted with clinical scores. Although no scores have been developed specifically for patients with DM or MS, several studies have demonstrated good performance in these populations. Among the most used scores are the

- Health ABC Heart Failure Score;^52^ the
- The Framingham Heart Failure Risk Score;^53^ and the
- And the Atherosclerosis Risk in Communities (ARIC) Heart Failure Risk Score.^54^

The variables included in the Framingham Heart Failure Risk Score are age, gender, CAD, diabetes, left ventricular hypertrophy based on electrocardiogram (ECG), valvular disease, heart rate, and systolic blood pressure (SBP). The Health ABC Heart Failure Score includes the Framingham variables with the following differences: addition of serum albumin, serum creatinine, and smoking; replacement of glucose for diabetes; and exclusion of valvular disease. The ARIC Heart Failure Risk Score includes age, ethnicity, gender, CAD, diabetes, SBP, use of medicines for blood pressure (BP), heart rate, smoking, and body mass index (BMI).

**Figure 3.1:**
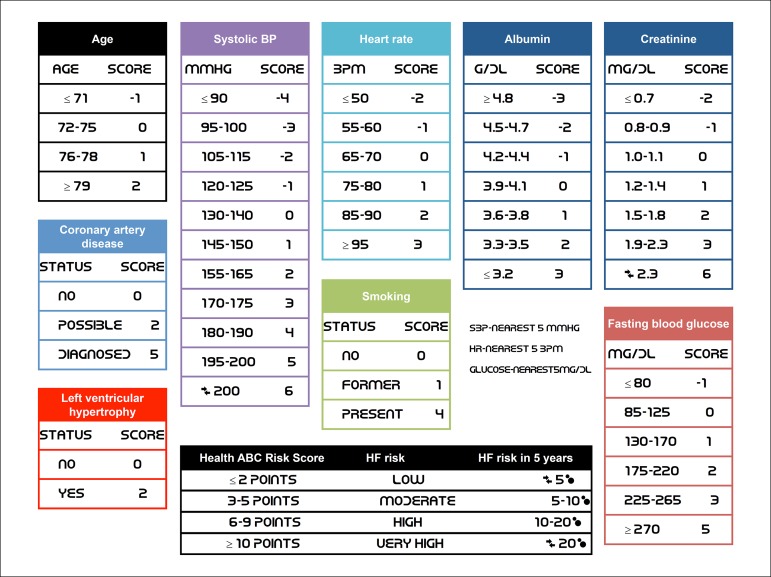
Health ABC Heart Failure Score.

Designed for a community population of older adults, the Health ABC Heart Failure Score reached a positive and negative predictive power of 10 and 15% in comparison with the Framingham Heart Failure Risk Score^52^ and 2 to 4% above the ARIC Heart Failure Risk.^54^ The Health ABC Heart Failure Score is an instrument validated in observational and intervention studies and, thus, considered a reference for estimating the future HF risk in patients with DM and MS (detailed description in Figure 3.1).

Although all scores are designed with only the variables listed above, the addition of BNP or NT-proBNP as linear variables would significantly increase the predictive power of all scores.^52,54^ Based on the thresholds used in the studies PONTIAC^43^ and STOP-HF,^42^ we suggest reclassifying individuals with BNP ≥ 50 pg/mL or NT-proBNP ≥ 125 pg/mL into a higher risk category.

#### 3.1.2. Preventive Therapies for Individuals at High and Very High Risk for Heart Failure in 5 Years and Secondary Prevention for Those with Clinical Heart Failure

***Drug Therapies for DM2 that impact HF -*** As stated previously, above 7%, the HF risk increases by 8% for each 1% increment in HbA1c, while a 1% reduction decreases the risk by 16%. Although, several clinical trials have investigated the effect of metformin on the CV system based on the pathophysiology of insulin resistance, the effect of this class directly on HF remains inconclusive. Studies with insulin and sulfonylureas showed a neutral effect on HF, and glucagon-like peptide-1 (GLP-1) agonists/analogs^55^ and acarbose^56^ proved to be neutral regarding the risk for HF hospitalizations and mortality.

More recently, three large studies - EMPA-REG, CANVAS, and DECLARE - revealed that sodium-glucose 2 (SGLT2) cotransporter inhibitors reduced CV outcomes, including HF hospitalizations.^57,58^ HF mortality among individuals who used empagliflozin was significantly lower than in those using a placebo. The studies EMPA-REG and DECLARE associated the risk of taking these drugs with a higher rate of genital infections in the group using empagliflozin and dapagliflozin, while the CANVAS study showed an increased risk of lower limb amputation.^57,58^ Together, all three SGLT2 inhibitors available (empagliflozin, canagliflozin, and dapagliflozin) reduce the risk for HF hospitalization, even in asymptomatic patients at the start of treatment. Therefore, the use of these drugs is recommended for patients with DM or MS at high or very high risk for HF.

Among the hypoglycemic agents that increase the chance of HF, we highlight the thiazolidinediones (RECORD study - rosiglitazone; and PROactive - pioglitazone)^59,60^ and a dipeptidyl peptidase-4 inhibitor (DPP-4i) - the saxagliptin (SAVOR-TIMI 53).^61^ In the studies RECORD and SAVOR-TIMI, patients with HF also had higher subsequent mortality rates. Thus, rosiglitazone, pioglitazone, and saxagliptin are contraindicated for patients with or at high risk for HF.

#### 3.1.3. Therapies Focused on Cardiac Remodeling

Although only two clinical trials substantiate these recommendations, patients with DM and MS at high and very high risk for HF seem to benefit from the early introduction of anti-remodeling therapies, such as RAAS inhibitors and beta-blockers. Based on these pharmacological strategies triggered by BNP or NT-proBNP levels above the risk threshold, the studies PONTIAC^43^ and STOP-HF^42^ suggested reducing the risk for HF hospitalization and mortality.

In patients with clinical HF, clinical trials have demonstrated that the drug therapies tested were equally effective, regardless of the presence of DM and MS.

***Angiotensin blockers -*** The CHARM Trial (candesartan),^62^ Val-HeFT (valsartan),^63^ and ATLAS (lisinopril)^64^ have demonstrated that the use of angiotensin-converting enzyme inhibitors (ACEI) or aldosterone-receptor blockers (ARB) favored the decrease in mortality and hospitalization among patients who had HF and reduced ejection fraction, regardless of the presence of DM2 or MS.

***Mineralocorticoid antagonists -*** Patients with and without DM2 showed a reduction in mortality, with the use of both spironolactone (RALES trial)^65^ and eplerenone (EMPHASIS-HF).^66^ We underline the risk for hyperkalemia, which might particularly affect patients with renal function deterioration and already using ACEI or ARB.

***Beta-blockers -*** In patients with DM and HF, the use of metoprolol succinate (MERIT-HF), bisoprolol (CIBIS II), and carvedilol (COPERNICUS) is recommended. They presented equal efficiency in patients with and without DM. A meta-analysis that included six trials indicated a reduction in all-cause mortality among patients with DM2, as well as in non-diabetic individuals.^67^

***Nitrates and Hydralazine -*** Approximately 40% of the patients randomized in the A-HeFT trial had DM2. In this subpopulation, the combination of a fixed dose of hydralazine and nitrate significantly reduced all-cause mortality.^68^

***Ivabradine -*** Its use decreased mortality and hospitalizations in patients with and without DM2 in the SHIFT study, which involved 6,558 patients.^69^

The sacubitril-valsartan combination is not well established yet in patients with preserved ejection fraction or at high risk for HF; even for patients with reduced ejection fraction, there is no specific study or subanalysis focused on the diabetic population.

### 3.2. Atherosclerotic Risk

#### 3.2.1. Metabolic Syndrome, Diabetes Mellitus, and the Continuous Corollary of Coronary Artery Disease

MS and the DM comprise a spectrum of multisystemic diseases, particularly in the vascular endothelium, that contribute dramatically to the progression of pathophysiological substrates of CAD. Robust evidence suggests that CV risk increases even in stages that precede the clinical diagnosis of DM in 10 to 20 years, based on current criteria. As MS is one of the main risk factors for DM, considering it within a *continuum* of metabolic changes related to coronary atherothrombosis is reasonable.^70,71^

**Figure 3.2:**
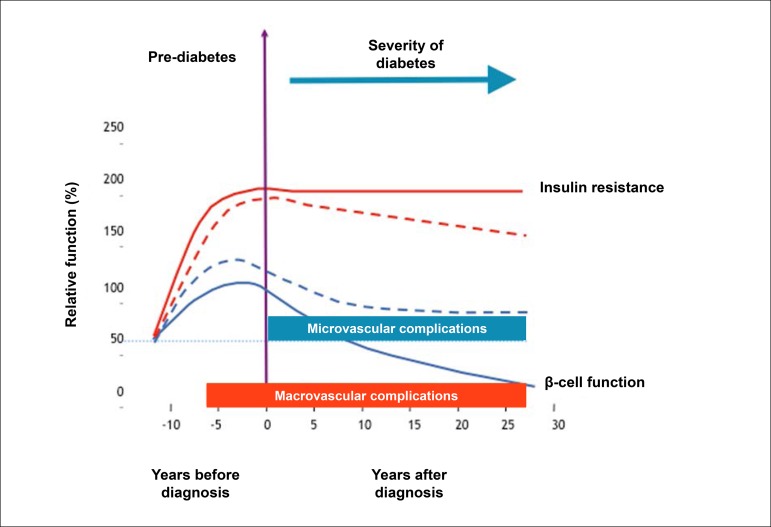
Progression of micro- and macrovascular disease in type 2 diabetes and its relationship with the functional reserve of pancreatic beta-cells and hyperglycemia.

In fact, estimates indicate that glucose metabolic changes precede the diagnosis of diabetes in 4 to 12 years^72^ (Figure 3.2). While in early stages the overproduction of insulin can compensate its resistance, after a certain point, the pancreatic functional reserve is exhausted, and the production of insulin no longer compensates its resistance. After this moment, the diagnosis will be established by hyperglycemia, but CV changes adaptive to insulin resistance and cellular oxidative stress become irreversible.

Another mechanism that seems to occur even in early stages (pre-hyperglycemia) is the accumulation of fatty acids in various tissues, such as pancreas, heart, and liver, accelerating the dysfunction in insulin production, hepatic glucose production, and left ventricular diastole.^73^ Therefore, even remotely before the period of hyperglycemia, several cellular mechanisms cooperate to determine the endothelial dysfunction, the phenotypic changes in lipids with hypertriglyceridemia, and small, dense LDL, creating the ideal scenario for accelerated atherogenesis.^74^ Together, these data suggest that CAD becomes accelerated many years before the onset of hyperglycemia.

#### 3.2.2. Primary Prevention Strategies for Coronary Artery Disease in Individuals with Metabolic Syndrome and Diabetes Mellitus

Corroborating the pathophysiological evidence, weight control strategies with physical activity and intensive dietary guidance have proven to be the best available method to reduce the risk of a patient with MS and pre-DM developing the clinical diagnosis of DM.^75,76^ Lifestyle interventions decreased the risk for DM by 45% (p = 0.001), the risk for CV death by 41% (p = 0.033), and all-cause mortality by 29% (p = 0.049).

In patients with established DM and microalbuminuria, the randomized clinical trial STENO-2^77^ showed that a multifactorial approach to lifestyle significantly decreased CV morbidity and mortality in comparison with conventional treatment.

#### 3.2.3. Individual Risk Prediction for Coronary Artery Disease in Patients with Diabetes Mellitus and Metabolic Syndrome

At least 68% of diabetic patients aged 65 years or older die from heart diseases, most of them from CAD, followed by congestive HF.^78^ DM is considered an independent CV risk factor both in men and women, raising in about two to four times the probability of clinical CAD, when compared to individuals without DM.^79^ Moreover, based on a meta-analysis with almost 1 million individuals from 87 studies, MS is associated with a twofold increase in CV outcomes and a 1.5 increase in all-cause mortality, exceeding the isolated risk of its components.^80^

The CAD risk in the population with DM or MS, however, is not evenly distributed. Several strategies for CAD screening were implemented in recent decades, although most of them have proven to be fruitless, as these groups are at high risk for CAD. Revascularization strategies guided by myocardial perfusion scintigraphy or coronary computed tomography angiography in asymptomatic diabetic individuals were not superior to clinical management, based only on traditional risk factors.

In the study FACTOR-64 - a randomized clinical trial with 900 patients with DM1 or DM2 for at least three years and without CAD symptoms -, the revascularization strategy guided by coronary computed tomography angiography did not reduce the risk for acute coronary syndrome (ACS) or CV mortality.^81^ Similarly, in the studies DIAD^82^ and DYNAMIT,^83^ the revascularization strategy guided by exercise stress test with scintigraphy did not improve CV and non-CV outcomes compared to conventional medical treatment in 1,900 asymptomatic diabetic patients.

Currently, the more efficient and practical resources to determine CV risk in diabetic patients have been the isolated control of its risk factors. Subanalyses of the Diabetes Heart Study^84^ and FACTOR-64^81^ revealed that the factors with greater predictive power for ACS risk were the use of statins and LDL-c levels, followed by glomerular filtration rate, microalbuminuria, and C-reactive protein (CRP).

The treatment of CV risk factors related to aggressive diabetes is the method more strongly associated with the reduction in CV morbidity and ACS mortality in diabetic patients, as demonstrated in the study STENO-2.^77^ However, as detailed below, the most effective way of predicting risk and managing more or less intensive targets in primary prevention should be combining risk and coronary calcium scores.

#### 3.2.4. Risk Calculator

Risk scores are among the most commonly used strategies, consisting of estimating risk based on prospective data collected from cohorts of diabetic patients, such as the UKPDS, the DECODE, the DARTS, the ADVANCE, the Swedish National Diabetes Register, and the DCS.^85,86^ Other calculators developed for mixed populations (diabetics and non-diabetics) are also widely used: ORS/SBC, Framingham, Pooled Cohort Equations (ASCVD), REYNOLDS, SCORE, PROCAM, and others.^74^ The main advantage of these methods lies in their easy application in clinical practice, as they consider the usual clinical data, such as age, laboratory test values, and anthropometric information. The UKPDS calculator is more recommended for diabetic patients (IDF21 guidelines, NICE, Canadian Diabetes Association, Australian National Vascular Disease Prevention Alliance, and others) and the ORS is the more widely used in the Brazilian diabetic and non-diabetic population.

Nevertheless, these and other strategies to estimate the progression of vascular diseases are still limited, underestimating the risk in young patients with DM or recently diagnosed patients, while overestimating the risk in individuals diagnosed for > 10 years or with HbA1c > 9.0%.^87-89^ Also, the scores do not take into account the advances of the last 5 to 10 years, such as new drugs and diagnostic methods, and have relatively low predictive performance (C-statistic between 0.54-0.70), considering that 30 to 60% of individuals are at moderate risk.^87^ In this scenario, adding the coronary calcium score to clinical risks has become the most efficient and cost-effective alternative to estimate the CAD risk in patients at moderate risk.

#### 3.2.5. Coronary Artery Calcium Score

Coronary artery calcium (CAC) is a highly specific characteristic of coronary atherosclerosis. The CAC score (CACS) is an available, consistent, and reproducible method to evaluate the risk for future coronary events, essentially by guiding primary prevention strategies.^90^ CACS in asymptomatic populations is cost-effective for moderate risk patients^90^ and has a positive impact on adherence to treatment.^91^

The Multi-Ethnic Study of Atherosclerosis (MESA) developed a valuable and useful support tool for CACS to predict risk, incorporating CACS to a clinical model using 10-year follow-up data until the first manifestation of CAD.^92^ The MESA score involves individuals aged 45 to 85 years, providing CAD risk in 10 years with and without CACS. The Heinz Nixdorf Recall (HNR) and the Dallas Heart Study validated the score.^92^ The greatest limitation of the MESA score is that its algorithm does not include all forms of atherosclerotic disease, which differentiates it from the ORS/SBC.^93^

In an analysis of patients from the MESA study^94^ who had an estimate of atherosclerotic cardiovascular disease (ASCVD) of 5 to 7.5% in 10 years, a CACS = 0 was associated with an ASCVD observed rate of 1.5%, while any calcium score > 0 was associated with an actual rate of events of at least 7.5%. In individuals from MESA with an ASCVD risk between 7.5 and 20%, a CACS = 0 was associated with an event rate of around 4.5%, while a CACS > 0 was associated with a net benefit of statin therapy of approximately 10.5%.

CACS should represent a way of segregating diabetic individuals with a higher atherosclerotic burden and possibly those suffering for longer the vascular effects of insulin resistance associated with endotheliopathy, which begins in the early stages of pre-diabetes.^72^

As explained above, pathophysiologically, vascular disease, especially diabetic coronary disease, starts long before its clinical diagnosis. However, the strategies to map the progression of the vascular disease in earlier stages are still limited, and there are few viable tools for clinical practice. Thus, a clinical score - such as the ORS/SBC - combined with CACS is the most efficient way to predict the CAD risk in moderate-risk patients.

#### 3.2.6. Lipid Targets in Primary Prevention for Individuals with Metabolic Syndrome and Diabetes Mellitus

Statins are among the most prescribed drugs worldwide, reflecting their fundamental role in primary and secondary prevention of atherosclerotic disease and the high prevalence of dyslipidemias. Several randomized clinical trials (RCT) and meta-analyses, such as the Cholesterol Treatment Trialists’ (CTT) Collaboration,^14^ solidified the indication of statins. Among 21 RCT comparing statin and placebo, with a total of 129,526 individuals followed for 4.8 years, each 40 mg/dL reduced of LDL-c decreased the incidence of CV events by 12% and CAD deaths by 20%. Moreover, the CTT analyses showed that a greater reduction in LDL-c with the use of more potent statins had an additive effect on the prevention of CV events. Findings of 5 RCTs with more than 39,000 individuals combined showed that reducing LDL-c levels in over 20 mg/dL with a more intensive lipid-lowering treatment can decrease the incidence of non-fatal myocardial infarction by 19%, ischemic CVA by 31%, and major CV events by 28%.

The use of statins in patients with CAD seems to stabilize atherosclerotic plaques, and can even lead to their volumetric reduction,^95^ with an approximately linear relationship between the decrease in LDL-c and the rate of CV events, as well as between LDL-c levels and the progression of the atheroma volume in carotid arteries. In parallel, not only the dose of statin and the reduction in LDL-c decrease CV risk, but the period of statin use also seems to have a central role in reducing the risk for CV death and non-fatal myocardial infarction. In the WOSCOPS study, for instance, the number needed to treat (NNT) with pravastatin after four years of follow-up was 40:1, whereas, after 16 years, NNT decreased to 27:1.^96^

Regarding lipid targets for patients in secondary prevention, the scenario was redesigned after the publication of the IMPROVE-IT study^97^ (with simvastatin and ezetimibe), whose LDL-c was 50 mg/dL, and the FOURIER study^98^ (alirocumab, a PCSK9 inhibitor), which reached mean LDL-c levels as low as 38 mg/dL. Based on the significant and consistent reduction in coronary events in two clinical trials, currently the LDL-c target is < 50 mg/dL; there is no reason, however, in terms of safety, to seek even lower targets, either through diet, statins, ezetimibe, or PCSK9 inhibitors.

In a primary prevention scenario, the reduction in vascular events is comparatively lower than in secondary prevention, but it still is robustly cost-effective in diabetic and non-diabetic patients with CV risk > 7.5% in 10 years.^99^ As revealed in the CTT meta-analysis, a decrease in LDL-c by 80 mg/dL (with a mean starting LDL-c from 130 to 160 mg/dL) combined with an effective statin regimen for about five years in 10,000 patients in primary prevention typically prevents 500 vascular events (5% of patients).^14^

Although the duration of clinical studies with statins is relatively short (3 to 7 years), patients with DM and MS will be subject to a metabolically unfavorable environment for the rest of their lives (10 to 30 years). Assuming that 68% of causes of death in diabetic patients are CV-related,^78^ it is reasonable to think that, once the high vascular risk is identified (based on the ORS with or without CACS), more aggressive therapeutic targets should be considered.

No RCT has investigated an LDL-c target below 70 mg/dL (JUPITER)^100^ in primary prevention. However, Mendelian randomization studies consistently support that lower LDL-c levels (including the 30-50 mg/dL range) were related to lower CV morbidity and mortality.^101^ Furthermore, a subanalysis of the JUPITER study showed that the lower the LDL-c level achieved (< 50 mg/dL), the greater the risk reduction in both diabetic and non-diabetic individuals.^102^

#### 3.2.7. Aspirin in Primary Prevention

The use of acetylsalicylic acid (ASA) in primary prevention is a controversial issue, but that seems to have recently reached a common denominator. In 2018, three RCT provided an answer to this question: the ASCEND,^103^ in diabetic patients; the ARRIVE,^104^ in non-diabetic patients at moderate CV risk (median risk of 15% in 10 years); and the ASPREE,^105^ in patients aged 70 years or older. All three studies compared low doses of aspirin (100 mg per day) with placebo from 5 (ARRIVE and ASPREE) to 7.5 years (ASCEND), and collectively found:

1. no difference in rates of myocardial infarction and acute myocardial infarction;
2. no difference in CV mortality;
3. no difference in all-cause mortality in ASCEND and ARRIVE, and a small risk increase with aspirin in ASPREE; and
4. greater risk for gastrointestinal malignancy among aspirin users in the ASPREE study (probably due to early diagnosis).

These data are consistent with a systematic review by the Antithrombotic Trialists’ Collaboration,^106^ which included 95,000 individuals from six RCT. The reduction in risk for vascular events ranged from 0.57 to 0.51% per year (placebo vs. aspirin), while extracranial and major gastrointestinal bleedings increased by 0.03% per year (0.10 to 0.07%).

Although observational studies suggest that the use of aspirin benefits primary prevention in patients at high CV risk,^107^ this result was not confirmed in subanalyses of ASCEND and ARRIVE. Even in patients at higher estimated risk for CV events, aspirin provided no net benefit since it induced more bleedings in this subpopulation, and the proportional decrease in vascular events was mild compared to that in individuals at lower risk.^103,104^

#### 3.2.8. Hypoglycemic Agents in Patients with Diabetes Mellitus

Despite the strong effect of glycemic control on microvascular complications among diabetic patients, its benefits for the macrovascular disease were still a paradigm until recently. Medicines such as sulfonylurea and insulin have limitations, despite being very effective in glycemic control, as they induce weight gain and increase the risk for hypoglycemia, two major risk factors for the worsening of symptoms and prognosis in HF and CAD. Several RCT tested these drugs, combined with metformin, by comparing intensive glycemic control and less aggressive targets. In a meta-analysis with 13 RCT and 34,533 diabetic individuals, although the risk for non-fatal myocardial infarction decreased with intensive glycemic control (relative risk - RR 0.85; 95% confidence interval, 0.74-0.96, p < 0.001), there was no significant change in all-cause mortality (RR 1.04; 99% confidence interval, 0.91-1.19) or CV mortality (RR 1.11; 95% confidence interval, 0.86-1.43).^108^

On the other hand, with the advent of new drugs that allow effective glycemic control associated with weight loss and minimal risk for hypoglycemia, the paradigm of glycemic control regarding CVD was broken. In a recent meta-analysis, GLP-1 analogs consistently reduced the incidence of CV deaths and non-fatal infarction by 14 and 18%, respectively.^109^ Data from the studies LEADER (liraglutide),^110^ SUSTAIN-6 (semaglutide),^55^ HARMONY (albiglutide),^111^ and REWIND (dulaglutide) demonstrated safety and efficacy among diabetic patients in secondary prevention and patients in primary prevention at high or very high CV risk. Chart 3.1 presents the recommendations for DM and MS management.

**Chart 3.1:** Recommendations for diabetes mellitus and metabolic syndrome management

Recommendation	Recommendation grade	Level of evidence	Reference
The Health ABC Heart Failure Score should be recommended for patients with MS or DM as a primary strategy in the risk stratification of HF	I	B	^52-54^
BNP values ≥ 50 pg/mL or NT-proBNP ≥ 125 pg/mL must be used together to reclassify individuals at moderate risk for HF into high risk Individuals at high and very high risk should receive an intensive primary prevention approach	IIa	A	^52-54^
Echocardiographic diagnosis of diastolic dysfunction in patients with DM or MS without clinical symptoms of HF should suggest an increased risk for the development of HF. However, the data available are not enough to recommend its routine use to estimate the future risk for symptomatic HF	IIA	B	^50,51^
The use of an SGLT2 inhibitor is recommended for patients with DM or MS without clinical symptoms of HF, but at high or very high risk for HF, based on the Health ABC Heart Failure Score and BNP levels	I	B	^57,58^
Prescribing rosiglitazone, pioglitazone, or saxagliptin is contraindicated for patients with DM or MS without clinical symptoms of HF, but at high or very high risk for HF, based on the Health ABC Heart Failure Score and BNP levels	III	A	^59-61^
Strategies for weight control, PA, dietary guidance, and quitting smoking should be offered to all patients with glucose intolerance, MS, or DM, so as to mitigate the progression of CAD	1	A	^75-77^
Stratifying the risk for coronary events with anatomical or functional methods is not recommended for asymptomatic patients with MS or DM	III	A	^78-84^
Using CACS is recommended for patients with DM or MS and at moderate CV risk (ORS 5 - 20%). When CACS = 0, the recommendation is usually not to start statin treatment	I	B	^89-94^
CACS should not be requested for patients with DM or MS and at low (ORS < 5%) or very high (> 20% in 10 years) CV risk	III	B	^14,95-97^
In primary prevention, patients with DM or MS referred to statin therapy should receive highly potent doses of these medicines and/or ezetimibe, with an LDL-c target < 70 mg/dL Alternatively, in individuals with DM or MS and at high or very high risk, the LDL-c target should be < 50 mg/dL	I I	A B	^14,95-97^
In primary prevention for patients with familial hypercholesterolemia, with or without DM or MS, the LDL-c target should be < 50 mg/dL, with an indication for a highly potent statin, ezetimibe, and PCSK9 inhibitors until the target is reached	I	A	^14,95-97^
Using ASA is not recommended as a primary prevention strategy for patients with MS or DM, regardless of CV risk	III	A	^103,104^
The introduction of a GLP-1 analog is recommended for diabetic patients with or without a history of CV disease, but at high or very high risk for ASCVD	I	A	^55,108-111^

ASCVD: atherosclerotic cardiovascular disease; BNP: brain natriuretic peptide; CACS: coronary artery calcium score; CAD: coronary artery disease; CV: cardiovascular; DM: diabetes mellitus; GLP1: glucagon-like peptide-1; HF: heart failure; LDL-c: low-density lipoprotein-cholesterol; MS: metabolic syndrome; ORS: overall risk score; SGLT2: sodium-glucose 2 cotransporter.

## 4. Obesity and Overweight

### 4.1. Introduction

In the past decades, Brazil underwent a process called nutritional transition^112^ - a concept related to secular changes in dietary patterns and nutritional status - and important modifications regarding food intake and PA patterns, as a consequence of economic, social, demographic, and health transformations.^113^ Obesity and overweight are complex and chronic conditions, whose prevalence has grown inexorably in the last 4 to 5 decades.^114^ Between 1980 and 2013, the global percentage of individuals with a BMI ≥ 25 kg/m^2^ rose from 28.8 to 36.9% in men and 29.8 to 38.0% in women.^115^ In Brazil, 52.4% of the population was overweight in 2014, with 17.9% of them classified as obese.^116^ According to data from the 2018 Risk Factors Surveillance and Chronic Disease Protection by Telephone Survey (VIGITEL), the incidence of overweight reached 55.8% and of obesity, 18.7% among men over 20 years of age; while for women, these values were 53.9% and 20.7%, respectively.^117^ In 34 years, the prevalence of obesity increased over four times for men (from 2.8 to 12.4%) and more than twice for women (from 8 to 16.9%).^118,119^ Brazil currently holds the fourth place among the countries with the highest prevalence of obesity and the number of overweight adults will exceed those with low weight.^118^ There is a significant rise in overweight and obesity among children and adolescents, regardless of gender and social status, and a considerable proportion of these individuals will become obese adults.

Obesity has a multifactorial nature and is one of the leading factors to explain the growth in the chronic NCD burden, given its frequent association with CVD, such as arterial hypertension (AH), CVA, HF,^120^ dyslipidemias, type 2 diabetes, atrial fibrillation,^121,122^ osteoarthritis, and certain types of cancer. Also, obesity is an important condition that predisposes the individual to mortality.^118,119^

In addition, weight gain over time is associated with MS, increased risk for CVA, and death in late stages of life.^123-125^ Many patients who present some of these changes have hypertriglyceridemia and increased levels of plasma fatty acids, stored as lipid droplets in the heart. Intramyocardial lipids that exceed the storage and oxidation capacity can become toxic and lead to non-ischemic and non-hypertensive cardiomyopathy, known as diabetic or lipotoxic cardiomyopathy.^126^ Significant weight loss (≥ 5% of initial weight) improves BP, LDL-c, TG, and glucose levels, delaying the onset of type 2 diabetes.^127^

### 4.2. Primary Prevention

According to the World Health Organization (WHO), an inadequate diet is the main risk factor for early mortality worldwide.^128^ Therefore, a healthy diet is recommended for everyone, and the ability to prepare healthy meals has beneficial correlations with the consumption of equally healthy foods.^129^ However, studies have shown a reduction in the habit of cooking in some countries, which has encouraged health specialists to elaborate nutritional education strategies focused on nutrients and tools, such as the proper purchase and storage of food, and planning and preparing meals at home.^130^

Also, we emphasize that the biological moment that could prevent weight gain is of the utmost importance. In females, the moment of greatest risk seems to be the reproductive age, specifically during pregnancy and the first two years postpartum, and the period post-menopause.^131,132^ Among children and adolescents, prevention of excessive weight gain was expected precisely because the growth phase requires extra energy, and the possibility of energy expenditure is higher compared to other life stages. These potential facilitators, however, do not seem to overcome the factors associated with obesity and those responsible for the epidemic growth also in these age groups and life stages.^133^ In this regard, we underline the so-called “obesogenic environment,” that is, the role of the food industry, fast food chains, advertisements, TV shows, movies, and videogames, leading to situations that keep the children more sedentary and subjected to excessive energy intake. The most appropriate interventions should combine environmental and behavioral changes.^134-136^

A study conducted with 422 adolescents, with a mean age of 12.5 years, compared students who practiced competitive physical activity daily for 2 hours with those from a standard school who have only one hour of physical activity per week. The percentage of overweight/obesity in the first group was 49.8% and in the second, 37.3%, which reveals the high prevalence of this change in the two groups.^137^ A similar sample submitted to a multidisciplinary program of moderate intensity, that could be easily incorporated in the daily routine, showed positive advances in risk factors when compared to the control group.^138^

Among adults, studies show a decline in the consumption of rice and beans, increase in the intake of processed products (particularly cookies and soft drinks), excessive consumption of sugar, more saturated fats, and insufficient intake of fruits and vegetables, creating an environment with habits that do not favor a healthy dietary pattern, directly associated with the increase in chronic NCD, especially obesity.^139-142^

A recommendation from the 2014 Dietary Guidelines for the Brazilian Population proposes 10 steps for a healthy diet:

1. Prioritize natural or minimally processed foods;
2. Use oil, salt, and sugar moderately;
3. Limit the consumption of processed foods;
4. Avoid the intake of ultra-processed foods;
5. Eat regularly and carefully;
6. Buy food at the street market;
7. Cook;
8. Plan the purchase of foods and preparation of meals;
9. Avoid fast food;
10. Be critical of food advertising.

Some other useful pieces of advice are:^143,144^

- Eat regularly throughout the day and at similar times every day to establish a healthy dietary pattern;
- Pay attention to food labels and choose products without trans and hydrogenated fats;
- Avoid soft drinks and processed juices, cakes, cookies, sandwich cookies, and sweet desserts;
- Give preference to drinking water between meals;
- Practice at least 30 minutes of vigorous physical activity on most weekdays or 40 minutes of moderate physical activity;
- However, individuals with a tendency towards obesity or with family profile should practice moderate physical activity for 45-60 minutes per day; those who were obese and lost weight should practice for 60-90 minutes to avoid regaining the weight lost;
- The practice of physical activities and exercises can prevent weight gain and obesity even in older adults.

Table 4.1 lists the recommendations on how to approach overweight and obese adults.

**Table 4.1:** Recommendations on how to approach overweight and obese adults

Recommendation	Recommendation grade	Level of evidence	Reference
Weight loss is recommended for overweight and obese individuals to improve their CV risk profile	I	B	^2,9,128^
Counseling and interventions addressing lifestyle, including caloric restriction, aimed at achieving and maintaining weight loss are recommended for overweight and obese adults	I	B	^2,9,128^
Calculate the BMI and anthropometric measures during medical appointments to identify overweight and obese adults with the purpose of intervention	I	C	^2,9,128^
Measure the waist circumference to identify individuals with higher cardiometabolic risk	IIa	B	^2,9,128^

BMI: body mass index; CV: cardiovascular.

## 5. Arterial Hypertension

### 5.1. Introduction

AH is the most prevalent chronic disease in the world, affecting approximately one-third of the adult population. BP is maintained by several factors, particularly the intravascular volume, cardiac output, peripheral vascular resistance, and the elasticity of arterial vessels. Among the various regulatory mechanisms, RAAS - involving the renal system - has significant participation; an imbalance in this complex regulatory system, however, can result in chronic elevation of BP levels, known as AH. AH is one of the most important CV risk factors, as hypertensive individuals present much more atherosclerosis, leading to CVA, HF, coronary disease, peripheral vascular insufficiency, and kidney disease.^145^

Although we have efficient drugs with few adverse effects, the worldwide control of this condition still leaves much to be desired, since we are dealing with completely asymptomatic disease, a fact that makes care adherence very difficult.

**Figure 5.1:**
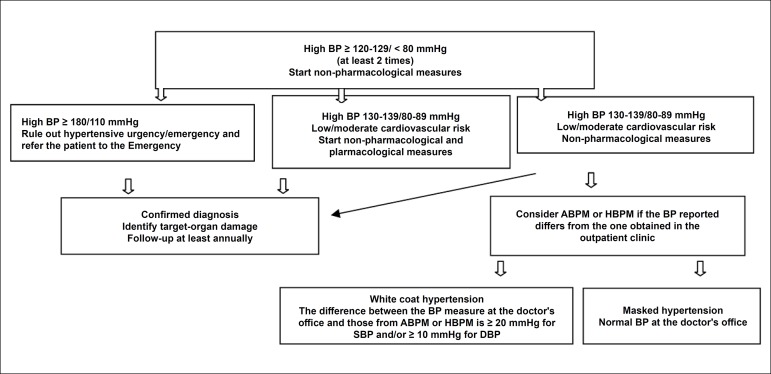
Flowchart for the diagnosis of arterial hypertension. BP: blood pressure; ABPM: ambulatory BP monitoring; HBPM: home BP monitoring. Modified from references.^9,10,189^

According to the 7^th^ Brazilian Guideline of Arterial Hypertension, an individual is hypertensive when his or her SBP and diastolic blood pressure (DBP) are equal to or higher than 140/90 mmHg (Table 5.1).^146^ Figure 5.1 shows the flowchart for the diagnosis of hypertension.

**Table 5.1:** Classification of blood pressure according to measurements taken casually or at the doctor's office in individuals aged 18 years and older^146^

Classification	SBP (mmHg)	DBP (mmHg)
Normal	≤ 120	≤ 80
Pre-hypertension	121-139	81-89
Stage 1 hypertension	140-159	90-99
Stage 2 hypertension	160-179	100-109
Stage 3 hypertension	≥ 180	≥ 110
When SBP and DBP are in different categories, the BP classification should assume the higher one		
Isolated systolic hypertension is determined when SBP ≥ 140 mmHg and DBP < 90 mmHg, and should be classified into stages 1, 2, or 3		

BP: blood pressure; DBP: diastolic blood pressure; SBP: systolic blood pressure.

The genesis of primary AH is multifactorial, with genetic and environmental influences. Although the genetic mechanisms involved are still obscure, there is evidence that children of hypertensive individuals have a greater chance of becoming hypertensive. However, the environmental aspect has an essential role in the development of AH. As the individual ages, the prevalence of AH increases significantly; therefore, detecting predisposing factors is important to prevent this critical CV risk factor properly. Besides family history, age, ethnicity, and insulin resistance, there are also environmental factors related to the development of AH that can be modified, such as obesity, psychosocial aspects, diet, sodium intake, sedentary lifestyle, and alcohol consumption.

### 5.2. Physical Activity and Hypertension

Epidemiological studies suggest that regular aerobic physical activity can be beneficial in preventing and treating hypertension, as well as reducing CV risk and mortality. A meta-analysis with 93 articles and 5,223 individuals showed that aerobic training, dynamic resistance, and isometric resistance reduce SBP and DBP at rest by 3.5/2.5, 1.8/3.2, and 10.9/6.2 mmHg, respectively, in the general population.^147^

Resistance training, but not other types of training, further reduces BP in hypertensive individuals (8.3/5.2 mmHg). Regular physical activity of lower intensity and duration reduces BP less than moderate or vigorous training but is associated with a decrease in mortality by at least 15% in cohort studies.^148,149^

This evidence suggests that hypertensive patients should be advised to practice dynamic aerobic exercise of moderate intensity (walking, running, cycling, or swimming) for at least 30 minutes 5 to 7 days per week. The practice of resistive exercises 2 to 3 days per week could also be recommended. Also, healthy adults could benefit from gradually increasing moderate aerobic physical activity to 300 minutes per week, vigorous aerobic physical activity to 150 minutes per week, or an equivalent combination of the two, ideally with supervised daily exercise.^6,9^ The impact of isometric exercises on BP and CV risk is less well established.

Table 5.2 demonstrates the classification of physical activity intensity and the levels of absolute and relative intensity. Table 5.3 shows the v goals to prevent and treat AH.

**Table 5.2:** Classification of physical activity intensity and examples of levels of absolute and relative intensity^9^

Classification	Absolute intensity		Relative intensity	
Intensity	MET	Examples	% HRmax	Talk test
Light	1.1 - 2.9	Cycling (< 4.7 km/h), light domestic chores.	50 - 63	
Moderate	3.0 - 5.9	Fast walking (4.8-6.5 km/h), slow-cycling (15 km/h), decorating, vacuuming, gardening, golf, tennis (in pairs), ballroom dancing, water aerobics.	64 - 76	Breathing is faster but compatible with complete sentences.
Vigorous	≥ 6.0	Running, cycling (> 15 km/h), heavy gardening, swimming, tennis.	77 - 93	Breathing is heavier, incompatible with a comfortable conversation.

Metabolic equivalent (MET) is the energy expenditure of an activity divided by the resting energy expenditure: 1 MET = 3.5 mL O_2_ kg^-1^ min^-1^ oxygen consumption (VO_2_). HR: heart rate; % HRmax: percentage of the maximum heart rate measured or estimated (220-age). Adapted from the 2016 European Guidelines On Cardiovascular Disease Prevention In Clinical Practice.^9^

**Table 5.3:** Physical activity to prevent and treat hypertension^147,151-153^

Intervention	Objective	Approximate impact of SBP	
		Hypertension	Normotension
Aerobic	• 90 to 150 min/week • 65 to 75% of HR reserve	-5/8 mmHg	-2/4 mmHg
Dynamic resistance	• 90 to 150 min/week • 50 to 80% 1 rep maximum • 6 exercises, 3 sets/exercise, 10 repetitions/set	-4 mmHg	-2 mmHg
Isometric resistance	• 4 × 2 min (handgrip), 1 min of rest between exercises, 30 to 40% of maximum voluntary contraction, 3 sessions/week • 8 to 10 weeks	-5 mmHg	-4 mmHg

### 5.3. Psychosocial Factors

Some psychosocial factors, such as work and family stress, depression, anxiety, hostility, and type D personality, as well as low socioeconomic and cultural status, increase the risk for AH - and consequently CVD - and reduce the adherence to a healthy lifestyle and drug treatment. On the other hand, CVD also increase the risk of manifesting these psychosocial factors, indicating a bidirectional and robust relationship.^154^

Moreover, the prevalence of CVD and AH is higher in developing countries, where the control rate of these diseases tends to be poor, decreasing life expectancy and increasing the pathologies and frailties related to aging.^155^ Several prospective studies and systematic reviews have addressed socioeconomic status, showing that low schooling and income, low-status jobs, as well as living in poor residential areas are associated with the increase in BP levels and consequently CV risk.^156,157^

Individuals with mood and personality disorders present an increase in the incidence and worsening of the prognosis of CVD, especially among those with depression or anxiety.^157^ Similarly, personality traits associated with hostility or distress also worsen the prognosis.^158^

The management of psychosocial stress with several existing techniques, among them meditation, music therapy, yoga, and slow breathing, can be crucial in preventing and controlling BP. In general, such techniques can mildly reduce BP levels in hypertensive individuals.^159,160^

### 5.4. Diets that Promote the Prevention and Control of Arterial Hypertension

In 2017, the Global Burden of Disease Group considered unhealthy diet as one of the main as risk factors for premature death and disability.^161^ Adjustments in the diet of individuals with normotension (NT) or pre-hypertension (PH) have the potential of reducing BP and preventing AH.^162^ National and international guidelines recommend that all patients with PH or AH reduce their sodium intake and consume adequate amounts of fresh fruit, vegetables, and low-fat dairy products.^163^ Furthermore, these documents emphasize the importance of maintaining body weight and waist circumference within the normal range.^164^

Many dietary patterns have been proposed to prevent and control AH, as well as maintain global and CV health. Among the dietary models proposed, with different levels of evidence and effectiveness to prevent and control AH, we highlight the Dietary Approaches to Stop Hypertension (DASH), low-fat, high-protein, low-carbohydrate, moderate carbohydrate, low-glycemic index/low-glycemic load, low-sodium, vegetarian/vegan, Mediterranean, paleolithic, Nordic, and Tibetan^165^ (Chart 5.1).

**Chart 5.1:** Methods and characteristics of dietary interventions proposed to prevent and control arterial hypertension

a. DASH: high consumption of vegetables and fruits, low-fat dairy products, whole grains, and low sodium intake
b. Mediterranean: high consumption of fruits, vegetables, olive oil, legumes, cereals, fish, and moderate intake of red wine during meals
c. Low-carbohydrate: < 25% of carbohydrates in the total energy intake; high consumption of animal and/or plant protein; in many cases, it has a high intake of fat
d. Paleolithic: lean meat, fish, fruits, leafy and cruciferous vegetables, tubers, eggs, and nuts, excluding dairy products, cereal grains, beans, refined fats, sugar, sweets, soft drinks, beer, and extra salt
e. Moderate carbohydrate: 25 to 45% of carbohydrates in the total energy intake; 10 to 20% of protein consumption
f. High-protein: > 20% of protein in the total energy intake; high consumption of animal and/or plant protein; < 35% of fat
g. Nordic: wholegrain products, plenty of fruits and vegetables, rapeseed oil, three fish meals per week, low-fat dairy products, no sugary foods
h. Tibetan: foods rich in protein and vitamins, preferably cooked and hot
i. Low-fat: < 30% of fat in the total energy intake; high consumption of cereals and grains; 10-15% of protein
j. Low-glycemic index: low-glycemic load
k. Vegetarian/vegan: without meat and fish/without animal products
l. Low-sodium: less than 2 g of sodium/day

Adapted from reference.^165^

A meta-analysis of 67 studies published between 1981 and 2016 compared the effects of these dietary patterns on patients with PH and AH. DASH, Mediterranean, low-carbohydrate, paleolithic, high-protein, low-glycemic index, low-sodium, and low-fat were significantly more effective in reducing SBP (-8,73 to -2,32 mmHg) and DBP (-4,85 to -1,27 mmHg) compared to the control diet.^165^

Regarding food supplements, several meta-analyses have evaluated the potential effects of additives on BP reduction with supplementation of certain substances in populations of individuals with NT, PH, and AH.^166^ The effects of these supplements on BP reduction are usually mild, heterogeneous, and their statistical significance is difficult to assess. The substances whose supplementation has evidence of significant BP reduction are: potassium, vitamin C, food-derived bioactive peptides, garlic, dietary fiber, linseed, dark chocolate (cocoa), soy, organic nitrates, and omega-3.^167^ Chart 5.2 shows the recommended mean daily portions, their potential impact on BP, the level of evidence, and the recommendation grade of each of these supplements, as well as other food interventions. Supplementation with calcium, magnesium, combined vitamins, tea, and coenzyme Q10 did not present a significant BP reduction.^168^

**Chart 5.2:** Dietary supplements and interventions with evidence of a potential reducing effect on blood pressure

Recommendation supplement or intervention SBP/DBP reduction	Recommendation grade	Level of evidence	Reference
Potassium: 90-120 mmol/day SBP/DBP= -5.3/-3.1 mmHg	IIa	A	^166^
Vitamin C: 500 mg/day SBP/DBP= -4.9/-1.7 mmHg	IIa	A	^166^
Bioactive peptides: 2.6-1500 mg/day SBP/DBP = -5.3/-2.4 mmHg	I	A	^166^
Garlic: 12.3-2400 mg/day SBP/DBP= -4.6/-2.4 mmHg	I	A	^166^
Dietary fiber: 11.5 g/day SBP/DBP= -2.4/-1.8 mmHg	I	A	^166^
Linseed: 28-60 g/day (crushed) SBP/DBP= -2.9/-2.4 mmHg	IIb	B	^166^
Dark chocolate: 46-100 g/day SBP/DBP= -2.9/-2.4 mmHg	I	B	^166^
Soybean: substituting 25g of dietary protein SBP -10%, DBP -7%	IIa	B	^166^
Organic nitrates: 15.5 ± 9.2 mmol +140-500 mL of beet juice/day SBP/DBP= -4.4/-1.1 mmHg	IIb	B	^166^
Omega-3: 3 to 4 g/day SBP/DBP= -4.5/-3.1 mmHg	I	A	^166^
Weight loss: - 5.8% / SBP/DBP= -4.4/-3.6 mmHg	I	A	^166^
Reduced alcohol consumption: - 67% / SBP/DBP= 3.9/2.4 mmHg	IIa	B	^166^

DBP: diastolic blood pressure; SBP: systolic blood pressure. Adapted from reference.^166^

### 5.5. Alcohol and Hypertension

The relationship between alcohol consumption and hypertension is known since 1915, when a pioneer publication reported this association.^169^ Several epidemiological studies corroborate the almost linear and dose-dependent relationship between alcohol and AH.^170^

The difficulty in determining the effect of alcohol on the development of AH is the difference in the quantification of the consumption pattern, and the varying alcohol concentration of these beverages. Heterogeneous results originate from the influence of the type of beverage ingested, volume consumed, lifestyle, intake pattern, and socioeconomic status of the population studied.^171-172^

The INTERSALT study evaluated the consumption of 300 ml of ethanol weekly (34 g, 3 or 4 drinks/day) and found a BP increase in drinkers compared to non-drinkers.^173^ Estimates indicate that excessive alcohol consumption is responsible for approximately 10-30% of AH cases.^174^ The ARIC study followed 8,834 individuals for eight years and, at the end of the investigation, the patients with high alcohol consumption had a greater incidence of AH, regardless of the type of beverage, gender, or ethnicity. Moderate alcohol consumption was associated with risk of developing AH, not only in African Americans but also in the Brazilian population.^175^ Approximately 6% of all-cause mortality worldwide is attributed to alcohol.^176^ When ingested in a single dose, alcohol has a dose-dependent biphasic effect characterized by BP reduction, vasodilation, and increase in HR with a subsequent BP elevation.^177^

In a study using the Ambulatory Blood Pressure Monitoring (ABPM) in pre-menopausal women, the group who consumed 20-300 ml of red wine/day (146-218 g of alcohol/week) showed a significant increase in BP.^178^ The same situation occurred in normotensive men who ingested an average of 40 g/day of ethanol, compared to the group who did not consume alcohol for four weeks.^179^

A meta-analysis with 15 RCTs, involving 2,234 participants, assessed the effects of reducing the consumption of ethanol on BP and estimated that a 2-mmHg reduction in DBP could decrease the prevalence of AH by 17%, the CAD risk by 6%, and the ischemic CVA and transient ischemic attack by 15%.^180^

### 5.6. Weight Loss and Prevention of Arterial Hypertension

Overweight is recognized as a factor related to BP elevation, and the greater the BMI, the higher risk for AH.^181^ Central obesity and weight gain over time stand out as important factors for the development of AH. The Nurses’ Health Study revealed that women who gained 5.0 to 9.9 kg and those who gained more than 25 kg in 18 years of follow-up had a higher risk for AH - 1.7 and 5.2, respectively. However, estimates suggest that only 26 to 40% of AH cases are attributable to overweight, emphasizing the multifactorial nature of AH.^182^

Weight loss as a non-pharmacological approach reduces BP in normotensive individuals and can prevent the development of AH. Changes in lifestyle are crucial for weight loss, focusing on the adoption of a hypocaloric diet and regular PA, with the reduction in caloric intake being more important than following specific diets.^183^

Regular isolated PA, without a concomitant dietary approach rich in fruits, vegetables, grains, seeds, nuts, fish, and dairy products, and poor in meats, sugars, and alcohol in general, is not enough for a significant weight loss.^184^

A meta-analysis of controlled studies with 4,184 individuals showed a reduction in SBP and DBP of 1.05 and 0.92 mmHg, respectively, for each 1 kg of weight lost. In healthy obese individuals, the combination of a low-calorie diet and BMI reduction was associated with an average decrease of 4.73/2.75 mmHg in SBP and DBP.^185^

A systematic review of studies with hypertensive subjects showed that the magnitude of BP reduction with weight loss was on average 4.5/3.2 mmHg for SBP and DBP, respectively, underlining that the greater the weight loss, the higher the BP reduction.^186^

The Framingham Study revealed a reduction in the risk of developing AH of 22 to 26% in individuals aged 30-49 and 50-65 years, respectively, who maintained a weight loss of 6.8 kg, in 8 years. In this context, regular PA stands as a measure of great importance in the maintenance of weight loss.^187^

### 5.7. Low-Sodium Diet in the Prevention of Arterial Hypertension

Prospective cohort studies have demonstrated that high sodium intake increases the risk of death and CV events. These studies also reported that decreasing sodium intake to below a certain value (approximately 3 g of sodium per day) further reduced BP. Paradoxically, low sodium intake was associated with an increased CV risk and risk of all-cause mortality in the general population and hypertensive individuals, suggesting a J-curve phenomenon. The mechanism of this apparent increased risk with low sodium intake is probably related to higher activity in the renin-angiotensin system under a very high restriction of salt in the diet. No epidemiological study has evidenced that very low sodium intake can be harmful.^10^

On the other hand, there is evidence of a causal relationship between sodium intake and an increase in BP. Excessive sodium intake (> 5 g of sodium per day) increases BP and is associated with a higher prevalence of systolic AH with aging.^188^

Many studies have shown that sodium restriction decreases BP. A meta-analysis revealed that a reduction of 1.75 g of sodium per day (4.4 g of salt/day) was associated with an average decrease of 4.2 and 2.1 mmHg in SBP and DBP, respectively, with a more pronounced effect in hypertensive individuals - 5.4 and 2.8 mmHg. The reducing effect of sodium restriction on BP is more significant in black people, older adults, and individuals with DM, MS, and chronic kidney disease (CKD).^164^

In Western populations, such as the Brazilian, the usual sodium intake is estimated between 3.5 to 5.5 g/day (which corresponds to 9 to 12 g of salt per day), with marked differences among countries or even regions.^189^

Sodium intake should be limited to approximately 2.0 g/day (equivalent to about 5.0 g of salt per day) in the population in general, but especially in hypertensive individuals.

The effective reduction of salt is not easy, and information about which foods have high levels of salt is often scarce. It is crucial that the population pay very careful attention to the amount of salt added to meals and with foods high in salt (processed products). Reducing salt intake remains a public health priority, but it requires a combined effort between the food industry, governments, and the general population since 80% of the salt consumed originates from processed foods. The adequate consumption of fruits and vegetables enhances the beneficial effect of a low-sodium diet on BP, mainly due to the increased intake of potassium, known for reducing BP.

It is possible to prevent or postpone AH with a change in lifestyle, which can effectively promote the primary prevention of systemic arterial hypertension (SAH), especially in individuals with borderline BP.^10^ Healthy lifestyle habits should be adopted since childhood and adolescence, respecting the regional, cultural, social, and economic characteristics of individuals (Chart 5.3).

**Chart 5.3:** Recommendations on how to approach adults with high blood pressure or arterial hypertension

Recommendation	Recommendation grade	Level of evidence	Reference
Non-pharmacological measures are indicated for all adults with high BP or hypertension to reduce BP: weight loss, healthy eating habits, low sodium intake, dietary potassium supplementation, increased physical activity with a structured training program, and limited alcohol consumption	I	A	^9,10,155,164,189^
Antihypertensive drugs are recommended for adults at estimated risk ≥ 10% in 10 years and average SBP ≥ 130 mmHg or average DBP ≥ 80 mmHg, for primary prevention of CVD	I	A	^9,10,155,164,189^
A BP target < 130/80 mmHg is recommended for adults with confirmed hypertension and CV risk ≥ 10%	I	B	^9,10,155,164,189^
A BP target < 130/80 mmHg is recommended for adults with arterial hypertension and chronic kidney disease	I	B	^9,10,155,164,189^
A BP target < 130/80 mmHg, which should start if BP ≥ 130/80 mmHg, is recommended for adults with arterial hypertension and type 2 diabetes	I	B	^9,10,155,164,189^
Antihypertensive drugs are recommended for adults at estimated risk < 10% in 10 years and average BP ≥ 140/90 mmHg for primary prevention of CVD	I	C	^9,10,155,164,189^
In adults with confirmed hypertension, without additional markers of increased CV risk, the recommended BP target is < 130/80 mmHg	IIb	B	^9^^.^^10,155,164^^,^^189^

BP: blood pressure; CVD: cardiovascular disease; DBP: diastolic blood pressure; SBP: systolic blood pressure.

### 5.8. Antihypertensive Control in Primary Prevention of Diabetes Mellitus and Metabolic Syndrome

BP control is one of the more robust tools for reducing CV risk. Reducing 20 mmHg in SBP can decrease CAD mortality by 40%, CVA mortality by 50%, and HF mortality by 47%. However, AH is still the most common and potent risk factor for loss of life expectancy, due to the suboptimal population control of this condition.^190-192^

Based on the non-automated measurement taken in the doctor’s office, the recommended BP target is < 130/80 mmHg for individuals with stages 1 and 2 hypertension at low and moderate CV risk, and those with stage 3 hypertension at low, moderate, and high CV risk.^146^ This recommendation is based on meta-analyses of randomized studies,^193,194^ which demonstrated the superiority of this BP target compared to values above 150/90 mmHg. Decreasing this target to 130/80 mmHg seems to be safe in this lower-risk population, as observational^195^ and some randomized studies corroborate,^194,196^ although the additional benefit is relatively small and counterbalanced by the risk for symptomatic hypotension and adverse effects of drugs.

On the other hand, individuals with stages 1 and 2 hypertension at high or very high CV risk or with three or more risk factors, and/or MS, and/or target-organ damage should have BP levels < 130/80 mmHg.^146^ In the SPRINT study,^197^ among the 9,361 non-diabetic individuals at high CV risk (median of 24.8% in 10 years), 39% met the criteria for MS. The study population was randomized for a more (< 120 mmHg) and less intense (< 140 mmHg) reduction in SBP - automated BP measurement (on average, 10 mmHg lower than the SBP measured at the doctor’s office with a non-automated method). Among patients with MS, the reduction in the primary outcome - comprising acute coronary syndromes, CVA, HF, or CV death - was similar to that of patients without MS after 3.26 years of follow-up. The most intense SBP treatment arm presented a decrease of 25% in the risk of primary outcome compared to that with less intense reduction (HR 0.75; 95% confidence interval: 0.57-0.96; p < 0.001).^198,199^

Among patients with coronary disease, the recommended BP target should be between 130 x 80 and 120 x 70 mmHg, particularly avoiding a DBP below 60 mmHg due to the risk for coronary hypoperfusion, myocardial damage, and CV events.^146^ A J curve has been consistently identified in this population, with SBP < 120 mmHg and DBP < 70 mmHg being associated with higher mortality.^200^

## 6. Vitamins and Omega-3 Fatty Acids

### 6.1. Introduction

Several observational studies have found a strong association between the consumption of grains, fruits, and vegetables - foods rich in vitamins and minerals - and low CV mortality^201^ and lower risk for myocardial infarction.^202^ Given this strong evidence, numerous intervention studies have tested the impact of supplementation with micronutrients (vitamins) and certain fatty acids (omega-3 series) on primary and secondary prevention of CV events. From a practical point of view, most of these studies showed no clinical benefit related to supplementation in the doses studied and in the face of the drug therapies used to prevent CV. Tables 6.1. to 6.3 summarize the recommendations for and against the use of these supplements.

**Table 6.1:** Summary of the recommendations for the non-consumption of vitamin supplements to prevent cardiovascular diseases

Recommendations	Description	Recommendation grade	Level of evidence	References
Vitamin A or beta-carotene	There is no evidence of the benefit of vitamin A or beta-carotene supplementation for primary or secondary prevention of CVD	III	A	^204,205^
Vitamin B and folic acid supplements	They are not effective in preventing primary or secondary CVD	III	A	^164,208^
Vitamin D	Vitamin D supplementation is not recommended to prevent CVD in people with normal blood levels for this vitamin. Similarly, there is no evidence that its supplementation in individuals with deficiency will prevent CVD	III	A	^215,216,217^
Vitamin E	Vitamin E supplementation is not recommended to prevent CVD	III	A	^205,208^
Vitamin K	In the same way, there is no evidence that vitamin K supplementation, in its different forms, can prevent CVD	IIa	C	^219,220^

CVD: cardiovascular disease.

**Table 6.3:** Recommendation for the consumption of foods rich in omega-3 fatty acids of plant origin

Indication	Class	Level of evidence	References
Stimulating the consumption of omega-3 polyunsaturated fatty acids of plant origin as part of a healthy diet can be recommended to reduce the CV risk, although the real benefit of this recommendation is debatable, and the evidence is inconclusive	IIb	B	^238^
ALA supplementation is not recommended to prevent CVD	III	B	

ALA: alpha-linolenic acid; CV: cardiovascular; CVD: cardiovascular disease.

### 6.2. Carotenoids

Carotenoids are a class with over 600 compounds, responsible for the yellow, red, and orange pigments in plants, with a-carotene, b-carotene, b-cryptoxanthin, lycopene, lutein, and zeaxanthin being the most commonly found in food. Known primarily as precursors of vitamin A, carotenoids are also essential suppressors of free radicals and act as potent antioxidants.^203^ The evidence for the role of carotenoids in CVD originated from studies showing that increased consumption of fruits and vegetables was associated with a lower risk for CVD.^204^ A series of retrospective and prospective longitudinal studies identified an inverse association between carotenoid intake and risk for CVD.^204^ However, the effect of carotenoids is complex and probably does not result from a single isolated compound. In contrast, prospective randomized studies showed no benefit of carotenoid supplementation for CVD.^204,205^ Corroborating this information, a cross-sectional analysis, consisting of 894 members of the cohort study Kardiovize, revealed that the consumption of foods containing vitamins (carotene, zinc, selenium, and vitamins A and C) was associated with a reduction in the intimal thickening of the carotids in women.^206^ In this research, the authors developed a “dietary antioxidant index” to categorize the foods, excluding individuals who used antioxidant supplements. Therefore, the use of supplements only with carotenoids, b-carotene, or similar compounds is not recommended. Instead, efforts should be directed toward increasing the consumption of fruits and vegetables rich in these nutrients.

### 6.3. Vitamin E

Vitamin E is the main fat-soluble antioxidant in the human body and is present in a complex of four isomers (a-, b-, g-, and d-tocopherol). The interest in the potential benefit of vitamin E for risk of CVD was related to its antioxidant capacity and the possibility of modifying oxidized low-density lipoprotein (Ox-LDL), particularly involved in atherogenesis.^207^ However, prospective randomized studies, such as the ATBC, CHAOS, GISSI, and HOPE, showed no benefit of vitamin E supplementation for CVD.^205,208^ The effect of supplementation with vitamin E and vitamin C on alternate days for eight years on 14,641 individuals did not reduce the incidence of myocardial infarction, CVA, and CV mortality, in addition to being associated with an increased incidence of hemorrhagic CVA.^209^ Despite the solid molecular basis theory of oxidative stress and its role in atherosclerosis, these clinical trials do not corroborate the use of vitamin E supplementation to prevent CVD. On the other hand, consuming foods containing vitamins E, A, and C was associated with a lower risk for adverse CV outcomes, as demonstrated in the Hong Kong Cardiovascular Risk Factor Prevalence Study (CRISPS), a longitudinal study comprising 875 participants.^210^ Thus, the consumption of foods with vitamin E has proven to be more effective and safe, and vitamin E supplementation is not recommended to prevent CVD.

### 6.4. Vitamin D

Vitamin D is an important precursor of the steroid hormone calcitriol, which is crucial for mineral and bone metabolism. In addition, it has other functions and supplementation with this vitamin to prevent and treat a wide range of diseases has increased considerably in the past decade.^211^ Its two main forms are vitamin D2 (ergocalciferol) and D3 (cholecalciferol). Vitamin D3 can be synthesized by human skin cells after exposure to UV-B radiation from sunlight. In the absence of sunlight, the intake of vitamin D is crucial. Vitamin D and dietary supplements are absorbed by the intestine and then converted into 25-hydroxyvitamin D3 [25(OH)D] in the liver, and 1.25 dihydroxyvitamin D3 [1.25(OH)2D3], the active form of vitamin D, in the kidney. Zittermann et al.^212^ summarized the underlying mechanisms for the potential role of vitamin D in preventing coronary disease. They include inhibiting the proliferation of vascular smooth muscle, suppressing vascular calcification, down-regulating pro-inflammatory cytokines, up-regulating anti-inflammatory cytokines, and acting as a negative endocrine regulator of the renin-angiotensin system. Low concentrations of circulating vitamin D were associated with AH, obesity, DM, and MS; furthermore, observational studies associated the deficiency of this vitamin with the risk for CVD.^212,213^ Some ecological studies suggest that vitamin D has a role in CVD, showing an increase in cardiac disease events according to the geographical latitude, that is, associated with lower exposure to solar radiation, with the concentration of vitamin D decreasing with latitude. Several prospective studies have investigated the association between plasma concentration of 25-hydroxyvitamin D and CVD, indicating an inverse relationship between the concentrations of this vitamin in the blood and the risk for CVD.^213,214^ Despite this evidence, data from a systematic review conducted by Beveridge et al.^215^ showed a lack of consistent benefit of vitamin D supplementation for the main markers of endothelial and vascular function.^215^ A randomized, controlled, double-blind study that lasted 5.3 years tested the efficiency of daily supplementation with 2,000 IU of vitamin D3 (cholecalciferol) in 25,871 participants.^216^ The primary outcomes assessed were myocardial infarction, CVA, and mortality from all CV causes, in addition to secondary outcomes of CV events. Vitamin D supplementation did not result in a lower incidence of CV events compared to placebo. The ViDA (Vitamin D Assessment) trial involved 5,108 participants in New Zealand aged 50-84 years. In the treatment group, participants received an initial dose of 200,000 IU followed a month later by 100,000 IU or placebo for an average of 3.3 years. The study found no significant reduction in CVD and mortality in the group that received vitamin D in comparison with the placebo group.^217^

Although observational studies demonstrate a positive association between low concentrations of 25hydroxyvitamin D and the risk for CV events, its supplementation is not indicated to prevent CV at the moment. However, studies with an adequate design still need to prospectively investigate populations with prominent deficiencies, especially patients with CKD, and other doses of this vitamin.^218^

### 6.5. Vitamin K

The review prepared by the Cochrane Library could not assess the effectiveness of vitamin K supplementation in decreasing all-cause mortality, including CV and non-fatal outcomes (myocardial infarction, CVA, and angina), in depth because only one study met the pre-established inclusion criteria.^219^ This study comprised 60 individuals aged 40-65 years investigated for three months and revealed that vitamin K2 did not change their BP and concentration of plasma lipids. The very limited results of this review highlight the lack of robust data about the efficiency of vitamin K in the primary prevention of CVD. However, the authors declared that the evidence for this assertion was minimal.

A recent systematic review and meta-analysis, registered as the PROSPERO study, analyzed the results of 13 clinical trials that evaluated the effects of vitamin K supplementation on cardiometabolic risk factors in healthy individuals or a population at high risk for CVD. The study found no benefit for plasma lipids, inflammatory cytokines - such as CRP and interleukin-6 - SBP, and DBP, both in healthy individuals and among those at CV risk.^220^ Therefore, the literature has no evidence to recommend vitamin K for CV prevention.

### 6.6. Vitamin C

Vitamin C or ascorbic acid is soluble in water and a very effective antioxidant since it loses electrons easily. The free radical theory of the aging process clarifies its role in the progression of chronic diseases.^207^ The Japan Collaborative Cohort Study (JACC)^221^ assessed food intake in 23,119 men and 35,611 women aged 40 to 79 years without a history of CVD, and showed that the consumption of foods rich in vitamin C was inversely associated with mortality from CVD in Japanese women. Despite the beneficial effects of consuming foods rich in vitamin C shown in observational studies, RCTs do not confirm the efficiency of its supplementation in primary or secondary prevention of CVD.^222^ Consequently, vitamin C supplementation is not recommended to prevent CVD.

### 6.7. B Vitamins and Folate

Evidence of a connection between vitamin B and CVD was demonstrated by the effect of these vitamins on the reduction of homocysteine.^223,224^ Homocysteine, an amino acid containing sulfur, is a metabolite produced indirectly in the demethylation of methionine. Prospective studies have shown an independent but modest association between plasma concentrations of homocysteine and the risk for CVD.^223^ Some factors identified as associated with high concentrations of homocysteine are: inadequate intake of folic acid and vitamins B6 and/or B12; for this reason, the growth in plasma concentrations of homocysteine can only be one follow-up marker of an inadequate diet. Other factors that might be associated with increased homocysteine include: preexisting atherosclerotic disease, consumption of coffee and alcohol, smoking, DM, use of antiepileptic drugs or methotrexate, renal failure, rheumatoid arthritis (RA), hypothyroidism, and cystathionine beta-synthase and methylenetetrahydrofolate reductase mutations. Prospective randomized studies with a large number of CV events failed to show any benefit of folate and B complex supplementation in reducing homocysteine and preventing CVD.^208^ The disagreement between the results of epidemiological studies and clinical trials might be partially due to the inclusion of different populations and the use of folic acid-fortified foods in some countries. Folic acid or B complex supplementation is not recommended to prevent CVD.^224^

A recent observational study conducted in 195 countries reiterated the efficiency of consuming foods containing vitamins to prevent CV risk and mortality, associating the mortality rate of CVD attributed to diet with low intake of fruits, grains, and vegetables.^164^ In conclusion, based on current evidence, a diet rich in vitamins must be encouraged; however, there is no indication that supplementation with these compounds can prevent CV events.

### 6.8. Omega-3 Polyunsaturated Fatty Acids of Marine Origin (Docosahexaenoic Acid and Eicosapentaenoic Acid)

Omega-3 fatty acids of marine origin - docosahexaenoic acid (DHA) and eicosapentaenoic acid (EPA) - produce numerous effects on different physiological and metabolic aspects that can influence the chance of developing CVD.^225,226^ Despite the general agreement that regular consumption of fish rich in omega-3 fatty acids is part of a healthy diet, recommending dietary supplementation with fish oil capsules is controversial, fostered by conflicting results of clinical studies.^32,227-229^

### 6.9. Effects of Omega-3 on the Lipid Profile

Clinical studies show that 2 to 4 grams of EPA/DHA supplementation per day can reduce TG levels by up to 25 to 30%, slightly increase HDL-c (1 to 3%), and raise LDL-c by 5-10%.^32^ The ability to decrease TG levels depends on the dose, with a reduction of approximately 5 to 10% for each 1 g of EPA/DHA consumed per day, which can be higher in individuals with greater baseline TG concentrations. These data show that high doses of omega-3 supplementation can be used to treat hypertriglyceridemia.

### 6.10. Omega-3 and Cardiovascular Outcomes

In a meta-analysis of 36 RCT, supplementation with fish oil (median dose of 3.7 g/day) reduced SBP by 3.5 mmHg and DBP by 2.4 mmHg.^230^ The decrease in adrenergic tonus and systemic vascular resistance is a proposed mechanism. Although several old pieces of evidence suggest a protective effect of fish and omega-3 fatty acids of marine origin on CV events,^230^ particularly in individuals with established CVD, more recent studies, showed no benefit of omega-3 supplementation for subjects who had or had not presented manifestations of atherosclerotic disease.^227,228^ In fact, a meta-analysis of 10 studies involving 77,917 individuals both in secondary (64% with prior coronary disease, 28% with prior CVA) and primary prevention (37% of diabetic individuals) failed to show any benefit of omega-3 supplementation (EPA doses ranging from 226 to 1,800 mg/day and DHA from 0 to 1,700 mg/d) after a mean follow-up of 4.4 years, presenting 6,273 coronary events (2,695 coronary deaths).^231^ These results were confirmed in an extensive systematic review and meta-analysis by the Cochrane group, with more than 119,000 individuals from 79 randomized studies.^232^ Also, the same meta-analysis did not find the benefit of supplementation with alpha-linolenic acid (ALA), the plant omega-3. Possible reasons for the divergent results between old and contemporary studies concern the profile of the population studied, mainly regarding the more frequent use of medicines known as protectors (e.g., statins, beta-blockers, ACEI), the more aggressive control of traditional risk factors, and the higher number of myocardial revascularization procedures in more recent studies. Another difficulty in analyzing studies with omega-3 supplementation is the diversity in its composition and the lack of control regarding the intake of omega-3 in the diet.

More recently, two published clinical trials used low doses (up to 1 g/day of EPA + DHA) of omega-3 in primary prevention of CVD. One of them has assessed the role of omega-3 in the primary prevention of CVD and cancer among men over 50 years of age and women over 55 years of age (VITAL study).^233^ Using a formulation containing 460 mg of EPA and 380 mg of DHA, the study included 25,871 patients with a median follow-up of 5.3 years and found no benefit of omega-3 in reducing major CV event or invasive cancer.^233^ Another study on primary prevention, but in diabetic patients, also examined the combination of EPA/DHA in the same composition of the VITAL study. It included 15,480 diabetic patients followed for an average of 7.4 years and found no benefit of omega-3 in reducing major vascular event.^234^ Thus, the role of omega-3 fatty acids in the doses used with respect to the primary prevention of CV events is questionable.

The Reduction of Cardiovascular Events with Icosapent Ethyl-Intervention Trial (REDUCE-IT)^33^ tested omega-3 in the reduction of CV outcomes among patients with hypertriglyceridemia and established CVD or diabetic patients with an additional risk factor. The patients received highly purified EPA (icosapent ethyl) at a dose of 4 g/day. The study included 8,179 patients who used statins and had TG ranging from 135 to 499 mg/dL (median of 216 mg/dL) and a median LDL-c of 74 mg/dL. The median reduction was 18% for TG and 6.6% for LDL-c in the EPA group. REDUCE-IT showed a relative decrease of 25% in composite CV outcomes among patients who received EPA and an absolute risk reduction of 4.8%, NNT of 22 patients to prevent an event. The hierarchical analysis demonstrated a significant decrease of 20% in CV mortality. On the other hand, the risk of hospitalization for atrial flutter or fibrillation presented a relative increase of 67% (1% absolute) with the treatment. The reduction of events in the REDUCE-IT study is similar to the results of the Japan EPA Lipid Intervention Study (JELIS), in which 1.8 g/day of EPA also led to a significant decrease in CV events among individuals who already used low doses of statins.^227^ However, the results of this last study are limited by their open design and lack of a placebo group.

Data from these studies suggest that high doses of EPA (4 g) can be used in patients with prior CVD and who remain with elevated TG levels, despite taking statins to prevent CVD. However, there is no evidence for the use of lower doses and other formulations of omega-3 for CV prevention, both primary and secondary. We emphasize, however, that several studies are still testing moderate to high doses of EPA and EPA + DHA in individuals at high risk for CVD who present persistent moderately high TG.

### 6.11. Omega-3 in Heart Failure

The GISSI-Heart Failure (GISSI-HF) trial evaluated the role of omega-3 in HF.^235^ This study randomized patients with chronic HF functional class II-IV of different etiologies to receive 1 g of omega-3 (EPA + DHA) (n = 3,494) or placebo (n = 3,481) per day.^235^ The primary outcome was time to death and time to death or hospitalization for CV causes. During a median follow-up of 3.9 years, the omega-3 group presented lower mortality rate (27 versus 29%, HR 0.91, 95% confidence interval 0.83-0.99, p = 0.041, with NNT = 56) and lower incidence of primary outcome (57 versus 59%, HR 0.92, 95% confidence interval 0.84-0.99, p = 0.009, with NNT = 44).^235^ Data from this study, however, need to be confirmed.

### 6.12. Omega-3 Polyunsaturated Fatty Acids of Plant Origin

ALA has shown inconsistent effects on lipid levels.^236,237^ A systematic review and meta-analysis of 14 randomized controlled trials with ALA supplementation found no significant influence on TC, LDL-c, or TG and only a minimal effect on HDL-c (a 0.4 mg/dL reduction).^238^

Specifically, the effects of linseed on experimental animals range from zero to a slight lipid decrease, and a review suggested a reducing impact on TG due to humans consuming large amounts of linseed oil.^238^ Observational studies indicate a modest reduction in the risk for CVD with the consumption of ALA.^238^ Data from the Alpha-omega study showed no benefits of ALA supplementation in preventing CVD among individuals who had a prior CVD. More recent data from the Cochrane group meta-analysis suggest that increasing the intake of ALA probably makes little or no difference in all-cause mortality, CV mortality, and coronary events.^232^ Its effects on CVA are unclear. However, the authors recognize the low quality of most studies; therefore, further studies on ALA supplementation are necessary to prove or refute its effectiveness in preventing CVD. We can conclude that, at the moment, there is no evidence to recommend ALA supplementation to prevent CVD. Table 3.3 presents the recommendations for ALA consumption and supplementation.

## 7. Smoking

### 7.1. Introduction

Smoking control in Brazil has been considered as a model, not only for its programming, but for the results, with tobacco use being reduced by at least half when compared to the last decades of the 1900s. Increased cost-effective taxes, a ban on advertising, a ban on indoor use (smoke-free laws), the sale of tobacco products to minors, discussion of the subject in the school curriculum, and warnings and information about Harmful effects of smoking on schools, universities, the media, and on cigarette packs themselves were effective measures to reduce smoking. There are currently over one billion smokers in the world. Brazil is considered one of the countries in the world that has most reduced the prevalence of smokers in the last thirty years. In 1989, about 32% of the population over the age of 15 were smokers, according to the National Health and Nutrition Survey of the Brazilian Institute of Geography and Statistics (IBGE). In 2018, the VIGITEL Survey of the Ministry of Health, through a telephone survey in 27 Brazilian cities, found a frequency of adult smokers over 18 years of age of 10.1%, being higher in males (13.2%) and lower in females (7.5%). The highest frequencies were found in men between the cities of Curitiba and Porto Alegre (17.3%) and São Paulo (15.6%).^239^ For the CV system, continued long-term use of tobacco and its derivatives leads to the onset of chronic diseases, which will manifest around 30 years after the start of regular smoking. As most smokers become addicted to nicotine before age 18, the health consequences are disastrous given the long exposure of the body to the harmful components contained in cigarettes. Therefore, prevention is the key to this health catastrophe.

**Smoking Prevention:** Despite worldwide tobacco control efforts, the number of nicotine addicts is still substantial. Effective public policies against the sale, advertising and use of cigarettes in public areas are important tools in primary prevention, as they prevent non-smokers, especially children and adolescents, from being exposed to nicotine. Therefore, the experimentation rate should be reduced in young people under 15 years of age. This initiative will surely reduce the number of potential tobacco users.

**Primordial prevention of smoking:** “Primordial prevention of smoking” is understood to be established before the initiation of smoking. It first identifies the risk factors for smoking, with a wide range from the individual’s own vulnerability, such as the surrounding social determinants. Providing a personalized and continuing education and enabling information exchange requires the formation of a multidisciplinary team.^240^ The aim of this team is to work with individuals and their families on the risks of smoking, to outline strategies to prevent them from smoking and to promote health.^241,242^

Factors that contribute to starting smoking:

1. ***Attitudes and beliefs:*** a study of adolescents^243^ showed that 40% of those who had never smoked became experimenters and that 8% had a smoking habit for 4 consecutive years.^244^ The lack of a firm decision not to smoke was the strongest predictor of experimenting;
2. ***Influence of friends and family:*** the presence of smokers among family and friends is an important predictor of tobacco initiation during adolescence; refusing a cigarette in the face of social pressure is a challenge for most teenagers, and only 44% of them can refuse a cigarette at a party;^245^
3. ***Age:*** both positive and negative attitudes towards smoking are more pronounced in adolescence;^246^
4. ***False conception:*** adolescents tend to overestimate the frequency of smoking among adults^247^ and to underestimate their own;^248^
5. ***Advertinsing:*** magazines and movies are main advertising sources promoting adolescent smoking;
6. ***Nicotine dependence:*** nicotine is a highly addictive substance, and many individuals develop dependence after a few days or weeks of exposure.^249^ Young people are more vulnerable than adullts to nicotine dependence;^250^
7. ***Other risk factors:***
  - *Depression:* the majority of studies show a relation between the presence of depression and the initiation of smoking, allthough it is not clear if the association is causal;
  - *Poor school performance:* missing classes and poor school performance are associated with initiation and continuation of smoking;^251,252,253^
  - *Adverse experiences:* separation of parents or divorce, physical emotions; emotional physical, or sexual abuse; growing up among family members who are addicted, mentally ill or imprisoned;^254,255^
  - *Substance abuse:* there is a high frequency of smokers among adolescents who use illicit drugs,^256^ so every adolescent who smokes should be considered potentially engaged in other risky behaviors.

### 7.2. Strategies in Combating Smoking Initiation ^257,258^

One way to approach primordial prevention is by age groups, observing for each group five main items (5 As):

**Group 0 to 4 years:** Ask parents and other family members about their smoking habits; advise to keep the environment free of cigarette smoke; the message should include information about the risks to parents and children, as well as the importance of the parental model; assess willingness to cooperate between parents and other family members; assist parents with trying to quit by informing them about self-help material and/or referring them to their own doctors; make an appointment with clinic within 3 months if a relative is a smoker; check parental progress at each subsequent pediatric visit.

**5 to 12 years old group:** Ask children about how they feel when someone is smoking nearby and what they do about it if they think it is dangerous to try smoking and if they think they will smoke when they are older, if they have tried smoking or if they have friends who smoke; advise children not to try smoking, praise them for remaining a non-smoker and/or staying away from cigarette smoke; remind children about the short-term negative effects of tobacco, such as reduced ability to smell and athletic capacity, as well as personal health risks (e,g., asthma exacerbation); advise parents to quit smoking and to give clear anti-smoking information to their children; assess the risk factors of smoking initiation or regular smoking progression, including level of experimentation, smoking among friends, depressive symptoms, school performance and adverse experiences; help parents in trying to quit smoking; assist children with developing skills to refuse smoking and exposure to it; assist parents in efforts to prevent tobacco use by their children through parenting and firm anti-smoking messages; make an appointment with clinic within 1-2 months for any child who is smoking or has worrisome risk factors for smoking, refer as necessary cases of social or learning difficulties and mental disorders as well.

**Group of adolescents and young adults:** ask adolescents about smoking behavior, confidentially about friends who smoke and about light cigarettes; advise adolescents to stop smoking, reinforcing personal health risks and danger of addiction; commend adolescents who are not smoking and remind them about the health risks; assess the motivation and symptoms of tobacco dependence among adolescents who are smoking; assess risk factors for smoking initiation among non-smokers; assist adolescents who are smoking with trying to quit, including nicotine replacement and referring if necessary; help parents with their efforts to prevent smoking initiation among their children through parenting and firm anti-smoking information; make an appointment with clinic within one month for each teenager who is smoking, supporting attempts to quit or assessing motivation and barriers to quitting; refer as necessary if risk factors are identified, such as social or learning difficulties, or findings of mental disorders.

Primordial Prevention of CVD, in its broadest context, it involves avoiding the establishment of modifiable CVD risk factors, including smoking, and effective strategies for promoting CV health of the individual and the population. Therefore, the joint action of interdiscipline teams (doctors, nurses, psychologists, physical educators, educators, nutritionists, social workers, communicators, managers) and intersectoral (family, school, government, society specialists, university) teams is necessary in a continuous and simultaneous way.

### 7.3. How to Treat Smoker’s Psychological Dependence

Nicotine addiction is a highly complex process that should be addressed by all health professionals. Every healthcare professional, especially the doctor during consultations, as well as the multidisciplinary team, should ask if the patient is a smoker. This question is essential. If the patient is a smoker, two types of approach can be used.

Basic approach where the goal is to ask if you smoke, to evaluate the smoker’s profile, to advise to quit smoking, to prepare for cessation and to accompany the smoker to the cessation of smoking. This approach should always be performed by the physician during the routine consultation, with an average duration of 3 (minimum) to 5 (maximum) minutes with each contact the patient makes. The patient should be questioned and asked systematically at each consultation and feedback on the evolution of the cessation process. Suitable for all smokers. Meta-analysis involving 29 studies showed that cessation rates were 19.9% for those who underwent medical intervention.^259^

**Specific Intensive Approach:** performed by health professionals available and trained to make a more in-depth follow-up with the patient, including the doctor. In this case, the professional should have a structured program available to the patient with scheduled sessions (8 group/individual sessions), and will use national reference medication for treatment of smoking, as well as the cognitive behavioral approach. If possible, the patient should be followed up to 1 year of treatment. The cognitive behavioral approach is a psychological approach that is based on working out the automatic thoughts that the smoker has and that lead him/her to get a cigarette. These thoughts are often accompanied by emotion and behaviors associated with smoking. It is important for the patient to feel welcomed by the doctor, to show empathy, not to judge or condemn because of difficulties in smoking cessation. Another aspect is that the better the smoker knows his/her addiction profile, the easier it is to work on ways to control smoking addiction.^259,260^

In the cognitive-behavioral approach it is necessary to: distinguish the patient’s automatic (dysfunctional) thoughts - example: “if I do not smoke I cannot think” - helping him to seek coping strategies for situations other than getting a cigarette. Behavioral techniques most commonly used: self-observation, control of stimuli or triggers that lead to smoking (telephone, computer, alcohol, bathroom, car), identification and learning of functional thinking patterns, relaxation techniques, deep breathing, postponement and breaking of conditioning, assertiveness training (so that one can face situations where there is the temptation to smoke), self-instruction (situations in which patients are taught to argue with themselves about the situation that tries to induce them to smoke) and problem-solving so that patients are taught about appropriate ways to solve a problem situation.^260-262^

Instruments that help in assessing and understanding the patient profile:

- ***Prochaska and Di Clemente Scale for Behavior Change:*** This scale provides a model that allows one to clearly and objectively evaluate which phase of behavior change the patient is in. Quitting smoking is a dynamic process that repeats over time and has different stages. At each stage, the individual uses different cognitive and behavioral processes.^263^ The authors propose five different stages in this process. Pre-contemplation is characterized by the absence of intention to change behavior. The individual does not perceive, in this case, the act of smoking as a problem. Contemplation implies some awareness of the problem. It is perceived, and there is an intention to change, but there is no notion of when, so there is no commitment. Preparation is the pre-action stage. There is a clear intention to change, the individual already has some initiatives regarding change, but the action is not yet effective. Action is already a behavior change to try to solve the problem. The individual spends time looking for treatments and promotes changes that must be sustainable. Maintenance is the stage at which such changes must be consolidated, encompassing all that has been achieved at the action stage. The problem is that these stages do not occur sequentially in the process of change, but rather in a spiral way; that is, the individual may be at an earlier stage, and at some point, for some reason, regresses to an earlier stage. and then evolves again. When he returns to an early stage of pre-contemplation, he may relapse back to his previous smoking pattern. The individual can start the whole process again, and be able to abstain once again. Basic signs that indicate that the smoker is ready for change: has less resistance, asks fewer questions about the problem (addiction), asks more questions about change (what and how to do it), takes a resolving attitude (feels decided), makes more self-motivational claims, talks about life after change (difficulties and benefits), begins to experience some changes (decreased smoking).
- ***Motivational Interviewing:*** It is a viable alternative in the treatment of dependent behaviors within brief interventions, as the initial impact seems to influence the motivation for behavior change. Motivational interviewing employs a particular way of assisting in recognizing present or potential problems as well as in behavioral change aimed at solving such problems. Motivational Interviewing Strategies: Providing Guidance, Removing Barriers/Assisting Obstacles, Providing Alternative Smoking Options, Decreasing Undesirable Aspects of Behavior, Practicing Empathy, Giving *Feedback*, Clarifying Objectives, and Actively Assisting and Taking Care of Relapse Prevention - Coping With Abstinence.^264^
- ***Fagerström Scale:*** This is an evaluation scale that allows us to determine the degree of physical dependence on nicotine. It should be used in the initial assessment of the smoker on arriving for treatment. If medication is needed, it helps to determine which medication is best and how much to take.^265,266^ In this case, it is noteworthy that the use of medication should not be considered only in cases where Fagerström is ≥ 5. It is known today that a very low Fargeström means that psychological dependence is very high and in this case the medication helps in reducing withdrawal symptoms.^260^
- ***Reason for smoking scale:*** This is a rating scale that allows us to check in which situations the smoker uses the cigarette. It has to do with physical, psychological dependence and conditioning and helps to clarify to smokers the risk situations of their daily life. This scale assesses: stimulation, ritual handling, pleasure in smoking, tension reduction/relaxation, physical dependence, habit/automatism, and social smoking. These items should be worked through throughout the smoker’s intensive approach process.^267^

### 7.4. Pharmacological Treatment of Smoking

#### 7.4.1. Secondary Intervention Smoking

The CV effects of smoking are harmful, so CVD is the leading cause of death among smokers.^268^ Smokers with CVD should stop smoking.^269^

The safety of first-line anti-smoking drugs such as varenicline, bupropion and nicotine replacement has been reiterated by clinical studies designed^270^ to answer publication questions that suggested there may be CV risk with the use of anti-smoking medication.^271^ The CATS study,^270^ among others, proved that there is no such risk. Thus, respecting the contraindications of each product, the use of these drugs should be encouraged so that the patient can really quit smoking, as the drugs increase cessation success rates.^272^

The prescription of anti-smoking drugs is essential for improving the effectiveness of smoking treatment, as well as conducting follow-up appointments and encouraging changes in patients’ habits and behavior.^273,274^

The main features of first-line anti-smoking drugs are:

***1. Nicotine Replacers (Chart 7.1)***

**Chart 7.1:** Initial evaluation in approach to smoking^300^

**Anamnesis**
• Scales: Fagerström (for nicotine dependence)^265^ - Table 7.2
• Prochaska and DiClementi (for motivation)^263^ - check the patient's counseling techniques - Table 7.3
• Clinical and/or psychiatric comorbidites (diabetes, hypertension, depression, alcoholism, stroke, convulsion, cancer)
• Continuous use medications
• Risk factors for CVD (dyslipidemia, uso of oral contraceptives or estrogen)
• Pregnancy or breastfeeding
• Questions about smoking:
- How long have you been smoking
- How many cigarettes do you smoke per day
- Have you tried to quit smoking and what was the result
- Are you interested (or thinking) about quitting smoking?
• Questions about smoking cessation:
- Are you considering a date to quit smoking and would you like help
- If you have tried to quit, if you have succeeded, if you have taken any medication and how long you have been without smoking
**Physical examination**
• Monitor BP, especially during bupropion use
• Monitor weight: weight gain can be a barrier to starting smoking cessation and a predictor of relapse
**Complementary examinations**
• Complete blood count, liver function tests, blood glucose, lipid profile and serum biochemistry
• Chest X-ray
• Electrocardiogram
• Spirometry (not always readily available)
• Measurement of COex, if possible. This parameter is directly related to carboxyhemoglobin and cigarettes smoked per day. The cutoff point is 6 ppm

COex: carbon monoxide; CVD: cardiovascular disease; BP: blood pressure.

**Table 7.2:** Fagerström test for nicotine dependence^265^

1. How long after waking up do you smoke the first cigarette?
[3] Within 5 minutes [2] Within 6-30 minutes [1] Within 31-60 minutes [0] After 60 minutes
2. Is is hard for you not to smoke in forbidden places?
[1] Yes [0] No
3. Which of the cigarettes that you smoke during the day give you the most satisfaction?
[1] The first in the morning [0] Others
4. How many cigarettes do you smoke per day?
[0] Less than 10 [1] 11-20 [2] 21-30 [3] More than 31
5. Do you smoke more often in the morning?
[1] Yes [0] No
6. Do you smoke even when sick, when bedridden most of the time?
[1] Yes [0] No

→Total: [0-2] very low; [3-4] low; [5] moderate; [6-7] high; [8-10] very high.

**Table 7.3:** Stages of motivation and counseling techniques^263^

• Precontemplative: not yet concerned; not ready for behavior change → briefly report risks of continuing to smoke and encourage patient to think ↓
• Contemplative: recognizes that you need and want to change, but still want to smoke (ambivalence) → ponder the pros and cons of cessation and remain available to talk ↓
• Determined: Wants to quit smoking and ready to take the necessary action → choose a date to quit smoking ↓
• Action: engage in attitudes intended to bring about change and abstain → follow-up to prevent relapse and relieve withdrawal symptoms ↓
• Maintenance: Keeps the behavior change achieved and remains abstinent → reinforce the benefits gained from quitting, identify risk situations for relapse and the skills to cope with them ↓
• Relapse: unable to maintain achieved abstinence and returns to smoker behavior → offer support, review and resume the whole process

Nicotine is primarily responsible for cigarette addiction and nicotine replacement therapies (NRT) have been used since 1984 to help smoking cessation. The forms of NRT currently used and available in Brazil are transdermal and oral (lozenges and chewing gums). Both are effective in smoking cessation and are often used in combination and can double the rate of smoking cessation compared with placebo.^268,275^

***2. Transdermal Nicotine***

Effectiveness - compared to placebo - RR = 1.9 (95% CI 1.7-2.2).

6-Month abstinence rate - RR= 23.4 (95% CI 21.3-25.8).

- Doses: 21 mg; 14 mg; 7 mg.
- Presentation: patches for transdermal application.
- Route(s) of administration: transdermal application with daily replacement.
- Dose schedule: use of each presentation for 4 weeks on average, with gradual dose reduction. e.g.: (21, then 14, then 7 mg/day).
- Care in administration: application to upper chest, anterior and posterior region, and upper lateral region of arm.
- Adverse reactions: itching and redness at the application site, nausea, feeling sick, tachycardia with overdose.
- Contraindications: Dermatological disorders that prevent the application of the patch, 15 days after episode of acute myocardial infarction (AMI), pregnancy and breastfeeding.
- Overdose (toxicity): nausea, feeling sick, tachycardia, hypertensive crisis.

***3. Nicotine for oral use - nicotine gum or lozenge***

Effectiveness - compared to placebo RR = 2.2 (95% CI 1.5-3.2).

6-Month abstinence rate - RR= 26.1 (95% CI 19.7-33.6).

- Doses: 2 and 4 mg.
- Presentation: chewing gum or lozenge.
- Route(s) of administration: oral.
- Dose schedule: use at times of craving, intense desire to smoke, instead of cigarettes (1 to 15 gums/day).
- Care in administration: Swallow with a glass of water before use to neutralize oral pH, which may be altered by food intake and food residue removal, which may decrease absorption by the oral mucosa.
- Adverse reactions: nicotine gum - temporomandibular joint pain when chewed quickly and incessantly; oropharyngeal irritation and nausea when chewed quickly and frequently.
- Adverse reactions: nicotine lozenge - oropharyngeal irritation and nausea when chewed rather than allowed to dissolve in the mouth or overuse.
- Contraindications:

*Nicotine gum -* Inability to chew, active peptic ulcer, 15 days after AMI.

*Nicotine lozenge -* active peptic ulcer, 15 days after AMI.

- Overdose (toxicity): nausea, feeling sick, tachyhardia, hypertensive crisis.

***4. Bupropion hydrochloride (Chart 7.2)***

**Chart 7.2:** Non-nicotine therapy^300^

**Bupropion hydrochloride**
• Simulates some of the effects of nicotine on the brain by blocking the neuronal uptake of dopamine and norepinephrine. It may be used in combination with nicotine replacement therapy with patch
• Excellent option for subgroups of relapse-prone smokers with depression after smoking cessation, and for women and those with a high degree of dependence. Success rates for smoking cessation range 30 to 36%
• Therapeutic scheme: Start treatment 8 days before smoking cessation
- 150 mg in the morning for three days, followed by 150 mg in the morning and afternoon at 8-hour intervals for 3 months, which may be extended for up to 6 months. Control blood pressure and, if elevated, the dose may be reduced to 150 mg/day before stopping in refractory cases. Reduce doses in renal and hepatic impairment to 150 mg/day. Monoamine oxidase inhibitors should be discontinued for up to 15 days before starting bupropion. Use with caution or avoid in patients on antipsychotics, theophylline and systemic steroids, as it favors the onset of seizures
• Contraindications:
- Absolute: history of convulssions (even febrile), epilepsy, brain injury, electroencephalogram abnormalities, brain tumor, severe alcoholism, anorexia nervosa and bulimia, pregnancy and breasfeeding
- Relatives: Combined use of barbiturate, benzodiazepines, cimetidine, pseudoehedrine, phenytoin, oral hyproglycemic agents or insulin
**Varenicline tartarate**
• α4β2 nicotinic acetylcholine receptor partial agonist, which mediates the release of dopamine in the brain
• Has double effect: reduces withdrawal symptoms and the desire to smoke
• Therapeutic schedule: start 1 week before the cessation date, with 0.5 mg for 3 days in the morning, followed by 0.5 mg from the 4th to 7th morning (7 h) and in the afternoon (19 h) and 1 mg/day for 3 months in the morning (7 h) and in the afternoon (19 h), which may be extended to 6 months in cases without complete cessation of smoking or risk of relapse. Varenicline is administered orally and does not undergo hepatic metabolism, and it isrenally excreted practically unchanged
• Adverse effects: nausea (20%), headache, vivid dreams and weight gain. Rarely, mood changes, agitation, restlessness, and aggressiveness
• Because it is not metabolized by the liver, varenicline does not interfere with concomitant use of digoxin, metformin or warfarin. Cimetidine may increase varenicline bioavailability
• It should be used with caution in patients with renal failure
• Contraindication: pregnancy, breastfeeding, less than 18 years old, bipolar disorder, schizophrenia or epilepsy

Bupropion is a dopamine and norepinephrine reuptake inhibitor that is effective in smoking cessation,^268,276^ decreasing nicotine withdrawal symptoms. Because it is an antidepressant, it can help control depressive symptoms that may arise during the smoking cessation process.

Effectiveness - placebo compared - RR = 2.0 (95% CI 1.8-2.2).

6-Month abstinence rate - RR = 24.2 (95% CI 22.2-26.4).

- Presentation: 150-mg prolonged-release lozenges.
- Route of administration: oral.
- Dose schedule: 1 tablet daily for 4 days, then increase to 1 tablet twice daily with a minimum interval of 8 hours.
- Care in administration: Avoid night administration to minimize the risk of insomnia.
- Adverse reactions: dry mouth, insomnia (interrupted sleep), constipation, epigastric pain, dizziness.
- Contraindications: Absolute: risk of seizure (history of seizure, epilepsy, childhood febrile seizure, known electroencephalogram abnormalities); alcoholism; use of monoamine oxidase inhibitors (MAOI) in the last 14 days; cerebrovascular disease; central nervous system tumor, head trauma.
- Warnings/precautions: The combination of bupropion with nicotine replacement, especially patches, may increase BP; for this reason, it should be evaluated at all doctor visits. Alcohol use may predispose to seizure, so patient should be advised to restrict alcohol consumption during use.
- Overdose (toxicity): convulsions.

***5. Varenicline tartrate (Chart 7.2)***

Varenicline^268,277^ it is a partial nicotinic receptor agonist in the central nervous system. The substance is the most effective medication among the first-line drugs in treating smoking.^278,279^

Effectiveness - compared to placebo - RR = 3.1 (95% CI 2.5-3.8).

6-Month abstinence rate - RR = 33.2 (95% CI 28.9-37.8).

- Doses: 0.5- and 1-mg varenicline tartrate tablets.
- Route of administration: Oral use.
- Dose schedule: Start with 0.5 mg once a day. On the 4^th^ day, prescribe 0.5 mg twice a day. On the 7^th^ day, prescribe 1 mg 2 times a day. Prescribe for 12 to 24 weeks. Varenicline therapy does not require immediate cessation of smoking. It is recommended to stop smoking from the 14^th^ day after starting the medication.
- Care in administration: take after meal with water (between 150 and 250 ml to reduce nausea).
- Adverse reactions: The most expected side effect with this substance is nausea (30% of patients). This effect is minimized by taking the medication after meals and with a glass of water. Less than 6% of patients discontinue medication because of this effect. Other effects reported to a lesser extent are insomnia (14%), headache (10%), constipation (6%), abnormal dreams (dream recall and actual content) and flatulence, which in some circumstances require dose reduction (1 mg/day), but rarely cause discontinuation of medication.
- Contraindications: Absolute - terminal renal failure, pregnancy and breastfeeding. Dose adjustment in patients with severe renal failure (see adjustment table).
- Precaution for use: Caution should be exercised when using in patients with a history of psychiatric illness such as severe depression, bipolar disorder and panic syndrome. Although the causal connection has not been demonstrated, and considering that smokers have a higher risk of depression and suicidal ideation,^280^ the US Food and Drug Administration (FDA) in 2009,^281^ warned about the possibility of mood swings, restlessness and suicidal ideation among users of varenicline, and is therefore not recommended in patients with non-stabilized psychiatric disorders.

In 2011, Singh et al.^271^ conducted a meta-analysis with some varenicline studies warning of possible risks of CV events among users. After careful analysis of the study, it was concluded that a significant number of patients who used varenicline in randomized trials were not included in the meta-analysis and did not present with any CV event.^282^ Prochaska and Hilton^283^ performed a more comprehensive meta-analysis, including all varenicline studies, and found no risk of increased CV event in the varenicline versus placebo group. The safety of varenicline was assessed by Rigotti et al.,^284^ when they analyzed, in a randomized, placebo-controlled manner, the efficacy and safety of varenicline in patients with CVD. The authors found no additional CV risk in the varenicline group.

- Overdose (toxicity): nausea, feeling sick, vomiting.

Second line medicine:

***1. Nortriptyline***

Nortriptyline is a tricyclic antidepressant that blocks the reuptake of norepinephrine into the central nervous system. It is a 2^nd^ line drug in the treatment of smoking. The FDA has not yet approved its use for treatment because, although its efficacy is similar to that obtained with NRT or bupropion, there is a greater risk of side effects from the medication.^268,281^ The recommended dose is 25 mg/day, as a single dose, gradually increasing to 75 to 100 mg per day. Use is not recommended in patients with structural heart disease of any nature because of the risk of inducing conduction disorders and arrhythmia.

### 7.5. Anti-Smoking Drug Combinations

The effectiveness of first-line anti-smoking drugs is between 20 and 25% for nicotine replacement and bupoprione, and does not exceed 35% with varenicline.^268^ Thus, we can imagine that out of 10 patients treated, about 3 will quit and 7 will not.

The combination of anti-smoking drugs seems to be a reasonable application option to improve success rates. Despite the increase in cost, it should be considered that quitting smoking has an substantial cost-benefit ratio, so the proposal is perfectly viable, leaving the perspective of dealing with the possible increase in side effects as the main factor to be managed.

Some studies with the combination of patches and oral nicotine have shown improved results. Meta-analysis of 9 studies^277^ that combined a nicotine patch with a nicotine rapid-release drug (gum, spray, lozenge) proved to be more effective than a single type of NRT (RR 1.34, 95% confidence interval 1.18 to 1.51).

The combination of NRT and bupropion was more effective than bupropion alone in the meta-analysis of 4 studies.^277^ (RR 1,24; 95% confidence interval 1.06 to1.45).

The combination of varenicline and bupropion appears to be the most effective of all (Evidence B);^285^ however, randomized studies^286^ of greater consistency need to be performed.

### 7.6. Future Proposals

The use of serotonin reuptake inhibitors has not proven to be an option for treatment of withdrawal symptoms,^281^ but considering how often depressive symptoms manifest during smoking cessation,^285^ with or without drugs,^286^ randomized trials to test concomitant use of this drug should be conducted to assess whether results are improving, as nicotine has an action on monoaminoxidase A, which is responsible for serotonin degradation, among many other neurotransmitters, which would explain the high frequency of this condition. smoking cessation, with or without anti-smoking medication. Bupropion and varenicline have no action on serotonin, explaining the higher frequency of mood disorders in drug users compared to those on nicotine replacement.

We believe that this event is more likely to occur in varenicline users due to the high antagonist potency in the a4 b2 receptor, thus preventing nicotinic action, even if the patient smokes. From this perspective, the longitudinal, observational study that evaluated the effectiveness of the combination of varenicline, bupropion and sertraline^287^ had a better success rate among those that used all three drugs. These findings warrant corroboration through a randomized, placebo-controlled study, so that there is indeed robust evidence of the benefit of these combinations, as well as testing whether the use of serotonin reuptake inhibitors is confirmed as an ancillary strategy in anti-smoking treatment in patients who manifest depressive symptoms during smoking treatment.^288,289^

Nicotine vaccines,^290^ long awaited to comprise the therapeutic arsenal, are still under study. They act by stimulating the immune system to produce specific antibodies that bind with great affinity to nicotine in plasma and extracellular fluids.Nicotine bound to antibodies cannot cross the blood-brain barrier because of its size, the vicious circle of gratification for brain receptor activation is broken. The main brands under study are: Nic-VAX®, TA-Nic® and Nic-Qb®.

### 7.7. Nicotine Electronic Devices (Electronic Cigarette, Heated Cigarette, Pen Drives)

These devices were launched in 2006 and have since been refined by their manufacturers to replace the conventional cigarette. The industry of these products insists on seeing them as “smoking cessation treatment,” arguing that smokers, by replacing the use of ordinary cigarettes with these devices, would reduce the risk of disease by consuming a product with less toxic substances. With this argument, they are investing heavily in marketing, and Phillips Morris, one of the largest conventional cigarette manufacturers in the world, is widely publicizing its strategy to stop producing ordinary cigarettes and replace it with heated cigarettes, also an electronic nicotine-release device, without combustion.^291^

The marketing, importation and advertising of any electronic smoking device, including electronic cigarette and heated cigarette, have been banned by Anvisa (National Health Surveillance Agency) since 2009 in Brazil (RDC 46). The agency considers that there is no scientific evidence to aid smoking cessation - meaning cessation as a treatment process for nicotine addiction - or scientific arguments that actually prove reduced morbidity and mortality by tobacco-related diseases in populations that have replaced tobacco use. Although they contain less toxic substances than conventional non-combustion cigarettes, those present are not harmless, and nicotine is a substance known to have CV effects, and perpetuates the addiction condition.^292^

The impact of the use of these products on people’s health is not yet known, and although manufacturers are betting on their use as a harm reduction policy, the concern is that there is an epidemic of consumption and a setback in encouraging smoking cessation worldwide. Therefore, WHO does not recognize these devices as a treatment for smoking and warns that they cause nicotine addiction just as much as regular cigarettes, and looks forward to studies evaluating the impact of these products on morbidity and mortality.^293^

### 7.8. Hookah

Contrary to popular belief that hookah is less harmful and less addictive than cigarettes, research shows that both carry significant health risks, and may induce nicotine addiction.^294,295^

The world panorama shows that the trends of hookah use are alarming and have shifted from being a social phenomenon among young people in some regions to becoming the beginning of a global epidemic.^296^

In Brazil, the frequency of hookah use in the Brazilian adult population aged 18 to 59 years was determined in a population-based cross-sectional study using the 2013 National Health Survey (PNS). Of the 60,225 adults interviewed, 15% reported using any tobacco use, with the frequency of hookah use being 1.2% (95% confidence interval 0.8 - 1.6), higher in the male, white and younger age groups, with medium to high schooling, and urban and southern and midwestern residents; Among those who tried hookah, 50% used it sporadically, 12.8% monthly, 27.3% weekly and 6.8% daily. These results point to the need for supervision and educational campaigns on the risks of hookah use.^297^

### 7.9. Conclusion

Pharmacological treatment of smoking should be considered as a secondary prevention strategy, mainly aimed at reducing CV injury. Smoking is a chronic degenerative disease, and should be viewed by the cardiologist like the other common illnesses in their care routine, such as hypertension and DM.

Defining criteria for choosing which anti-smoking drug will initially be used for patient treatment is still a challenge for treatment guides and guidelines because of the lack of systematization of models to be tested. In clinical practice, the choice of drugs is made on the basis of contraindications, drug availability and price, among other criteria. Therefore, systematically discussing criterion models for this choice becomes relevant and necessary for increasing the efficacy of anti-smoking treatment.

The high degree of nicotine dependence^298^ could be a factor in decision making, as well as factors that identify subpopulations that benefit from any particular drug, considering gender, age, pharmacogenetics^299^ (genetic polymorphism of nicotinic, dopamine and hepatic receptors) among others. These factors are not yet known at this time.

Recommendations for addressing adult smokers can be found in Table 7.1 and Charts 7.1, 7.2, 7.3 and 7.4.

**Table 7.1:** Recomendations for approach for adult smokers

Recomendation	Recommendation class	Level of evidence	Reference
Routine assessment of smoking for adults at all health professional appointments, recorded in medical records	I	A	^2,10,300^
Systematic counseling for all adults on smoking cessatio	I	A	^2,10,300^
A combination of behavioral and pharmacological interventions is recommended for all adults to minimize dropout rates	I	A	^2,10,300^
Smoking cessation is recommended for all adults to reduce cardiovascular risk	I	B	^2,10,300^
A multidisciplinary team should be allocated to facilitate smoking cessation in all health systems	IIa	B	^2,10,300^

**Chart 7.3:** Nicotine Replacement Therapy^300^

**Rapid Nicotine Replacement: chewing gum and lozenge**
• Used when craving (imperative need to smoke) or at 1- to 2-hour intervals
• Promotes faster release of nicotine. It can be combined with nicotine patch or combined with bupropion and varenicline
• The approximate time for nicotine release is 5 minutes with the lozenge and 10 minutes with the gum
• The maximum tolerated dose is around 10 gums or lozenges per day
• Patients should chew/suck the gum/lozenge until it tastes spicy. At this point, they should stop for 2 minutes (time to absorb nicotine) until the taste disappears and then chew/suck again by repeating the cycle within 20 minutes for a second nicotine release. They should drink a glass of water before use to neutralize oral pH, which changes with food intake, and to remove food residues, which may decrease absorption by the oral mucosa
• Side effects: hypersalivation, nausea, hiccups, gingival ulceration (which may lead to tooth softening) and temporomandibular joint (TMJ) pain
• Contraindication: inability to chew/suck, oral mucosa lesions, peptic ulcer, TMJ subluxation and use of mobile dental prostheses
**Slow Replenishment: Nicotine Patch**
• The patches are provided in boxes of seven units each, in doses ranging from 7 to 25 mg
• They are indicated to maintain a continuous level of circulating nicotine for 24 hours, in a process of gradual smoking cessation
• They may be indicated as pre-cessation therapy for 2 to 4 weeks in smokers who have a hard time reducing the number of cigarettes and setting a date to quit
• The patches should be applied in the morning to covered areas on the upper chest or anterior, posterior and upper lateral regions of the arm, rotating between these sites and changing at the same time of day. Sun exposure should be avoided at site
• They may be used in combination with bupropion or varenicline
• Therapeutic scheme:
- Smoker of 20 cigarettes/day and/or with Fagerström score of 8-10 points: A patch of 21 to 25 mg/day from the 1st to 4th week; 14 to 15 mg/day from 5th to 8th week; 7 mg/day from 9th to 10th week. It is suggested to put the patch on in the morning just after waking up. In cases of insomnia, it should be removed after 16 hours of use. In special cases of high dependence and in the absence of contraindication, up to two 21-mg patches may be used
- Smoker of 10-20 cigarettes/day and/or with Fagerström score of 8-10 points: A patch of 14-15 mg/day in the first 4 weeks followed by 7 mg/day from the 5th to 8th week
• Side effects: pruritis, rash, erythema, headache, nausea dyspepsia, myalgia and tachycardia with overdose
• Contraindications: history of recent myocardial infarction (in last 15 days), severe cardiac arrhythmias, unstable angina pectoris, peripheral vascular disease, peptic ulcer, skin diseases, pregnancy and breastfeeding

**Chart 7.4:** Standard pharmacological treatment for smoking^300^

Medication	Start of treatment	Therapeutic scheme	Duration (weeks)
Nicotine replacement therapy: patch	On date chosen for smoking cessation	21-25 mg/day - 4 weeks 14-15 mg/day - 4 weeks 7 mg/day - 2 weeks Smokers with greater dependence may need doses higher than 21 mg	8 to 10
Nicotine replacement therapy: gum or lozenge	On date chosen for smoking cessation	2 or 4 mg: 1 to 4 times a day	8 to 10
Non-nicotine therapy: bupropion	One week before date chosen for smoking cessation	First to third day - 150 mg, 1 x day Fourth to last day- 150 mg, 2 x a day	12
Non-nicotine therapy: varenicline	One week before date chosen for smoking cessation	First to third day - 0.5 mg, 1 x day Fourth to seventh day - 0.5 mg every 12 hours Eighth to last day - 1 mg every 12 hours	12

## 8. Physical Activity, Physical Exercise and Sport

### 8.1. Introduction

Physical inactivity is one of the major public health problems, and physical inactivity, which is strongly related to all-cause and CVD mortality, is highly prevalent in Brazil and worldwide.^301,302^ Increased physical activity is related to health gain, better quality of life and greater life expectancy.^303-307^ Therefore, both in an individual and population-based CVD prevention strategy, it is of utmost importance to prioritize a strong fight against sedentarism, and requiring the questioning of PA habits and encouragement of adopting a more active lifestyle should be routinely done at medical office visits.^308^

### 8.2. Relevant Concepts and Expressions in Physical Activity

PA is used as a broad term that includes both structured and unstructured forms of leisure, sports, transportation, and domestic and work-related activities. physical activity involves body movement, with increased energy expenditure in relation to rest, and can be classified in terms of intensity as mild, moderate or high. Physical exercise is defined as a subset of structured activities aimed at improving cardiorespiratory fitness, balance, flexibility, strength and/or power and even cognitive function, particularly important in the elderly.^309^

Thus, physical activity, physical exercise and sports are related but distinct terms, and Table 8.1 defines some concepts and expressions.

**Table 8.1:** Main concepts and terms in the subject: exercise, sedentarism and health

Concepts and terms	Significance
Physical aptitude	Ability to perform activities and physical exercises expected for their age group, gender and body, which promote health, survival and adequate functionality in the environment in which they live. It is divided into aerobic and non-aerobic components (muscle strength/power, flexibility, balance and body composition).
Physical activity	Any body movement produced by skeletal muscles that results in energy expenditure.
Physical exercise	Structured and repetitive physical activity, for the purpose of maintaining or optimizing physical fitness, body aesthetics and health.
Sport	Physical exercises with variable energy demand, involving rules and competitions and aimed at individual or collective winning.
Sedentarism	It is a condition in which there is no regular exercise or frequent physical activity involving energy expenditure > 2 to 3 times that at rest, at work, personal transportation or leisure.
Exerciser	One who works out regularly.
Athlete	One who simultaneously meets the following criteria: a) training in sports to improve performance, b) actively participating in sports competitions, c) being formally registered in sports organizations, and d) having sports training and competition as their focus of interest or way of life.

There is a strong association of different levels of physical fitness components with all-cause mortality and the occurrence of unfavorable CV events, with inverse association, i.e., the lower the physical fitness, the higher the mortality,^310-317^ requiring preventive action, focusing on combating physical inactivity as of childhood. WHO recently presented specific recommendations for children 0 to 5 years of age related to daily physical activity/exercise and sleep times, which considerably limits or restricts sitting time in front of screens.^318^ Table 8.2 presents a classification of the profile of children and adolescents according to physical exercise.^319^

**Table 8.2:** Childhood and adolescence profile according to physical exercise (adapted from Balassiano et al.)^319^

Score	Definition	Childhood/Adolescence
0	Sedentary or very inactive	Sometimes riding a bicycle, often dismissed from physical education at school.
1	Little active	Normal frequency in school physical education and short and intermittent periods of sports or dancing.
2	Moderately active	Regular participation most of the time in physical activity classes or sports activities or dance or fighting academies.
3	Very active	Regular and frequent participation in various sports activities on most days of the week.
4	Very active and competitive	Participation most of the time in training and/or sports competition or regular and frequent practice of predominantly aerobic exercise.

### 8.3. Main Acute and Chronic Effects of Exercise

The effects of exercise can be divided into acute and chronic.^320^ The acute effect is that which dissipates rapidly and may be immediate after a single session or last for up to 24 hours (subacute or late acute effect). Improvement in flow-mediated response with respect to endothelial function is an example of the acute effect of a single exercise session. The chronic effect is achieved by repeated acute/subacute effects and can be evaluated at rest, even if long after the last exercise session. Resting bradycardia observed in athletes of predominantly aerobic modalities is an example of chronic effect. Repetition of responses can produce a chronic effect, as in the case of decreased blood pressure. Some of the main effects of exercise are listed in Chart 8.1.

**Chart 8.1:** Main acute and chronic effects of exercise. NO: nítric oxide; VO_2_: oxygen consumption.

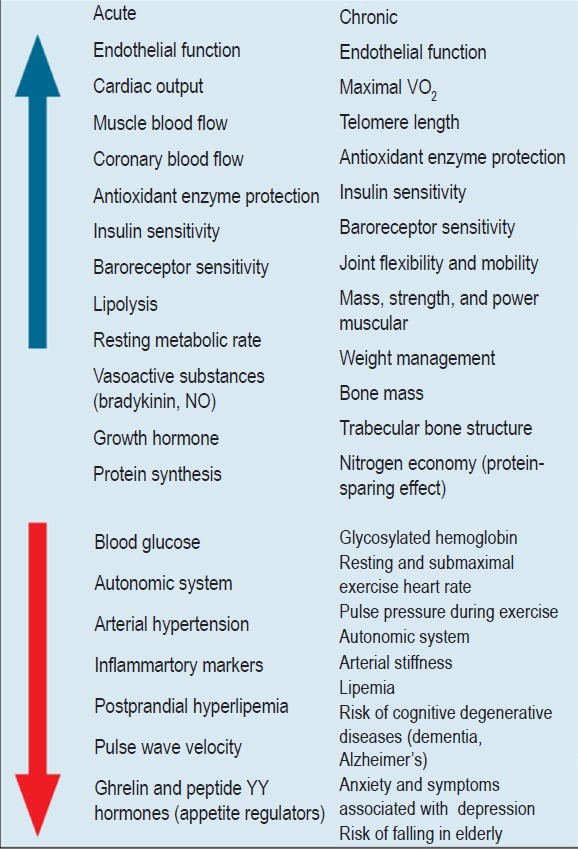

### 8.4. Epidemiological Rationale of the Benefits of Physical Exercise

In addition to aerobic fitness,^312-315,321,322^ other components of physical fitness are associated with prognosis, with higher mortality associated with poor fitness. proven as a predictor of mortality in middle-aged and elderly men and women.^311,323^ Other studies on muscle strength and power have also shown associations with mortality.^316,317^

Scientific findings support the previous recommendations of national^324,328^ and international guidelines^329^ that recommend the regular and combined practice of aerobic and resistance exercises. Flexibility and balance exercises should be part of an exercise program, especially aimed at the elderly.

Regarding PE, the greatest benefit is when comparing sedentary individuals and those who engage in very little or no exercise, since the positive impact of abandoning a sedentary lifestyle is very significant. However, comparing the varying degrees of aerobic fitness on an increasing scale, we realize that there is a continuous decrease in the risk of cardiac death and all-cause death. The higher aerobic fitness, the lower the risk is of total morbidity and mortality and CVD both in healthy individuals and in CVD patients.^312-315,321,322^

Greater physical fitness and amount of physical activity are associated with lower risk of developing hypertension.^330^ In already hypertensive individuals, PE reduces BP, and better results have been found with aerobic exercise (mean SBP reduction of 8.3 mmHg and DBP 5.2 mmHg). Smaller but significant reductions also occur with dynamic resistance training.^150^ Another useful and clinically safe strategy for BP reduction is based on manual isometric training.^150,331^ In patients with resistant hypertension - those with over-target BP despite the use of three or more antihypertensive medications, exercise in warm water (30 to 32°C) resulted in a more marked reduction in BP, and should be considered when available.^332-334^

The effects of reducing blood pressure levels during exercise occur immediately after the end of exercise and last up to 24 to 48 hours. Thus, as with drugs, this action in the CV system needs to be repeated periodically for the benefit to be chronically maintained. Regular PE exerts a hypotensive action, which adds to the effects of pharmacotherapy^335^ and may, in some cases, require reduction of medication doses.

It has also been suggested that dyslipidemic individuals with higher cardiorespiratory fitness, even without statin use, have a lower CVD risk than those with poor fitness using medication. Those with higher aerobic fitness and statin use have lower all-cause mortality, which reinforces the importance of physical exercise and greater physical fitness, even in patients with optimized drug treatment.^336,337^

### 8.5. Risks of Physical Activity, Physical Exercise and Sport

Healthy individuals have an extremely low risk of events due to regular exercise. A study of more than 20,000 physicians with an average follow-up of 12 years found that the risk was approximately one out of every 1.5 million hours of exercise exposure (during and within the first 30 minutes post-exercise).^338^ Thus, the recommendation to be physically active is quite safe and the fear of exercise-related problems should not be a barrier or justification for maintaining a sedentary lifestyle. This message needs to be widely disseminated to the population because the percentage of physically active individuals is very low in our country.^339^

For further information regarding sport and pre-participation assessment, it is recommended to read the recent update of the Brazilian Society of Cardiology Guidelines for Cardiology of Sport and Exercise.^328^

### 8.6. Recommendations for Exercise and Physical Activity

Although a meta-analysis has shown that simply stimulating the adoption of a more active lifestyle can increase physical activity levels,^340,341^ the physician’s guidance should be for physical exercise to be in an organized and structured manner. A good weekly goal for CVD health promotion and prevention is physical activity/exercise/sport for at least 150 minutes of moderate intensity or 75 minutes of high intensity.^10,342,347^ Getting more than 300 minutes per week of moderate to high intensity exercise may provide additional benefit. However, there is no scientific evidence for a clear delineation of an upper limit from which there would be a greater possibility of harm to a healthy individual.^303^

More recent studies have associated sedentary time, such as watching television, with higher all-cause mortality, CV mortality, and the risk of developing DM.^348^

### 8.7. Prescription for Exercises

Exercises may be prescribed for their characteristics, such as type (aerobic, muscle endurance, flexibility), modality (walking, running, cycling, dancing), duration (running time), weekly frequency and its intensity (Tables 8.4 and 8.5).

**Table 8.4:** Classification of physical exercise

Denomination	Characteristic
**By the predominant metabolic pathway**	
Alactic anaerobic	High intensity and very short duration
Lactic Anaerobic	High intensity and short duration
Aerobic	Low or medium intensity and long duration
**By pace**	
Fixed or constant	No change in pace over time
Variable or intermittent	With change in pace over time
By relative intensity *	
Low or light	Quiet breathing, very little panting. (Borg < 4)
Medium or moderade	Rapid breathing, panting but controlled. Can say a sentence. (Borg 4 to 7)
High or heavy	Very rapid breathing, a lot of panting. Speech difficult. (Borg > 7)
**By muscle mechanics**	
Static	No movement occurs and mechanical work is zero
Dynamic	There is movement and positive or negatice work

* For exercises with implements or weights that use localized muscle groups, the relative intensity can be expressed according to the maximum load possible to perform a maximum repetition (MR). For example, light intensity - up to 30% of 1 MR; medium intensity - 30 to 60 or 70% of 1 MR. Another alternative is to use Borg's psychophysiological scales. For the above classification, the scale version ranging from 0 to 10 was used.

**Table 8.5:** Prescribed methods for moderate intensity aerobic exercise

Method	Description
Subjective Sensation of Exertion (Borg)	Exercises with self-perceived exertion as moderate, medium or heavy, ranging 2 to 4 on the Borg scale 0-10 and 10 to 13 on the 6-20 scale
Speech Test	Performing intense exercises with heavy breathing but controlled so that a complete sentence can be said without pauses
HR Peak Percentages	Exercises at intensity between 70 and 90% of peak HR* Target HR = peak HR* x percentage
Reserve HR (Karvonen)	Exercises at intensity between 50 and 80% of reserve HR (peak HR* - resting HR). Target HR = resting HR + (peak HR* - resting HR) x percentage
Cardiopulmonary Test Thresholds	Performing exercises at iintensity between ventilatory thresholds 1 and 2 (anaerobic threshold and respiratory compensation point)

* Peak HR obtained in a maximum exercise test is preferred, since there are individual variations that cause errors in the prediction of HR by age, especially in patients using medications with negative chronotropic effects.

Previously sedentary patients may begin exercise at the lower end of the prescription and progress to higher intensities gradually over the following weeks. The progression should initially be made in the duration of the session and later in the intensity of the exercises. Already physically active patients, according to individual assessment, can perform exercises at more intense levels, aiming at a minimum of 75 minutes, ideally divided into two or more weekly sessions.

Localized muscular endurance and strengthening or power exercises have been shown to be very beneficial for general health and for CV and musculoskeletal systems, being of fundamental importance in patients with sarcopenia and/or osteopenia. They should be performed at least twice a week, favoring large muscle groups of the upper and lower limbs and trunk. They can be made using one’s own body or using attachments such as free weights, shin guards, elastic bands and weight machines. The load or weight for each exercise or movement must be individually adjusted. Due attention should be given to the execution of the movements so that technique and posture are correct.

There are different protocols for resistance exercise, from the number of exercises used per session from 6 to 15 (when done daily, there is a tendency to work a muscle group on alternate days), ranging from one to three sets for each exercise and also in the number of repetitions from 6 to 15. When training muscle power, the speed of execution should be as fast as possible in the concentric phase of the movement. In this case, only 6 to 8 repetitions per exercise are used, requiring only 20 to 30 second interval between each series to allow replenishment of the adenosine triphosphate (ATP) and phosphocreatine stocks needed to perform the next series. This strategy also has the advantage of greatly reducing the time dedicated to resistance exercises, which in many situations may represent the difference between adhering to the prescribed exercise program and not.

Flexibility exercises can offer osteomyoarticular benefits, health-related quality of life and prevention of falling in the elderly. By contributing to easier and more efficient joint movement, they ultimately reduce the demand for oxygen in moving situations and thus benefit the CV system. In these exercises, we seek to reach the maximum range of motion, reaching the point of slight discomfort and statically held position for 10 to 30 seconds. Flexibility exercises should ideally be individualized from specific assessments, such as the.^349^ In general, women tend to be more flexible than men, and there is a tendency for a progressive loss of flexibility with aging. tends to be proportionally larger in shoulder and trunk movements.

Depending on the age range, clinical conditions and objectives of the exercise program for a given patient, other forms of exercise may be included in the prescription, such as motor coordination and balance exercises, not to mention the numerous opportunities generated by more playful forms and exercise socializers such as ballroom dancing and Tai-Chi-Chuan.^351,352^

Performing assessments of aerobic and non-aerobic fitness allows a more individualized prescription of physical exercise, aiming to obtain the best results and, through risk stratification and search for hidden abnormalities, minimize the risks of exercise of any kind or intensity.

The initial evaluation consists of anamnesis, physical examination and ECG. More detailed assessments should be individualized, with exercise testing or maximal exercise cardiopulmonary testing, anthropometric assessment, muscle strength/power and flexibility. In the initial evaluation, we can quantify the functional deficit against the desirable, as well as set goals to be achieved. It is important to emphasize that even those with low initial levels of physical fitness can benefit from and become adherent to a supervised exercise program.^353^ It is also possible to obtain clinical and functional support for appropriate counseling on sexual activity based on the KiTOMI model proposed by Brazilian authors in 2016.^354^ It is essential for commitment to be encouraged in the patient on reevaluation, while measuring his/her evolution and benefits obtained.

### 8.8. Formal and Informal Physical Activity: Encouraging Referral, Implementation and Adherence

Although health benefits occur with relatively low intensity activities resulting from informal daily actions such as walking, climbing stairs, cycling and dancing, it is ideal that regular exercise (formal activities) also occur, which provides greater gains.

Patients with heart disease also benefit from regular physical exercise, ideally in the context of a formal CV rehabilitation program (or supervised physical exercise). CV rehabilitation acts on major disease outcomes, with proven effects by meta-analyses of randomized trials, reducing CV mortality, reducing hospitalizations^355,356^ and improving quality of life. In addition, CV rehabilitation is a cost-effective treatment.^357,358^

A possible way to improve exercise counseling by health professionals would be to combat their physical inactivity, as it has been shown that physically active people have greater knowledge about recommendations on prescribed exercises and can motivate more.^359^

In addition to direct medical practice, there is a need for changes in public and private policies, with the need for comprehensive strategies established through simultaneous actions, such as increasing physical activity in school programs; transport policies and systems that favor commuting through walking, cycling and public transportation; public education, including public awareness campaigns; sports organization at various levels (school, work, community etc.), with proposals that encourage and enable lifelong sports, from childhood to old age.

### 8.9. Final Messages

Physical inactivity should be combated by increasing physical activity in its various forms, both structured, physical and unstructured, favoring urban mobility with bicycle paths and facilitating travel through walking.

There is a consensus that a good and plausible weekly goal for health promotion and CVD prevention is to engage in physical activity/exercise/sport for at least 150 minutes of moderate intensity or 75 minutes of high intensity.

Given the current stage of knowledge, it can be said that:

- any amount of physical activity is better than none, and sedentarism is a worse possible situation;
- the benefits of exercise seem to be greater with more exercise, up to 5 times the minimum recommendation;
- there is no consistent scientific evidence that more than 10 times the minimum recommended exercise is harmful to health;
- there are no longitudinal studies relating heart disease to severe physical exercise, when performed regularly in healthy individuals.^303,360^

## 9. Spirituality and Psychosocial Factors in Cardiovascular Medicine

### 9.1. Concepts, Definitions and Rationale

#### 9.1.1. Introduction

There are lines of evidence that demonstrate a strong relationship between spirituality, religion and religiosity and the processes of health, illness and healing, composing together the physical, psychological and social aspects of the integral vision of the human being. In contrast to the easy conceptual assimilation, obstacles are observed, mainly due to lack of knowledge of the concept and scientific outdating, regarding the operationalization of the spirituality construct and the understanding of how to measure and evaluate its influence on health outcomes.^361^

Spirituality and religiosity are valuable resources used by patients to cope with illness and suffering. The process of understanding relevance, identifying demands, and providing adequate spiritual and religious support benefits both patients, the multidisciplinary team, and the health system itself. About 80% of the world’s population have some religious affiliation, and faith has been identified as a powerful mobilizing force in the lives of individuals and communities.^362,363^

#### 9.1.2. Concepts and Definitions

Definitions of spirituality typically merge with other constructs such as religiosity and the dimensions of psychological well-being, especially positive relationships with other people, purpose in life, and sometimes paranormal beliefs. Conceptual heterogeneity has been widely recognized and, for some authors, spirituality has no clear definition, the term being used inaccurately and inconsistently, varying according to religion, culture and time and therefore difficult to gauge.^364^

The meaning of the word religion has Latin derivations that refer to rereading (from scripture), to (re) binding or even to reelection (back to a god), inferring connections to deity, other people, or their beliefs and values. Although the term religion in the past (and in current theological erudition) has been used to grasp the institutional and individual dimensions of experience, contemporary references to religion increasingly imply institutional, social, doctrinal, and denominational characteristics of lived experiences.^361^

According to Koenig, religion is “an organized system of beliefs, practices and symbols designed to facilitate closeness with the transcendent or the Divine and foster understanding of one’s relationship and responsibilities with others living in the community”.^361,363-365^ Religion is a multidimensional construct that includes beliefs, behaviors, dogmas, rituals and ceremonies that can be practiced in private or public contexts, but are somehow derived from established traditions that have developed over time within a community. Religion is also designed to facilitate closeness with the transcendent and promote an understanding of one’s relationship and responsibility to others when living in a community.^365^ Religiosity is how much an individual believes, follows and practices a religion. It can be organizational (church, temple, or religious services) or non-organizational such as praying, reading books, or attending religious programs.

Historically, spirituality was considered a process that unfolded within a religious context, with institutions designed to facilitate the spiritualization of the practitioner. Only recently has spirituality been separated from religion as a distinct construct, in part because of the distancing from the authority of religious institutions in modern social life and the increasing emphasis of individualism on Western cultures.^361^

More recently, faced with the need to standardize a definition for spirituality in palliative care, a group of interprofessional specialists in palliative and spiritual care has defined spirituality as “a dynamic and intrinsic aspect of humanity, through which people seek meaning, purpose and transcendence and experience relationship with self, family, others, community, society, nature and the meaningful or sacred. Spirituality is expressed through beliefs, values, traditions and practices.”^366^

According to the Study Group on Spirituality and Cardiovascular Medicine (GEMCA) of the Brazilian Society of Cardiology, “spirituality is a set of moral, mental and emotional values ​​that guide thoughts, behaviors and attitudes in the circumstances of intra- and interpersonal relationship life.” One can also add the aspect of being motivated or not by the will and be subject to observation and measurement (http://departamentos.cardiol.br/gemca). We consider it important that spirituality be valued measurable in all individuals, regardless of religious affiliation, including atheists, agnostics, or even those with religious affiliation but without observing and practicing it. For some, both atheists and agnostics, while not believing or uncertain about God’s existence, still have a form of spirituality based on existential philosophy, finding meaning, purpose, and fulfillment in their own lives. Spirituality evokes concerns, compassion, and a sense of connection with something greater than us.^367^

Thus, spirituality may include religion and other universal views, but it encompasses much more general ways in which these experiences are expressed, including through the arts, relationships with nature and others, and for some through the concept of “secular humanism.” This emphasizing reason, scientific inquiry, individual freedom and responsibility, human values, compassion and the needs for tolerance and cooperation.

#### 9.1.3. Rationale and Mechanisms

A significant and growing body of evidence demonstrates an association between spirituality and religiosity and mortality indices, quality of life, with supposed mechanisms based on a huge range of biological and mediating variables, varying according to the model of healthy populations (or not). -healthy), forms of expression of spirituality and religiosity, research development scenario etc.^365,368,369^

In an American cohort predominantly composed of Christians > 40 years of age and followed for an average of 8.5 years, a lower risk of death was observed, regardless of confounding factors among those who reported religious services at least once a week compared to no presence. The association was substantially mediated by health behaviors and other risk factors.^370^

In a 2009 systematic review, spirituality/religiosity was associated with reduced mortality in studies involving healthy populations, but not in trials of the sick population. The protective effect of spirituality and religiosity was independent of behavioral factors such as smoking, alcohol, exercise, socioeconomic status, negative affect, and social support. When compared to organizational but not non-organizational religious activities, it was associated with longer survival.^371^

In the Women’s Health Initiative study involving more than 43,000 menopausal women, CV risk was highest in patients with private spiritual activity such as prayer, Bible reading, and meditation. Subgroup analysis suggests that this association may be determined by the presence of severe chronic diseases.^372^

It is possible that spirituality and religiosity will have little impact on outcomes once disease is established, identified and treated, and is more important in promoting resistance to health problems before they reach an advanced stage. It should also be noted that religious coping is often used but may have positive or negative connotations. Negative religious coping (such as passive acceptance of fatality and requests for direct intercession) can be detrimental in contrast to other beneficial effects.^371^

More recently, new cohort studies have made important contributions from the perspective of epidemiology and the associations between religious service, mortality and quality of life. In the Nurses’ Health Study cohort of over 74,000 nurses followed for up to 8 years, both all-cause mortality and CVD or cancer mortality were reduced by about 30% in women who attended religious services at least once a week compared to those without any participation.^373^ In this same population, attendance at religious services was significantly associated with lower suicide rate.^374^

Similarly, the follow-up of a large cohort of black American women showed a significant 46% reduction in mortality rate, comparing attendance at religious services several times a week with no frequency. On the other hand, involvement in prayer several times a day, religious confrontation or self-identification as a very religious/spiritual person did not correlate with mortality.^375^

This interface between spirituality and religiosity and health and illness processes is multifactorial and can, in part, be attributed to a behavioral self-regulation determined by religious affiliation and participation, with reduced consumption of alcohol, tobacco and drugs, reduction in the number of partners, better transport, food and access to health care. Emotionally, religious communion brings better positive psychology and social support, and positive spiritual coping can provide more hope, forgiveness, comfort, love, and other benefits.

In addition to behavioral aspects, most studies demonstrate the beneficial relationship between spirituality and religiosity and the physiological and pathophysiological variables of many clinical entities, including CVD. Despite the great heterogeneity between studies, better BP levels, neurohormones and autonomic nervous system activation, HR variability, dyslipidemia, CV risk, atherosclerotic disease, DM, CRP and other markers of inflammation and immunity have been observed.^365,368^ Another way of understanding the scope spirituality and religiosity may have on clinically relevant outcomes, including greater longevity, is expressed in the direct relationship with telomere size in leukocytes.^376,377^

### 9.2. Spiritual History and Scales for Measuring Religiosity and Spirituality

The degree of spirituality and religiosity of patients can be assessed in spiritual history or anamnesis, understood as “the set of questions asked to the patient to share their spiritual and religious values, to identify possible spiritual issues that may contribute to or undermine the therapy, as well as feelings that are used in daily life, in the life of relationship, whether positive (edifying) or negative (not edifying).” It should always be patient-centered and guided by what it expresses about its spirituality.^378^ At first, spiritual anamnesis as an integral part of clinical history should be obtained from all patients seeking medical attention, but especially those hospitalized with serious, chronic, progressive or debilitating illness.

#### 9.2.1. Why Address Spirituality and Religiosity

The approach to the subject is very important because many patients are religious or spiritual, and their beliefs influence how to cope with adverse situations in life and may help to cope with the disease. During periods of hospitalization or chronic illness, they are often removed from their communities and prevented from practicing their religious beliefs. In addition, personal beliefs may affect health-related decisions that may be conflicting with treatment.^379,380^

Many health professionals do not know if patients wish, agree with, or are open to this approach. Studies show that most patients would like their doctors to ask about spirituality and religiosity, generating more empathy and trust in the doctor and thus rescuing the doctor-patient relationship, with a more humane care.^381,382^

#### 9.2.2. Objectives of Spirituality and Religiosity Assessment

It is essential to seek to understand the patients’ beliefs, identify aspects that interfere with health care, evaluate the individual, family or social spiritual strength that will allow them to cope with the disease, offer empathy and support, help them to find acceptance of the disease and identify situations of conflict or spiritual suffering that will require evaluation by a skilled professional.^383,384^ In this evaluation, it is essential to detect negative feelings that may contribute to the illness or aggravation of the condition, such as hurt, resentment, unforgiveness, ingratitude, among others.

#### 9.2.3. How to Address Patient Spirituality and Religiosity

There are several ways to approach this issue, and most importantly it should be done sensitively without promoting religion or prescribing prayers or religious practices. Nor should the individual be coerced into adopting specific beliefs or practices.

Most of the time, the approach can be taken naturally, during the interview, as the doctor assesses psychosocial aspects.^365^ Patients should be asked about the importance of spirituality, religiosity, and religion to them, if this helps to cope with illness, generates stress or negative feelings (guilt, punishment etc.), or influences treatment adherence or decisions, and if there are any unmet spiritual needs.

The health professional should be sensitive and welcoming to religious beliefs and practices. If there are negative feelings, conflicts, or spiritual needs, the provider should solicit the participation of a trained individual or member of the patient’s community to properly address these issues. In the case of nonreligious patients or those who refuse to talk about the subject, the doctor may inquire about the ways in which individuals live with the disease, what promotes purpose and meaning for their life (family, friends, hobby etc.) and what beliefs may have an impact on their treatment.

For this approach to be non-conflictive, preparation and acceptance by both health professionals and patients is required.

#### 9.2.4. Scales and Instruments for Evaluating Spirituality and Religiosity

Measuring spirituality and religiosity in clinical practice and research is challenging, given the complexity of the elements and definitions involved in denomination, beliefs, religious/spiritual practices, participation in religious communities, support in dealing with illness, forgiveness, gratitude, altruism, spiritual well-being, pain or suffering and others.

The various psychometric instruments can be divided into tools for spiritual tracking or for spiritual history collection.^386,387^

***1. Spiritual Tracking -*** Evaluate the presence of spiritual needs that indicate deeper assessment. They are brief and easy to apply. Some of the instruments for spiritual tracking are listed in Chart 9.1.

**Chart 9.1:** Instruments spiritual tracking

Tracking tools	Spiritual domains evaluated
"Rush" protocol for tracking religiosity/spirituality^388^	Importance of religiosity/spirituality in dealing with disease. Spiritual strength or comfort
"Are you at peace?"^389^	Inner peace
"Do you feel spiritual pain or suffering?"^390^	Spiritual pain/suffering
Spiritual injury scale^391^	Guilt, anger, sadness, feeling of injustice, fear of death

Spiritual tracking provides important information and may indicate the need for further evaluation, although aspects remain to be studied (better time to apply in different stages of the disease and differences in cultural context, among others).

***2. Collection of spiritual history -*** They allow a broader evaluation of the different domains of patients’ spirituality and religiosity that may affect clinical evolution, their attitude towards CVD, self-care and their physical, mental and spiritual well-being during the disease.

They are well-structured instruments, addressing the different domains, but they should be applied informally from memory throughout the conversation with the patient, which serve as a tool or guide and should not be viewed rigidly, but as continuous learning and consequent familiarization with the task of completing the anamnesis. There are several validated instruments for collecting spiritual history, whether to evaluate spirituality and religiosity more broadly or for research purposes.

*2. a. Religiosity scales -* The religiosity index DUREL (Duke University Religion Index) is a scale of five items that measure three dimensions of religious involvement:

(1) assesses organizational religiosity (OR);
(2) assesses non-organizational religiosity (NOR); and
(3, 4 and 5) consider the assessment of intrinsic religiosity (IR) (Chart 9.2).

**Chart 9.2:** Duke University Religion Index (DUREL).

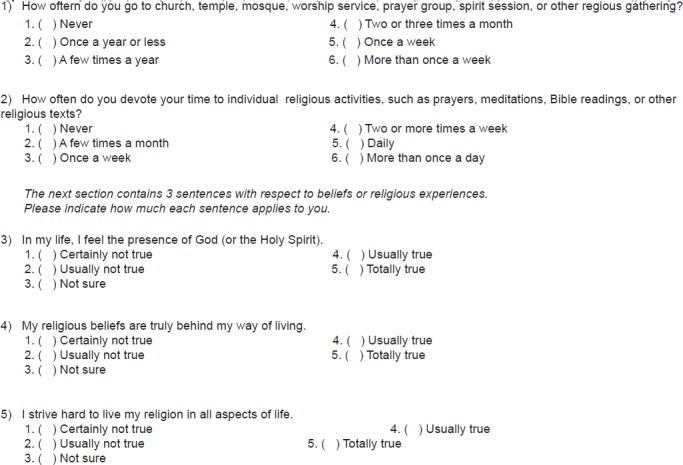

Validated in Brazil,^392^ DUREL is succinct and easy to apply, addresses the main domains of religiosity and has been used in various cultures. It has shown good psychometric characteristics, face and competitor validity, and good test-retest reliability, but does not evaluate spirituality. The dimensions of religiosity measured by DUREL have been related to several indicators of social support and health.^39^

*2. b. Assessment of spiritual history -* The assessment of spirituality involves a set of questions about its different domains that are associated with health outcomes, based on previously validated scales. Known by acronyms, some of the main instruments are FICA,^393^ HOPE,^394^ FAITH^380^ and SPIRIT.^395^

Among these, the FICA questionnaire has shown the best psychometric characteristics (Chart 9.3). It was created by doctors based on clinical experience and can be used in different clinical situations. It analyzes four dimensions (Faith or Belief, Importance and Influence, Community and Action in treatment), has easy application, fast execution and good memorization^383^ Similarly, HOPE has shown good performance in spiritual assessment (Chart 9.3).

**Chart 9.3:** FICA and HOPE questionnaires for spiritual history

FICA questionnaire	HOPE questionnaire
F - Faith/beliefs Do you consider yourself religious or spiritual? Do you have beliefs that help you deal with problems? If not, what gives life meaning?	H - Are there sources of hope? What are your sources of hope, comfort and peace? What do you cling to in hard times? What gives you support and makes you move forward?
I - Importance/influence What importance do you give to faith and religious beliefs in your life? Has faith or beliefs helped you cope with stress or health problems? Do you have any beliefs that may affect medical decisions or your treatment?	O - Religious organization Do you consider yourself part of an organized religion? Is this important? Are you part of a community? Does it help? In what ways does your religion help you? Are you part of a religious community?
C - Community Are you part of any religious or spiritual community? Does it support you? How? Are there any groups of people you really love or are important to you? Is there any community (church, temple, support group) that supports you?	P - Personal spiritual practices Do you have any spiritual beliefs that are independent of your organized religion? Do you believe in God? What is your relationship with God? What aspects of your spirituality or spiritual practice help you the most? (prayer, meditation, readings, attending religious services?)
A - Action in treatment How would you like your doctor to consider the religiosity/spirituality question in your treatment? Name any religious/spiritual leaders in your community.	E - Effects on treatment Are there any spiritual resources you are missing? Are there any restrictions on your treatment generated by your beliefs?

Studies evaluating the association of spirituality and religiosity with CV outcomes have been criticized for the difficulty in adjusting for multiple comparisons, certain seemingly contradictory findings, and too many instruments. Measuring spirituality is complex because of the many aspects involved in defining it and the multiple domains it encompasses.

Systematic reviews^386,396,397^ broadly discuss the tools available for assessing spirituality and religiosity, showing that the different instruments measure a wide range of spiritual dimensions, including religious denomination, attendance at religious ceremonies, OR, NOR and IR, religious/spiritual coping, religious and spiritual beliefs, practices and values, well-being and inner peace, stress generated by religion (“struggle”), a tendency towards forgiveness and gratitude.

The scale called Brief Multidimensional Measure of Religiousness and Spirituality, validated in Brazil,^392^ considers in its analyses the frequency of spiritual experiences, values/beliefs, propensity for forgiveness, personal religious practices, religious and spiritual overcoming, religious support and commitment.

The WHO Quality of Life instrument in the Spirituality, Religiosity and Personal Beliefs module (WHOQOL-SRPB) comprises 32 items, distributed in 8 facets involving connection to being or spiritual strength, meaning in life, wonder, wholeness and integration, spiritual strength, inner peace, hope, optimism and faith.^398^

In a systematic review, Lucchetti et al.,^386^ selected and evaluated instruments for clinical research validated in Portuguese.

#### 9.2.5. Attitudes and Behaviors after Spiritual Anamnesis

With information on the spiritual dimension of patients, it is possible to establish new possibilities for understanding the pathophysiology illness and consequent medical intervention. Some general lines can be established:

1. Take no action: religious issues are delicate and not always objective to the point of plausible resolution, even though they may be of great importance to the patient. Often, the best course is simply to offer your empathy and understanding;
2. Incorporate spirituality in preventive health: the physician can encourage the patient to use his/her spirituality as a disease prevention tool by engaging in activities such as prayer and meditation;
3. Include spirituality in adjuvant treatment: the physician can help the patient identify spiritual aspects that, along with standard treatment, may help with the outcome of the disease; in the case of serious illness, the physician can collect the spiritual history and help the patient find meaning, accept the illness, and cope with the situation using his/her spiritual resources in the best way;
4. Modify the treatment plan: it is up to the physician to understand that the patient has the freedom to be able to modify the therapeutic plan on the basis of religious beliefs and thus to propose modifications in the course of the treatment. For example, the patient may opt for meditation as an option for chronic pain, change chemotherapy plans, and seek community support.

### 9.3. Primary Prevention

Available scientific evidence describes that high levels of spirituality and religiosity are associated with lower prevalence of smoking, alcohol consumption, sedentarism/PA, better nutritional and pharmacological adherence in dyslipidemia, hypertension, obesity and DM.^365,399-401^

**Alcohol:** In many studies that examined the relationship between spirituality and religiosity with alcohol use, an inverse relationship was found; that is, there were higher rates of spirituality or frequency of religious activity, with lower alcohol consumption. According to the same authors, several studies have shown that more religious individuals are more physically active. There is also a positive relationship between spirituality and religiosity and PA.^365^ Among Brazilian university students, a higher prevalence of alcohol consumption, smoking and use of at least one illicit drug in the last 30 days among those who had less frequent religious involvement.^399^

**Smoking:** In the CARDIA cohort study, it was observed that religiosity was related to lower risk of subclinical carotid atherosclerosis and had a positive association with higher consumption of fiber, vegetables and fruits, and lower consumption of processed foods.^401,402^

**Obesity:** In both the MESA and CARDIA studies, a higher association was observed between extent of religious involvement and higher propensity for obesity.^401,402^ Compared to those who did not participate in any religious activity, individuals with different frequencies of religious involvement were significantly more prone to obesity even after adjusting for demographic and smoking characteristics.

**Diabetes mellitus:** Regarding diabetes, although they are more prone to obesity, patients with greater religiosity had no higher risk of being diabetic. This may be explained by better diet or better treatment adherence.^366^ In contrast, in the Third National Health and Nutrition Examination Survey (NHANES III) study, there was no association between diabetes and attendance at religious services.^370^

**Hypertension:** Regarding hypertension, the results are contradictory. In the Chicago Community Adult Health Study, it was found that higher religiosity indicators were not associated with hypertension.^403^ In the prospective Black Women’s Health Study, after an 8-year follow-up, the greater involvement with spirituality and religiosity employed in coping with stressful events was associated with a lower risk of developing hypertension, especially in women with higher stress.^404^ A national study involving a highly religious community found that the prevalence of hypertension among these individuals was lower than the national prevalence.^405^

Meditation is one of the most studied interventions among practices related to spirituality and religiosity and the repercussions on BP levels. In these studies, the magnitude of BP reduction varies significantly. Studies have methodological limitations with data bias, high dropout rates, and different populations studied.

In a systematic literature review, transcendental meditation reduced SBP by ~4 mmHg and DBP by ~2 mmHg, effects comparable to other lifestyle interventions such as weight loss and exercise.^407^ The mechanisms by which meditation reduces PA have not yet been fully elucidated. Possibly, the long-term neurophysiological changes that occur with meditation may lead to changes mediated by the autonomic nervous system in BP. The impact of stress reduction on BP remains to be better defined.^406^

### 9.4. Secondary Prevention

As with primary prevention, secondary prevention should be viewed as comprehensive and taking into account psychosocial factors such as socioeconomic status, depression, anxiety, hostility/anger, and type D personality that may aggravate CVD.^2^ In this context, some of these factors should be highlighted, as well as the results obtained with new proposals for intervention in the field of spirituality, religiosity and related areas.

**Forgiveness:** Evaluated by various scales as tendency and attitude, forgiveness determines multiple effects, generating states more favorable to homeostasis in the emotional, cognitive, physiological, psychological, and spiritual aspects. Forgiveness broadens the possibilities for behavior by building better adaptive strategies and counteracting the feelings of anxiety, anger, and hostility that are potent CV risk factors. It also reduces stress, drug addiction and rumination; improves social support, interpersonal relationships, and health self-care.^409-413^

One study analyzed the effect of forgiveness on myocardial ischemia, ischemia generated by stress and measured by scintigraphy techniques. Patients were randomized to receive or not a series of psychotherapy sessions to develop interpersonal forgiveness. After 10 weeks of follow-up, the forgiveness intervention was able to reduce the burden of anger-induced myocardial ischemia in patients with CAD.^414^

**Gratitude:** In clinical practice, gratitude can be assessed by specific questionnaires such as the Gratitude Questionnaire - 6 (GQ-6),^415^ allowing the analysis of behavioral interactions and physiological, pathophysiological and clinical outcomes. Individuals with greater gratitude have a better CV health profile, similarly to those with higher spirituality and religiosity indices.

In asymptomatic HF patients assessed by the gratitude, depression, sleep, gratitude, and spiritual well-being questionnaires, the latter two correlated with better inflammatory profile and better mood and sleep quality, less fatigue, and greater self-efficacy.^416^ Psychological strategies that may increase feelings of gratitude such as regular journaling, thoughts, meditation, and fact-checking, or grateful people have been studied, demonstrating increased feelings of gratitude and reduced inflammatory markers.^417^

**Depression and Resilience:** Depression is significantly more common in patients with CVD than in the general community. This higher prevalence is often secondary to the disease as an adaptation disorder, with symptoms disappearing spontaneously in most patients. However, approximately 15% of them develop a major depressive disorder, which is an independent risk marker of increased morbidity and mortality.^418,419^

In a cross-sectional study including 133 patients diagnosed with ischemic heart disease assessed by the Wagnild & Young Resilience Scale, 81% were classified as resilient, suggesting that disease may act as a facilitator for the presence of this feeling.^420^

Resilience is a behavior that greatly improves treatment adherence as well as quality of life and can be acquired at any stage of life, regardless of age and disease state. Spirituality and religiosity are associated with higher levels of resilience.^420,421^

In another series, elderly patients (≥ 65 years) were significantly more resilient than younger patients. Resilience correlated negatively with depression and inversely with affective, cognitive, and somatic symptoms of depression and was responsible for greater variation in the affective characteristics of depression than in the somatic characteristics.^419^

In a long-term cohort, patients were analyzed for functional social support, BMI, recent history of major depression, coronary artery disease, hypertension, and diabetes. After 13 years, it was observed that social support was responsible for reducing the relationship between depression and the occurrence of coronary artery disease. Specifically, depression was prospectively associated with coronary artery disease among individuals with low social support but not those with high support, suggesting that it may function as a resilience factor against CV risk associated with depression.^422^

The Palliative Care in Heart Failure study was the first randomized controlled trial involving palliative care to demonstrate the significant clinical benefit of incorporating interdisciplinary interventions in the management of patients with advanced HF. The addition of palliative care improved physical and psychosocial condition (anxiety/depression), and spiritual quality of life.^423^

**Relaxation and Meditation:** Relaxation and meditation are well-established mind/body approaches to improving stress, and their benefit has been demonstrated in many populations, including heart disease.^344^^.^^424-426^ Easy to learn and practical, they are inexpensive and widely accessible techniques.

In an observational study in patients with coronary artery disease, the cardiac rehabilitation strategy associated with a 13-week program was analyzed using self-relaxation, spiritual well-being and psychological stress control techniques. There were significant increases in relaxation practice time and spiritual well-being scores, as well as improvement in depression, anxiety, hostility, and overall severity scores. A greater increase in relaxation practice time was associated with spiritual well-being, which in turn was associated with improved psychological outcomes.^424^

Patients with coronary artery disease were enrolled in a transcendental meditation or health education program with an average follow-up of 5.4 years. Transcendental meditation significantly reduced the risk of mortality, myocardial infarction, and stroke, these changes being associated with lower BP levels and psychosocial stressors.

Additionally, a national study randomized patients with chronic HF to do meditation or not, demonstrating a reduction in serum norepinephrine and VE/VCO_2_ slope in the cardiopulmonary test and improved quality of life assessed by the Minnesota Living with Heart Failure questionnaire.^426^

A recent paper by the American Heart Association reviews various forms of meditation and highlights the prolonged effects observed on brain physiology and anatomy, possibly responsible for better systemic physiological status and reduced cardiovascular risk. Meditation shows a better physiological response to stress, smoking cessation, reduced BP, insulin resistance and metabolic syndrome, endothelial function, inducible myocardial ischemia, and primary and secondary prevention of CVD. Although some data on CVD risk reduction are limited, meditation can be considered as a complement to risk reduction and lifestyle modification.^344^

In a robust study involving 1,120 meditators, other complex domains have been identified that may be crucial to people’s psychological and spiritual development by acting as mediators and/or mechanisms responsible for the effects of meditation. Of difficult measurement, relational and transpersonal aspects, mystics, and anomalous or extraordinary phenomena linked to meditation deserve further study.

The extent of the possible effects to be obtained with each form of meditation remains open. Transcendental meditation has been shown to reduce anxiety, improve mood, and to double the acute pain tolerance time when compared to secular forms of meditation.^428^

**Medication adherence:** In a cohort of 130 HF patients, adequate adherence score was observed in only 38.5% of patients. Spirituality, religiosity, and personal beliefs were the only variables consistently associated with adherence. It is noteworthy that depression or religiosity were not correlated with adherence when evaluated separately. When spirituality was assessed by both, it was positively correlated with adherence, adjusted for demographic and clinical characteristics and psychosocial instruments.^429^

**Cardiac Rehabilitation:** Several studies report improvement in psychological stress in patients with coronary artery disease undergoing CV rehabilitation. In addition, a meta-analysis of 23 randomized controlled trials involving 3,180 coronary artery disease patients seeking to assess the impact of adding psychosocial interventions to standard rehabilitation exercise reported greater reduction in psychological distress and improvements in SBP and serum cholesterol.^430^ The scope of cardiac rehabilitation can be amplified by positive psychology techniques. In patients undergoing coronary angioplasty, these explanatory techniques, with telephone contacts and inductive correspondence, resulted in better physical performance (caloric expenditure), with a reduction in medical events, as opposed to the effects observed by stress.^431^

In another meta-analysis, the influence of rehabilitation associated with psychosocial and/or educational interventions in 14,486 individuals with pre-established coronary artery disease with a median follow-up of 12 months was evaluated. In general, rehabilitation led to a reduction in CVD mortality and the risk of hospitalizations and a better quality of life.^354^

### 9.5. Recommendations for Clinical Practice

Most patients and their families, guardians or caregivers have varying degrees of religiosity and spiritual needs and, importantly, expect health professionals to know their beliefs and to be part of the decision-making process, reinforcing the concept of integrality.

Health professionals involved should keep in mind that spirituality and religiosity favorably influence ability to cope with the disease, but the isolation imposed by hospitalization may be negative as it removes patients from their religious meetings or practices, from religious leaders and of dedicated communities.

These beliefs and practices can impact and often antagonize proposed medical strategies. It is worth noting that health professionals also present their own profiles of spirituality and religiosity, influencing their practice, especially in severe, critical or limiting situations.

Every professional should be aware of the relevance of screening involving spirituality, and those focused on direct care such as doctors, nurses, and chaplains should have an anamnesis of spirituality and religiosity, viewed not only as part of identifying where professed religion is concerned, but how a broader construction obtained by structured or unstructured questionnaires, allowing to penetrate and understand the true identity of spirituality and religiosity in patients and relatives.^387,432,433^ Most professionals are sensitive to the demand of patients only when informed, but the contemporary view is to actively search for this information and demands, because the patient often does not feel comfortable discussing it.^432^

In a holistic view of a human being, the anamnesis of spirituality and religiosity should be remembered in each care interaction and by all health professionals.^366,433^ Naturally, this approach may be unimportant or difficult to use in many situations, such as in major emergencies, but it is of enormous relevance in critical, terminal, chronic degenerative diseases or palliative care.

Critically ill patients have high rates of not only pain, dyspnea, anorexia, and fatigue, but also of anxiety, nervousness, sadness and depression. For this patient profile, Cicely Saunders’ concept of “total pain,” understood as a sum of physical, psychological, social, emotional and spiritual elements, must be valued and addressed in a systematic and structured manner, even in the first days of hospitalization.^434,435^

Patient-centered approaches with a greater focus on spirituality make it easy to understand and value the motivations for consultation, understand the patient’s universe (including emotional and existential issues), and strengthen the relationship between practitioners and patients, shared decision making, and prevention and promotion of health.^433,436^

It is essential for professionals to be technically prepared and the patient to agree on addressing issues related to spirituality and religiosity so that the interaction is constructive and without conflict. In the absence of technical training or resistance to the subject by the patient, the spiritual history should be postponed to a more opportune time or even canceled. When these alignments do not occur, serious conflicts can develop and sometimes very deleterious to medical management.

To avoid conflicts in the doctor-patient relationship, it should always be borne in mind that this area is deeply personal as well as intensely emotional, and therefore, the physician should not address emotional issues without proper approximation of spiritual and/or religious aspects. The physician should be sure of the patient’s agreement to address the issue.

Health professionals, especially those involved in critical or terminal patient care or palliative care, are subject to a significant burden of professional stress. This work involves a lot of compassion, understood as an attitude of addressing the needs of others and helping those in distress, which can be viewed as a spiritual practice. Training and practice strategies on spirituality and religiosity in this setting can contribute to a better sense of meaning and purpose at work, spiritual well-being, less fatigue and reduced burnout.^366^

The reasons for professionals not addressing spirituality and religiosity are diverse, such as feeling uncertain about initiating spiritual discussions, being misunderstood as imposing religion, invasion of privacy, causing discomfort, difficulties with the language of spirituality.^436^ These justifications have also been identified in Brazilian medical students^437^ and represent weaknesses in medical education and practice, with specific ignorance or inadequate dimensioning, lack of mastery of specific tools and training.

The solution to these limitations lies in the development of hospital spirituality support and training programs. These programs contribute to well-being and health improvement, assist with misunderstanding in conducts, and meet patients’ expectations, and they are part of accreditation processes and prospects for reducing hospitalization costs.^438^ For the development of these programs, there should be deep institutional involvement, formal training of the teams most directly connected to care, availability of infrastructure and resources, adjustments to care routines, and alignment with the various religious communities.

Health teams, especially when acting in scenarios where there is a higher demand for spirituality and religiosity, should be structured with systematic training and clear definition of responsibilities, such as obtaining and recording anamnesis in medical records, clarifying the observed demands and the implemented clinical course, as well as the observed outcomes. At initial contact, spiritual history can be obtained through open and brief questions by the doctor, nurse or chaplain, thus tracking needs and anticipating conflicts. For the spiritual approach, no professional is expected to be able to do so, but a certified chaplain or a spiritual care professional with equivalent technical training and structured standards and concepts to develop a spiritual care plan.^387^

Religion should never be prescribed, forced or even encouraged, at the risk of adding guilt to the burden of disease. Identifying the right time for spirituality and religiosity approaches is important to avoid any kind of misunderstanding, always under the rule of common sense. We emphasize that the evaluation of spirituality is always desirable, enabling the search for information in all patients regardless of religion or religiosity, but the approach in extreme situations can lead to stress and even worsen patient evolution.

Respect for spirituality, religiosity and individual beliefs is essential and should match the therapeutic plan if it is not harmful. If necessary and at the patient’s wishes and in the face of risk or harm or in conflict situations, the presence of religious representatives or leaders can bring comfort, balance and better management and can contribute to a desired consensus.

The approach of spirituality and religiosity topics in medical consultation in an area such as cardiology, where the patient is generally in a situation of fragility, and more sensitive and stressful, increases the complexity of the multiple variables already mentioned and may generate some conflicts. The misunderstanding or intolerance of the parties involved are major factors and can generate conflicts of various kinds and at all interfaces involving the patients, their families and their relationships, within the multidisciplinary team itself and between the team and the patient. All these problems can be prevented by good management of the doctor-patient relationship which, once consolidated, will make all other situations less influential.

Conflicts can be avoided even for untrained professionals, as long as some important steps are followed: conduct spiritual anamnesis without prejudice, showing deep interest and respect for the patient, seeking to understand their religion, beliefs, and practices,^363^ encourage questions to help patients clarify their feelings and thoughts about the spiritual perspective of what is going on or even their possible spiritual problems.^393^ In questions related to spirituality and religiosity, it should be kept in mind that it is always better to understand than to advise.

Concepts involving evidence-based medicine have also been applied in the realm of spirituality, but the available evidence is not always ideal and definitive. In these scenarios, available evidence should be used to improve this practice, also contributing to the revision of old concepts, the development of new research and the advancement of science in the field of spirituality. In Chart 9.4, GEMCA gathers recommendations that may be useful for improving cardiology practice in our country.

**Chart 9.4:** Practices in spirituality and health. Recommendation class and level of evidence

Recommendation	Recommendation class	Level of evidence	References
Brief tracking of spirituality and religiosity.	I	B	^388-391,429^
Spiritual anamnesis of patients with chronic diseases or with poor prognosis.	I	B	^386,387,393,429,432^
Respect and support the patient's religions, beliefs and personal rituals that are not harmful to treatment.	I	C	^361,365,366^^,^^384^
Support from trained professional for patients suffering or with spiritual demands.	I	C	^361,365,366,393^
Organizational religiosity is associated with reduced mortality	I	B	^370,371,373,375^
Hospital program for training in spirituality and religiosity.	IIa	C	^365,438^
Spiritual anamnesis of stable individuals or outpatients.	IIa	B	^384,386,387^
DUREL, FICA, HOPE, or FAITH questionnaire to assess spirituality.	IIa	B	^380,386,393,394^
Meditation, relaxation techniques and stress management.	IIa	B	^406,424-426^
Spirituality and religiosity potentially increase survival.	IIa	B	^370,371,373,375^
Spiritual empowerment techniques such as forgiveness, gratitude and resilience.	IIb	C	^412,413,417-420^
Evaluate spirituality and religiosity in patients in acute and unstable situations.	III	C	^384,387,439^
Prescribe prayers, religious practices or specific religion.	III	C	^365,381,382^

## 10. Associated Diseases, Socioeconomic and Environmental Factors in Cardiovascular Prevention

### 10.1. Introduction

In the last century, humanity has undergone an epidemiological transition in relation to the causes of death; infectious diseases are no longer the leading cause of death while chronic degenerative diseases, especially CVD now take the lead. Although they are still the leading causes of mortality worldwide, from the late 1950s, a decline in CVD mortality began in industrialized countries. In Brazil, this decrease in CVD mortality began to be observed in the late 1970s, with a significant reduction in these rates, despite significant regional differences.^2,440,441^

It is not possible to only associate the reduction in mortality due to CVD to the better control of classic CV risk factors such as diabetes, hypertension, obesity, dyslipidemia and smoking, since all of these, except smoking, have increased in prevalence in recent decades. This led to new concepts about occupational, behavioral and environmental risk factors, which are directly influenced by the socioeconomic conditions of the populations and have an important relationship with the causes of mortality.

In this chapter, we describe important conditions associated with increased CV risk that require concomitant assessment with classic CV risk factors when addressing CVD as a complex relationship between patients and the context in which they live.

### 10.2. Socioeconomic Factors and Cardiovascular Risk

The health conditions of populations are influenced in a complex way by social determinants such as income distribution, wealth and education. These indicators act as interdependent risk factors for disease occurrence. Relationships between mortality rates and socioeconomic level have already been evidenced in Brazil and other countries, showing an inverse relationship, i.e., low socioeconomic levels are related to high mortality rates. These relationships between reductions in mortality rates, in particular deaths from diseases of the circulatory system (DCS), and improvement in socioeconomic indicators are highly correlated. Several prospective studies have shown that low socioeconomic status, defined as low educational level, low income, low status employment, or living in poorer residential areas, have contributed to the increase in all causes of death, as well as the risk of death from CVD.^9,442-446^

Low socioeconomic status, when defined as an independent CV risk factor, has been shown to confer an increased risk for CVD; with RR mortality between 1.3 and 2.0.^445,447^ The time periods in which there was a reduction in mortality rates due to diseases of the circulatory system were preceded by periods with improvement in socioeconomic indicators. In Brazil, between the 1930s and 1980s, there was great economic growth that, despite the concentration of income, enabled educational, sanitary, economic and infrastructure improvements, reducing infectious diseases and inflammatory processes. In developed countries, the decline in CVD mortality began a little over a decade after the end of World War II, which followed the great depression of the early 1930s and the 1918 influenza pandemic. The same decline began just over 40 years after the beginning of the period of economic growth. Exposure to infectious agents and other unhealthy conditions in the early years of life may make individuals more susceptible to the development of atherothrombogenesis. It is also possible that the reduction in exposure to infectious diseases in the early stages of life is related to the observed decline in adult CV mortality.^442,446,448-452^

Strong correlations have been shown between the Human Development Index, falling child mortality, rising per capita gross domestic product (GDP) and the increasing education levels; with the reduction in mortality from diseases of the circulatory system in adults, from 1980, in some Brazilian states and municipalities, showing that the improvement in socioeconomic indicators preceded the reduction of CV deaths. The great increase in education over the last decades, which practically doubled in the states of Rio de Janeiro, São Paulo and Rio Grande do Sul, had a great impact on mortality, and is related to the reduction of more than 100 deaths from CVD with a one-year increase in average years of study in adults. Comprehensive measures to improve socioeconomic indicators should be part of the paradigm for CV disease control. These relationships show the importance of improving the living conditions of the population in order to reduce CV mortality.^442,446,448,449,453,454^ The assessment of social factors in patients and people with CV risk factors is essential as a means to stratify future preventive efforts with individual’s risk profile.

Recommendations for socioeconomic indicators and CV risk are listed in Table 10.1.

**Table 10.1:** Socioeconomic indicators and cardiovascular risk

Recommendation	Recommendation Class	Level of Evidence	References
Socioeconomic indicators should be investigated in the clinical assessment and considered in the patient approach to improve the quality of life and prognosis of circulatory system diseases.	IIb	B	^483,484,486,488^

### 10.3. Environmental Factors and Cardiovascular Risk

Atherosclerosis has a complex and multifactorial pathophysiology, depending on the integration of several factors inherent to the individual, acquired or not, with the environment in which he is inserted. The impact of environmental factors on the epidemiology of CV disease has been increasingly studied and recognized, especially in relation to the possibility of adopting preventive strategies. In this context, in addition to the influence of socioeconomic factors such as income and education, the characteristics of the individual’s own habitat and lifestyle are also considered. Thus, the natural and social environments are two different types that potentially influence CV disease.^455^

The natural environment is determined by specificites of the place where the individual resides such as altitude and latitude, density of wooded areas, seasons, exposure to sunlight and atmospheric temperature. A study by Massa et al.,^456^ in the city of São Paulo in 2010, suggested an inverse relationship between green area density and CV risk, regardless of income.^456^ In addition, CVD lethality appears to be higher in winter months, while in some places there is an increase of up to 53% in the incidence of AMI.^457^ This increase occurs similarly in young adults (< 55 years) and in elderly (> 75 years) individuals, and may be a consequence of both hemodynamic variations. (e.g., elevated BP), as well as the higher incidence of respiratory infections at this time (e.g., influenza), which are known to increase the risk of heart attack.^455^ However, elevated temperatures are also associated with a higher CVD risk, especially when there is an abrupt variation in temperature.^458^

The social environment is related to the artificial forms of housing and the characteristic of daily life in modern society, especially in the urban environment. Population, noise level, violence, access to clean water, sanitation and air pollution may limit health promotion and promote the development of infectious and chronic diseases. In this context, air pollution was established as the most important modifiable environmental determinant of CVD risk, consisting of a complex mixture of gaseous particles and components.^459^

Among such pollutants, particulate matter (PM) is the element that is most relevant to health, which is formed by substances whose size and types of particles vary over time in the same region. The main sources of PM are motor vehicle emissions, tire fragmentation and reuse in asphalt production, energy industry-related combustion, ore processing, agriculture, construction and demolition activities, forest burning and volcanic eruptions, among others.^460^ Thus, because of the complexity related to their composition, the particles are identified according to their diameter: coarse PM or PM10 (< 10 and ≥ 2.5 µm); Fine PM or PM2.5 (< 2.5 and ≥ 0.1 µm); Ultrathin PM or PM0.1 (< 0.1 µm).^459^

Current evidence suggests that PM2.5 is the major pollutant associated with increased CVD risk for both fatal and nonfatal events. The central justification for this relationship is the increased oxidative stress and systemic inflammation promoted by the particles. These effects result in the amplification of other traditional risk factors already present and in the potential instability of coronary plaques.^461^ According to the WHO, the mean daily PM2.5 concentration should be < 20 µm/m^3^, and the annual < 10 µm/m^3^. With each 10µm/m^3^ increase in short-term exposure, there is a 2.5, 1 and 2.1% increase in the risks of admission or death from AMI, stroke and HF, respectively. However, as exposure tends to occur over several years, atherosclerosis becomes progressive and cumulative, and also affects regional CV mortality. Thus, recurrent events may occur even with average annual PM2.5 concentrations below the WHO targets. Other consequences possibly associated with short- and long-term pollution are venous thromboembolism, acute atrial fibrillation, hypertension, and insulin resistance.^459^

The recommendations for environmental indicators and CVD risk can be seen in Table 10.2.

**Table 10.2:** Environmental indicators and cardiovascular risk

Recommendation	Recommendation class	Level of evidence	References
Restrict exposure to air pollution as a non-pharmacological measure for primary and secondary prevention of cardiovascular events	I	B	^459-461^

### 10.4. Vaccination for People with Heart Disease

In most clinical situations, vaccination is identified as a primary prevention action. When it is transposed to heart disease, it is usually secondary prevention for decompensations that aggravate pre-existing CV disease. Several vaccines are indicated for adults, with priority to patients with NCD, such as heart disease. We will list those prescribed for adults with heart disease. There is a specific guideline published by the Brazilian Society of Cardiology regarding indications and doses of vaccines indicated for children and adolescents with heart disease.

#### 10.4.1. Prevention of Respiratory Tract Infections in People with Heart Disease

Historical reports evidence the seasonal relationship of influenza epidemics with higher mortality among the elderly and patients with NCD. Observational trials, reports, population studies and meta-analyzes have proven the benefits of vaccination against respiratory infections in the elderly and in patients with NCD, with a marked reduction in overall mortality, hospitalizations, myocardial infarction and stroke rates.^463-471^ Venous congestion and immunosuppression present in patients with NCD who are predisposed to infections are highlighted among the pathophysiological explanations. In contrast, infections cause changes in coagulation factors, platelet aggregation, inflammatory response proteins, tumor necrosis factor and cytokines, and thus may be triggers for acute CV events. Infections also play a chronic role in decreasing cardiomyocyte contraction strength, inflammation, thrombosis, fibrin deposition, and acceleration of the atherosclerosis process and cardiac remodeling.^463,468,471^ Despite all the evidence and guidelines, the rate of vaccination against respiratory infections - Influenza and pneumococcal pneumonia - are low in Brazil and worldwide.^472-474^

The overall consensus is for all patients with heart disease and NCDs to be vaccinated, regardless of age; they are summarized in Chart 10.1 and Table 10.3. If the patient is over 60, the patient will be included in government campaigns according to age group. If the patient is under 60 years of age, a referral form is required along with a declaration that there is a clinical indication for vaccination.

**Chart 10.1:** Main priority indications for influenza vaccination and pneumococcal vaccine

System	Syndromes, diseases or clinical situations
**Cardiovascular**	Stroke
	Congenital heart disease
	Valvular heart disease
	Coronary Artery Disease (Angina pectoris, Myocardial Infarction)
	Pulmonary hypertension
	Systemic hypertension if target organ injury
	Heart failure and cardiomyopathies
**Respiratory**	Asthma
	Bronchiectasis
	Bronchopulmonary dysplasia
	Interstitial lung disease
	Chronic Obstructive Pulmonary Disease (COPD)
	Cystic fibrosis
**Endocrine**	Diabetes mellitus
	Grade 3 Obesity
**Gastrointestinal**	Cirrhotics
	Chronic liver disease
**Other**	Chronic kidney disease (stages 3,4 and 5)
	Down Syndrome
	Solid organ transplant
	Over 60 years old, even if healthy

Source: Martins WA.^477^

**Table 10.3:** Indication of vaccination in heart disease

Recommendation	Recommendation class	Level of evidence	References
Vaccine heart disease patients against influenza to reduce morbidity and mortality	I	B	^463-471^
Vaccine heart disease patients against pneumococcus to reduce morbidity and mortality	I	C	^475,476,478^
Vaccine heart disease patients with other vaccines recommended for adults (Hepatitis, Triple Viral, Diphtheria and Tetanus)	I	C	^475-477^
Yellow fever vaccine for people over 60 years old, with or without heart disease, at high risk of exposure to the disease against Yellow Fever	IIa	C	^475-477,479^
Yellow fever vaccine for people older than 60 years, with or without heart against at low risk of exposure to the disease	III	C	^475-477,479^

#### 10.4.2. Which Vaccines?

***Influenza Vaccine:*** In Brazil, it is up to the Ministry of Health to determine the composition of the vaccine according to the prevalence of circulating types and strains in recent epidemics. It is an inactivated, trivalent or tetravalent vaccine, with the latter having a greater immunization spectrum. Indications, characteristics and restrictions are common to trivalent and tetravalent. Vaccination should occur annually in the national campaign, which takes place between April and May.^475-477^

***Pneumococcal Vaccine:*** There are two types of vaccine: conjugate and polysaccharide. Among the conjugates is “Pneumo 10” which is intended to prevent serious infections in children under 2 years of age; therefore outside the scope of NCDs, with the exception of congenital heart disease. The other available type which is widely used is the “Pneumo 23”. This vaccine contains 23 pneumococcal serotypes and is indicated for those older than 60 years and those with clinical conditions which put them at risk for pneumonia, including those with NCD. Conjugate vaccines have shown better performance in clinical work, but are not always available in the public network. Referral for vaccination after confirmation of diagnosis. Recommended revaccination time is five years.^475-478^

***Other vaccines indicated for adolescents and adults with NCDs:*** The other vaccines indicated for adults should not be neglected for those with heart disease. Among them is the Hepatitis B vaccine, for which three doses are recommended in patients up to 49 years of age, depending on the previous vaccination situation. Regarding the triple virus, two doses up to 29 years of age and one dose over 30 years are indicated, with an age limit of 49 years. Older people and patients with heart disease are susceptible to falls and injuries, and therefore the double vaccine is recommended, DT (Diphtheria and Tetanus), with a booster vaccine every 10 years.^475-477^

***Yellow Fever:*** There is limited evidence regarding the safety of the yellow fever vaccination in heart disease patients and those over 60 years of age. There are two prospective studies and one report suggesting that serious adverse effects are rare in this age group, but much more frequent than in young people. There is limited data available on the relationship between the risk of adverse effects and the presence of previous CVD disease; interaction with CVD drugs; and the use of the fractional doses currently adopted in Brazil. Therefore, vaccination is recommended for those at risk of exposure to the disease, such as the elderly and heart disease patients. The vaccine should be given as a single dose without the need for a booster vaccine.^475-477,479^

***Vaccination Precautions:*** The use of platelet antiaggregants is not an impediment to the use of intramuscular vaccines, thus there is no need for suspension. Subcutaneous vaccination can be performed in patients taking warfarin anticoagulation or direct anticoagulants. There are no reports of clinically significant interactions of vaccination in patients using antihypertensives, anti-ischemics, statins, fibrate, warfarin or digoxin.^475,476,480,481^

### 10.5. Lower Extremity Peripheral Artery Disease

#### 10.5.1. Context

The evolution of atheromatous plaque and its association with the various CVD risk factors is widely described in the literature. It is also recognized that the atherosclerotic phenomenon can occur in different vascular beds, of larger or smaller caliber. The term peripheral arterial disease (PAD) has been used to characterize atherosclerotic disease that affects several peripheral (non-coronary) vascular beds. In this context, the current PAD guidelines deal with the theme in different ways. While the European directive,^482^ has chosen to analyze PAD in various vascular territories (i.e., carotid, subclavian, mesenteric, renal, and lower limb arteries), the current American document,^483^ as well as the Society for Vascular directive Surgery,^484^ deals exclusively with lower extremity PAD.

More than 200 million people worldwide are estimated to have diverse stages of PAD, ranging from the asymptomatic phase of the disease to intermittent claudication (IC) and the more severe late stages of the disease.^485^ Prevalence increases with aging, rising by more than 10% in patients aged 60 to 70 years; and over 20% in patients over 80 years of age. Although the prevalence of symptomatic and more severe forms of PAD is higher in men, a recent study of 3.6 million individuals in the US has shown that women may be worsening the odds of developing the disease compared to men (odds ratio [OR] of 1.62, 95% confidence interval between 1.60-1.64). Inversely, women were less prone to carotid stenosis or abdominal aortic aneurysm (AAA) than men.^485,486^ Publications of previous decades already evidenced that the simultaneous presence of PAD and CVD or cerebrovascular is frequent, especially at older ages. Likewise, the anatomopathological characteristics and clinical manifestations of CVD in patients with PAD are usually more severe, with a higher occurrence of multivessel coronary branch injury and a higher prevalence of left coronary trunk injury.^487,488^

#### 10.5.2. Interrelationship between the Various Cardiovascular Risk Factors and Lower Extremity Peripheral Artery Disease

In most studies, the proportion of symptomatic patients ranges from 20 to 33%, among all patients with PAD. In the Swedish population aged 60-90 years, the prevalence of lower extremity PAD was 18% and intermittent claudication 7%.^489^ In Brazil, a multicenter cross-sectional study evaluated 1,170 individuals in 72 urban centers. The prevalence of intermittent claudication was 9% among those with ABI below the cutoff point of 0.9. In this analysis, women with coronary artery disease were 4.9 times more at risk for lower extremity PAD.^490^

***Arterial Hypertension:*** Hypertension increases the chance of lower extremity PAD by 32% up to 2.2 times in various epidemiological studies. Although the risk of hypertension causing lower extremity PAD has been modest in some studies, the high prevalence of this risk factor among the elderly reinforces the epidemiological burden of lower limb arteriopathy.^489^ A comprehensive study of more than 4.2 million individuals in primary health care in the UK, investigated the association between hypertension and the risk of lower extremity PAD. In this study, with each 20 mmHg increase in systolic blood pressure in hypertensive men aged 40-79 years, the risk of lower extremity PAD increased by 63%. Lower extremity PAD was associated with an increased risk of ischemic heart disease, CKD, HF, aortic aneurysm and atrial fibrillation; however, it was not associated with hemorrhagic stroke.^491^

The Harmonica Project, a Finnish community-based report, showed that the prevalence of asymptomatic (without claudication) lower extremity PAD, by means of ABI, was 7.3% in 532 hypertensive subjects, compared with 2.3% in 440 normotensive individuals. By adjusting multiple variables, hypertension continued to be an independent risk factor associated with lower extremity PAD, more than tripling the occurrence of lower limb arterial involvement (OR: 3.20). Hypertensive patients with borderline and altered ABI represented one third of all participants with hypertension in the average age range of 60 ± 7 years.^492^

***Smoking:*** This is a particularly prominent risk factor in atherosclerotic disease of the lower extremities. The prospective Health Professionals Follow-up Study (HPFS) investigated 44,985 men with lower extremity PAD aged 40 to 75 years with a history of limb amputation, need for revascularization, arterial angiographic injury > 50% occlusion, and ABI below 0, 90. The authors followed the attributable risk of four of the most traditional risk factors, diabetes, hypertension, hypercholesterolemia, and smoking, by a median follow-up of 24.2 years. Active smoking significantly increased the adjusted risk of lower extremity PAD by 12.89-fold (95% confidence interval between 8.59 and 19.34), compared with individuals who had never smoked. Also, in participants who stopped smoking for more than 20 years, the risk of lower extremity PAD remained 39% higher than in those who had never smoked.^493^ The Guangzhou Biobank Cohort Study (GBCS) evaluated the association between second-hand smoke exposure and lower extremity PAD in non-smokers. By adjusting for confounding variables, exposure to residential passive smoke for 25 hours / week or more was significantly associated lower extremity PAD (OR = 7.86; p = 0.003).^494^

***Diabetes:*** The presence of diabetes increases the risk of lower extremity PAD by 1.9 to 4 times compared to non-diabetics.^489^ In our country, the risk of diabetic men developing lower extremity PAD was 6.6 times higher than that of non-diabetics.^490^ A case-control trial in patients with diabetic foot investigated ulcers that progressed to amputation. After adjusting several variables, at least three widely known risk factors were predictors of amputation risk:

- HbA1c level > 8% (p = 0.002);
- hypertriglyceridemia (p = 0.004); and
- hypertension (p = 0.028).495

The risk of lower extremity PAD tends to increase with the duration and evolution of both metabolic factors, diabetes (p < 0.001) and hypercholesterolemia (p = 0.05) over time.^493^

***Dyslipidemia:*** Hypercholesterolaemia increases the risk of developing lower extremity PAD by 90% (p = 0.05).^493^ FH is an autosomal dominant condition associated with mutations in the LDL receptor-encoding gene or ApoB and PCSK-C coding genes.^9^ In a Brazilian cross-sectional study, 202 patients with heterozygous FH were compared to 524 normolipidemic controls. The prevalence of lower extremity PAD in the FH group went from 17.3 to 2.3% in the group with appropriate lipid profile (p < 0.001).^496^

Classic risk factors continue to play a relevant role when lower extremity PAD progresses to more severe forms of vascular impairment, such as critical lower limb ischemia (CLI) or acute limb ischemia. Such presentations of lower extremity PAD have a poor prognosis in terms of disability and death. The UK-based prospective population-based Oxford Vascular Study (OXVASC) analyzed the incidence of severe peripheral ischemic outcomes in 92,728 patients. Compared with the control population, the occurrence of unstable events was associated with risk factors:

- hypertension: adjusted risk of 2.75 times;
- smoking: adjusted risk of 2.14;
- diabetes: adjusted risk of 3.01; and
- CLI: adjusted risk of 5.96.^497^

#### 10.5.3. Summary of Anatomical Location of Atherosclerotic Lesions of Lower Extremity Peripheral Artery Disease

The classic document from the Inter-Society Consensus for the Management of Peripheral Arterial Disease (TASC II) has been updated to include infrapopliteal segment lesions in the anatomical classification of lower extremity PAD.^498,499^ The location of lower extremity PAD according to the affected arterial territory has been harmonized by the recent Peripheral Academic Research Consortium (PARC) document (Chart 10.2).^500^

**Chart 10.2:** Lower extremity PAD location according to PARC^500^

**Aortoiliac segment:** infrarenal abdominal aorta; common iliac arteries; internal iliac artery, external iliac artery. Distal limit is the pelvic ring or inguinal ligament.
**Femoropopliteal:** common femoral artery; deep femoral artery; superficial femoral artery; segment 1 - above the knee popliteal artery, from the Hunter canal to the proximal edge of the patella; segment 2 - from the proximal portion of the patella to the center of the knee joint space; segment 3 - below the popliteal knee artery, from the center of the knee joint space to the origin of the anterior tibial artery (distal limit).
**Tibiopedal:** tibioperoneal trunk (origin of the anterior and below tibial artery until the bifurcation of the posterior and peroneal tibial arteries); anterior tibial, posterior tibial, peroneal, dorsalis pedis, arterial plantar arch and minor arteries of the feet.

#### 10.5.4. Preventive Management of Lower Extremity Peripheral Artery Disease

Several aspects of the preventive approach to lower extremity PAD risk factors are equally applicable, both regarding the asymptomatic and symptomatic (intermittent claudication) form of the disease.^484^ The items listed below and, in particular, Chart 10.3 summarize the risk factors and proposed treatment approaches (including recommendation class / level of evidence) according to the latest international guidelines for lower extremity PAD:

**Chart 10.3:** Risk Factors / Therapeutic Conduct and their Recommendation Classes / Levels of Evidence at DAPEI according to the latest international Peripheral Artery Disease guidelines

Risk Factor / Therapeutic Management	Society for Vascular Medicine Guidelines (2015)^484^	AHA / ACC Guidelines (2016)^483^	European Society of Cardiology (ESC) Guidelines (2018)^482^
Smoking	Comprehensive preventive interventions aimed at smoking cessation in asymptomatic Lower extremity PAD, intermittent claudication and after open endovascular or surgical procedure I-A	Lower extremity PAD smoking cessation programs, including pharmacotherapy I-A	Smoking cessation is recommended in all patients with Lower extremity PAD I-B
Statins	In Lower extremity PAD with intermittent claudication I-A Optimized statin therapy is recommended for all patients with claudication and after endovascular or open surgical procedure I-A	Suitable for all patients with Lower extremity PAD I-A	Recommended statins for all patients with Lower extremity PAD I-A In patients with Lower extremity PAD it is recommended to lower LDL-c below 70 mg/dL or to decrease it by > 50% if baseline values are between 70-135 mg/dL I-C
Physical exercise	Supervised Exercises I-A Residential exercises I-B Post limb revascularization exercises for claudication I-B At least annual follow-up of claudication to check the results from exercise I-C	Treadmill test may help in functional evaluation in Lower extremity PAD IIa-B Supervised exercises in patients with claudication I-A Residential or community exercises with behavioral change techniques may be beneficial in functional improvement IIa-A In lameness patients, alternative exercises such as low intensity, painless walking may be beneficial in functional improvement IIa-A	Supervised exercises are recommended in patients with lameness. I-A Unsupervised exercise in patients with claudication I-C Healthy diet and physical activity are recommended in patients with Lower extremity PAD I-C
Antiplatelets	Use of aspirin 75-325 mg/day in claudication I-A In claudication, use of clopidogrel (75 mg/day) as an effective alternative to aspirin IIb Optimized antiplatelet therapy is recommended for all patients with claudication and after endovascular or open surgical procedure I-A Improves patency of venous and artificial lower limb vascular grafts II-B In infrainguinal endovascular intervention for lower limb claudication, aspirin with clopidogrel for at least 30 days is suggested IIb	Use of aspirin monotherapy (75-325 mg/day) or clopidogrel monotherapy in claudication (75 mg/day) reduces AMI, stroke and vascular death I-A In asymptomatic Lower extremity PAD, antiplatelet use is reasonable to prevent risk of AMI, stroke and vascular death IIa-C In asymptomatic borderline ABI Lower extremity PAD, the advantage of antiplatelets is uncertain to prevent risk of AMI, stroke and vascular death IIb-B The efficacy of dual antiplatelet therapy (aspirin + clopidogrel) in reducing risk of CV events in symptomatic Lower extremity PAD is not well established IIb-B Dual antiplatelet therapy (aspirin + clopidogrel) may be reasonable to reduce risk of lower limb events in symptomatic Lower extremity PAD following limb revascularization IIb-C	In patients with symptomatic Lower extremity PAD, antiplatelet monotherapy is indicated I-A In all patients with revascularized Lower extremity PAD, antiplatelet monotherapy is indicated I-C In infrared revascularized Lower extremity PAD, antiplatelet monotherapy is indicated I-A In patients with Lower extremity PAD requiring antiplatelet agents, clopidogrel may be preferable to aspirin IIb-B Following infrainguinal endovascular intervention with stenting for lower limb claudication, aspirin + clopidogrel for at least 30 days is suggested IIa-C After prosthetic bypass graft in infrapopliteal PAD (below the knee), the use of aspirin + clopidogrel IIb-B
Anticoagulants	They reduce the risk of limb loss and increase graft patency, but double the risk of bleeding compared with antiplatelet agents B-C Suggests against warfarin use only to reduce risk of CV events or vascular occlusions I-C	The usefulness of oral anticoagulants in maintaining patency of vascular grafts is uncertain IIb-B Anticoagulation should not be used to reduce risk of CV events in Lower extremity PAD III-A	Vitamin K antagonist may be considered after revascularization with infra-inguinal autologous venous graft. IIb-B
Antihypertensives	Optimized antihypertensive therapy is recommended for all patients with claudication and after endovascular or open surgical procedure I-A	Antihypertensive therapy recommended in hypertensive patients to reduce the risk of AMI, stroke, heart failure and CV death inLower extremity PAD I-A Use of ACE inhibitors or ARB may be effective in reducing risk of CV events in Lower extremity PAD IIa	In hypertensive patients with Lower extremity PAD it is recommended to maintain BP < 140/90 mmHg I-A The use of ACE inhibitors or ARB is considered a drug of choice in patients with Lower extremity PAD and hypertension IIa-B
Diabetes, glycemic control and hypoglycemic drugs	Hemoglobin A1C target < 7.0% in lameness if it can be achieved without hypoglycaemia I-B Recommended optimized glycemic control for all patients with claudication and after endovascular or open surgical procedure I-A	Optimized glycemic control may be beneficial in patients with critical lower extremity ischemia to reduce limb outcomes IIa-B	Strict glycemic control in diabetic patients with Lower extremity PAD I-C

ABI*: Ankle-Brachial Index; ACEI: angiotensin-converting enzyme inhibitors; AMI: acute myocardial infarction; ARB: angiotensin receptor blocker; CV: cardiovascular; CVI: Stroke; Lower extremity PAD: lower extremity peripheral arterial disease.

- Smoking cessation is recommended for people with lower extremity PAD.^482-484^
- Physical Exercise: In patients with claudication due to lower extremity PAD, a supervised exercise program is recommended to improve functional performance and quality of life.^482-484^ The comprehensive review by Olin et al.,^501^ highlights that the methodology of supervised exercise, either home or community-based, has improved considerably over the past decade.
- Antiplatelet: Despite the small sample size (n = 366), the CLIPS study showed the benefit of ASA in preventing 52% of vascular events such as AMI, stroke, pulmonary embolism, and CLI.^502^ The CAPRIE lower extremity PAD subgroup analysis, which compared clopidogrel 75mg / day and acetylsalicylic acid in the secondary prevention, showed positive results in reducing CVD death, AMI and stroke by 24% in symptomatic lower extremity PAD.^502^ The EUCLID study compared ticagrelor and clopidogrel in 13,885 symptomatic lower extremity PAD patients. There was no significant difference between drugs in preventing CVD, AMI, or stroke.^502^ The presence of increased bleeding (TIMI score) was infrequent (0.94/100-patient years) and similar in randomized patients on ticagrelor and clopidogrel (p = 0.49) .^503^
- Anticoagulants: The recent COMPASS study evaluated the cumulative risk of new outcomes one year after the occurrence of a major lower limb adverse event (MALE). The cumulative incidence 1 year after hospitalization due to MALE was 95.4%, vascular amputation was 22.9%, the risk of death was 8.7% and major CV events reached 3.8%. In this study, rivaroxaban (direct selective coagulation factor Xa inhibitor) at a dose of 2.5 mg twice daily associated with ASA reduced the incidence of MALE by 43% (p = 0.01), decreased vascular amputations by 58 % (p = 0.01), restricted peripheral vascular interventions by 24% (p = 0.03) and decreased all peripheral vascular outcomes by 24% (p = 0.02) compared to AAS monotherapy.^504^
- At present, this analysis of patients with lower extremity PAD from the COMPASS study is not characterized as a recommended treatment, but it has been relevant as a hypothesis and has reinforced the importance of further investigation on the possible role of new oral anticoagulants in the prevention of vascular events in symptomatic lower extremity PAD.
- Antihypertensives: In hypertensive patients with lower extremity PAD, strict BP control below 140/90 mmHg with first-choice drugs is recommended.
- Renin-angiotensin inhibitor drugs, such as ACE inhibitors or ARBs, when tolerated, are recommended to control BP in lower extremity PAD.^482,483^
- Hypolipidemics: Management of hypercholesterolaemia in patients with lower extremity PAD aims to keep LDL-cholesterol below 70 mg/dL or reduce it by 50% if baseline levels are between 70-135 mg / dL.^482^ Statin prescription is broadly recommended in current international guidelines.^482-484^ Statins reduce the risk of CVD and lower limb ischemic events in patients with lower extremity PAD.^482,483^
- The FOURIER study tested evolocumab (PCSK-9 inhibitor monoclonal antibody) in patients aged 40 to 85 years with a history of clinically evident atherosclerotic CVD disease. In this trial, 13.5% of patients in the evolocumab group and 12.9% in the placebo group had symptomatic lower extremity PAD (13.2% of all participants).^98^ In the subgroup analysis of patients with lower extremity PAD claudication, evolocumab reduced the primary combination of outcomes by 21% (p = 0.0098).^505^ Additional information on cholesterol-lowering drugs in lower extremity PAD will be available in the future.
- Glycemic Control: Optimized glycemic control is indicated in all diabetics with lower extremity PAD, especially those with greater severity, such as critical lower limb ischemia.^482,483,484^ The objective is to reduce ischemic events in the lower extremities.^483^

In addition to their efficacy in glycemic control, new hypoglycemic drugs have been required to demonstrate CV safety. In the EMPA-REG study, the SGLT-2 inhibitor empagliflozin reduced the risk of CV death by 38%. A recent subanalysis of this study showed that in patients with lower extremity PAD at the start of the trial, the risk of lower limb amputation in the empagliflozin group was not significantly different from placebo (HR = 0.84; 95% confidence interval 0.54 and 1.32).506 However, the CANVAS study with canagliflozin, despite a 14% reduction in the risk of the primary combined outcome (CV death, AMI and nonfatal stroke), showed almost doubling of amputations, predominantly at the toe or metatarsal level. (6.3 [canagliflozin] compared with 3.4 / 1000 patient years [placebo]; hazard ratio = 1.97; 95% confidence interval between 1.41 and 2.75).^57^ Inversely, the recent DECLARE study TIMI507, with dapagliflozin, in addition to showing reduced CVD death or hospitalizations for heart failure, did not significantly increase the risk of amputations (1.4% in the dapagliflozin group versus 1.3% in placebo; p = 0.53).^507^ While further analysis is awaited, it is important that patients using SGLT-2 inhibitors maintain routine preventive foot care and adequate hydration. Monitoring the patient with lower extremity PAD at risk of foot infections, ulcer, gangrene or osteomyelitis is critical.

Risk Factors/Therapeutic Conduct and their Recommendation Classes/ Lower extremity PAD Evidence Levels, according to the latest international Peripheral Artery Disease guidelines, are listed in Chart 10.3.

### 10.6. Autoimmune Diseases and Cardiovascular Risk

Several autoimmune diseases can affect the heart through various manifestations including arrhythmias, pericardial, myocardial and coronary artery diseases. In relation to this last complication, advances and research in the field of atherosclerosis have increasingly reinforced the participation of the immune system in its pathophysiology. The presence of lymphocytes and macrophages within atheromatous plaques suggests that inflammation is a major factor in the disease evolution cascade. This hypothesis even motivated a recent clinical trial that evaluated the effect of low dose methotrexate on the reduction of CV events in patients without autoimmune diseases but with previous infarction. Although the reduction in the primary outcome was not achieved in this study, further work in this area is still ongoing.

However, in patients with rheumatic diseases, the systemic inflammatory process is amplified and which may result in the occurrence of accelerated atherosclerosis.^509^ This condition may be the main explanation for the high percentages of morbidity and mortality in these patients.^510^ In addition, the use of certain immunosuppressive medications, such as corticosteroids, may also contribute to the worsening of the CV risk profile. It is worth mentioning RA and systemic lupus erythematosus (SLE) among the diseases that may have this pathophysiological feature, although other conditions such as scleroderma, inflammatory bowel diseases, psoriasis and certain primary vasculitis such as polyangeitis granulomatosis, are also relevant.^509-511^

RA is associated with a 3-fold reduction in survival, with ischemic heart disease accounting for about 40% of deaths.^512^ In addition, the risk of AMI is about 2 times higher than in the general population, and the prognosis after the event tends to be worse. This scenario begins to develop at the onset of the disease and independently of other factors classically associated with atherosclerosis. Vascular inflammation caused by autoimmunity seems to play a more important role in this context. Some population studies even suggest a recent reduction in CV lethality in these patients, perhaps due to the greater availability of disease-specific treatments.^513^ Nevertheless, functional limitation and consequent physical inactivity imposed by RA may also increase the likelihood of developing other risk factors, such as obesity, hypertension and diabetes. On the other hand, it is noteworthy that systemic inflammation in individuals with RA can reduce serum levels of total cholesterol and LDL, promoting what is known as the “lipid paradox”, since the risk of events remains high even with this metabolic profile.^513,514^ Nevertheless, control of traditional risk factors remains the main strategy for preventing CV events in these patients. Like RA, SLE also behaves as an independent risk factor for CV disease, with a coronary disease prevalence of up to 10% and a risk of events up to 8 times higher than the general population. Some studies suggest that AMI may be the cause of death in up to 25% of cases, especially in patients who have the disease for longer.^509^ At the same time, the prevalence of major CV risk factors such as hypertension, diabetes, obesity, physical inactivity and dyslipidemia is also higher in individuals with SLE. Frequent use of corticosteroids for disease management is another condition that worsens the metabolic profile, although daily doses of prednisone below 10 mg appear to be safe in this respect, as do antimalarial drugs.^515^ Nevertheless, risk calculators using traditional factors often underestimate the incidence of events in these patients. Other markers associated with atherosclerosis that are more relevant in individuals with SLE, such as osteoprotegerin and osteopontin, are promising predictors that could refine this estimate. The fact that disease-associated coronary artery disease is more often associated with atherosclerosis than vasculitis corroborates this expectation.^516^

As most autoimmune diseases are more common among women, a thorough stratification of CV risk in females is essential in the presence of these conditions, despite the limitations already mentioned. Even so, the fundamental issue is the absence of clinical studies demonstrating a benefit in treating this group of patients more aggressively. To date, there is no evidence that therapeutic targets for blood pressure, blood glucose, LDL cholesterol, or any other risk factor should be modified due to the presence of an autoimmune disease. The relatively low prevalence of these diseases in the population is the main factor limiting good quality studies to answer these questions. Therefore, each case needs to be individualized, with constant reassessments throughout the disease evolution of the potential risks and benefits of treatment.

Recommendations for autoimmune diseases and CV risk are shown in Table 10.4.

**Table 10.4:** Autoimmune Diseases and Cardiovascular Risk

Recommendation	Recommendation Class	Level of evidence	References
In the context of preventing cardiovascular events, the benefit of using stricter therapeutic targets specifically due to the presence of autoimmune diseases is uncertain	IIb	C	^513,514,516^

### 10.7. Chronic Kidney Disease

The overall prevalence of CKD is estimated at 11-13%,^517^ and in Brazil, despite inconsistent data, it is estimated that between three and six million people have the disease.^518^ The relationship between CKD and CVD is complex, dynamic and multifactorial. In addition to both sharing risk factors such as systemic arterial hypertension, diabetes and advanced age, there is a higher prevalence of traditional CVD risk factors in patients with CKD.^519,520^ In a study by Foley et al.,^519^ with more than 15,000 patients, 83.6% of those with estimated glomerular filtration rate (eGFR) < 60 ml/min/1.73 m^2^ and 100% of those with GFR-e < 30 ml/min/1.73 m^2^ had at least two risk factors for CVD.^519^ Furthermore, the loss of renal function itself causes changes that can accelerate CVD, such as arterial stiffness and anemia contributing to left ventricular hypertrophy, endothelial dysfunction disease, chronic inflammation, vitamin D deficiency, oxidative stress, and activation of the renin-angiotensin system.^520-523^

The result of this interaction is that CVD is the leading cause of death in patients with CKD.^521^ In a meta-analysis by van der Velde et al.,^524^ evaluating cohorts of patients with hypertension, diabetes or CV disease, they observed an increase in all causes of mortality with eGFR reduction, and rates of 60, 45 and 15 ml/min/1.73m^2^ presented a hazard ratio of 1.03, 1.38 and 3.11, respectively, when compared to patients with eGFR 95 ml/min/1.73 m^2^. In addition, the presence of albuminuria, even when borderline, was also associated with higher mortality in this same study, and urinary albumin-creatinine ratios of 10 mg/g, 30 mg/g and 300 mg/g presented a hazard ratio of 1.08, 1.38 and 2.16 when compared to the ratio of 5 mg/g.^524^

Currently, CKD^525^ and albuminuria are considered independent predictors of CV^522-526^ events and thus, CV prevention plays a key role in the management of CKD patients. Overall, the risk assessment should be individualized and CKD should be interpreted in the context of the overall risk assessment according to each clinical setting, and is considered a high risk CV marker.^7,525^ Given the various clinical scenarios related to CKD, it is worth mentioning systemic arterial hypertension, dyslipidemia and the use of antiplatelet agents in primary prevention.

With regard to systemic arterial hypertension, risk stratification and treatment to prevent events and additional loss of renal function should follow the guidelines published by this Society.^146^ It is noteworthy that in this case, CKD is used in the additional risk stratification according to eGFR and urinary albumin-creatinine ratio, and can be interpreted as target organ damage (eGFR 30-60 ml/min/1.73 m^2^ or urinary albumin-creatinine 30-300 mg/g) or as an established disease (eGFR < 30 ml/min / 1.73 m^2^ or urinary albumin-creatinine > 300 mg/g). Similarly, the approach to dyslipidemia in CKD patients should follow the stratification and treatment model proposed in this Society’s specific guideline.^7^ In this case, CKD (eGFR < 60 ml/min/1.73 m^2^) is considered as a high-risk CV marker for proposed goals and treatments.^7^

Finally, regarding the use of antiplatelet agents in primary prevention, the evidence regarding its benefit is not robust enough to indicate its routine use considering CKD alone. In a meta-analysis of more than 50 studies and more than 27,000 patients, the use of ASA reduced the risk of infarction without, however, reducing overall mortality, CV mortality or stroke, with increased numbers of major and minor bleeds.^527^ Thus, the use of antiplatelet agents should be assessed according to the overall risk and decision-making should be made on an individual basis when considering their use solely for CKD.

Recommendations for CKD and CV risk can be seen in Table 10.5.

**Table 10.5:** Chronic Kidney Disease (CKD) and cardiovascular risk

Recommendation	Recommended class	Level of evidence	Reference
Cardiovascular prevention measures in patients with CKD should be individualized and consider the eGFR, the presence of other associated diseases and the overall cardiovascular risk	I	C	^525-527^

CKD: chronic kidney disease; eGFR: estimated glomerular filtration rate.

### 10.8. Obstructive Sleep Apnea

In recent years, much has been debated about obstructive sleep apnea (OSA) as a CV risk factor and, in 2018, the Brazilian Society of Cardiology published a position on this clinical condition and its implications on CV risk.^528^ OSA is characterized by the temporary narrowing or occlusion of the upper airway during sleep,^529^ which in turn activates the sympathetic nervous system and triggers a chain of events involving elevation of blood pressure, release of inflammatory mediators, oxidative stress, endothelial dysfunction, reduced insulin sensitivity and activation of the renin-angiotensin-aldosterone system.^528-530^ Despite the short duration of events, prolonged repetitive exposure to periods of hypoventilation and hypoxemia can lead to chronic changes in metabolism and circulatory system leading to consequences such as systemic arterial hypertension, pulmonary hypertension, arrhythmias, coronary disease, stroke, heart failure, diabetes, dyslipidemia, and increased mortality CV.^528-533^

The prevalence of OSA has increased in recent years^528,529^ and some series have reported apnea-hypopnea index equal to or greater than 5 events per hour in 34% of men and 17% of women aged 30 to 70.^534^ In CVD patients The prevalence of OSA is higher when compared to patients of the same age and sex in the general population, regardless of body mass index.^529^ Among CVD, hypertension, coronary artery disease, stroke and heart failure with reduced ejection fraction, with reports of associated prevalence of OSA of up to 83%, 58%, 91% and 53%, respectively.^528,529^

The treatment of OSA is mainly based on the use of continuous positive airway pressure (CPAP). There is evidence that this treatment modality has beneficial effects on blood pressure control,^535^ but evidence regarding rigid outcomes such as total and CV mortality is not as robust,^528-530^ with data on primary prevention from observational studies.^531,536^ In a recent systematic and meta-analysis review, no reduction in major CV events including vascular death or all-cause death was observed.^537^ It is worth mentioning that in 60% of the studies evaluated CV disease (secondary prevention) was documented and in such cases, with patients undergoing optimal clinical treatment, CPAP treatment may have little additional effect than current treatment when assessing total mortality and CV outcomes,^530,537^ despite the benefits of blood pressure control and improvement of extra-cardiac symptoms.^530^

Finally, CV prevention strategies in OSA patients should consider the higher morbidity and mortality attributed to this condition, emphasizing the control of other associated risk factors and respecting specific treatment indications according to this Society’s position on group 11 of AOS.^528^

Recommendations for obstructive sleep apnea (OSA) and CV risk are shown in Table 10.6.

**Table 10.6:** Obstructive sleep apnea and cardiovascular risk

Recommendation	Recommended class	Level of evidence	References
Measures for cardiovascular prevention in patients with obstructive sleep apnea should be individualized and consider the presence of other associated diseases, the overall cardiovascular risk and indications for treating the disease itself	I	C	^528,530,537^

### 10.9. Erectile Dysfunction

Erectile dysfunction (ED) is the recurrent inability to obtain and maintain an erection that allows for satisfactory sexual activity. ED is not a disease but a symptomatic manifestation of isolated or associated pathologies.^538^ It has a prevalence of just over 50% in men over 40 years of age in the USA and Brazil. Studies have shown a prevalence between 43 and 46% in the same age range.^538-541^ The causes of ED can be classified as psychological, organic or a combination of both. Organic factors include vascular, endocrine, neurological, drug-related causes, and urological interventions. Vascular etiology is the most common cause of erectile dysfunction. Arterial traumatic disease, atherosclerosis and SAH are among the main causes of vascular ED. Increasing the prevalence in patients with hypertension and / or diabetes and also with aging, it may reach a prevalence of over 68% in these populations and also be related to therapy with CV action drugs that contribute to the occurrence of ED.^542-544^

ED is currently recognized as being of vascular etiology in most men, with endothelial dysfunction as the common denominator. ED often precedes CVD and is often present in men with known CVD, leading to the concept that a man with ED and no CVD symptoms is a patient with CVD until proven otherwise, and a man with known CVD should be routinely asked about your erectile dysfunction. ED also has a significant negative impact on the patient and partner (one man’s problem but a couple’s concern), thus emphasizing the need to approach ED as early as possible.^545^

A meta-analysis of 20 prospective cohort studies involving 36,744 participants suggested that erectile dysfunction significantly increases the risk of ischemic heart disease, stroke and all-cause mortality and concluded that it could play a role in quantifying CV risk based on traditional risk factors.^546^ Another population-based study of 95,038 men aged 45 and over showed that CVD risk is related to the severity of erectile dysfunction in men with and without established CVD, with a relative risk (respectively) of 1.6 and 1.7 for the development of ischemic heart disease.^547^ All men with erectile dysfunction should be considered potential candidates for primary prevention, CV risk stratification and treated according to their risk estimates.

Recommendations for autoimmune diseases and CV risk are listed in Table 10.7.

**Table 10.7:** Autoimmune Diseases and Cardiovascular Risk

Recommendation	Class	Level of evidence	Reference
All men with erectile dysfunction should be submitted to cardiovascular risk stratification and treated according to the observed risk estimate	IIa	C	^9,546,547^

### 10.10. Prevention of Rheumatic Heart Disease

Rheumatic heart disease (RHD) is the cardiac consequence of acute rheumatic fever (ARF), an inflammatory disease caused by streptococcal pharyngitis. Its prevalence is closely related to unfavorable sanitary conditions, agglomerations and inadequate access to health systems.^548^ Over the last decades there has been a significant reduction in prevalence and mortality from RHD worldwide (with a reduction in standardized global mortality of 47, 8% from 1990 to 2015^2^), markedly in developed countries, and even near eradication in some regions. However, the burden of disease remains high in underdeveloped countries and even in poor regions of developed countries.^548^ In 2015, the highest age-standardized mortality rates for RHD prevalence were observed in Oceania, South Asia, and central sub-Saharan Africa, but there is clearly an underestimation of data from Brazil and Latin America, partly due to the scarcity of primary data. It is estimated that in 2015 there were 33.4 million cases and approximately 10.5 million disability-adjusted life years (DALY) attributable to RHD worldwide.^549^

The principal determinant of RF is the admittedly repeated infection with group A beta-hemolytic streptococci (GAS), and some theories attempt to explain the pathophysiology involved in susceptibility to damage, which affects only 6% of individuals exposed to GAS: a) an antigenic similarity between agent structures (M protein surface and GlcNAc epitope) and molecules in host tissues, triggering an exaggerated immune response, and b) generation of a “neo-antigen” through contact between GAS and collagen matrix subendothelial, with consequent binding between M proteins and CB3 region of collagen type IV, inducing an autoimmune response against collagen.^548^

Thus, primary prevention of RF requires early identification and appropriate therapy for GAS pharyngitis. When selecting a treatment regimen, consideration should be given to the bacteriological and clinical efficacy, ease of adherence to the recommended regimen (ie: dosing frequency, duration of therapy and acceptability), cost, spectrum of activity of the selected agent and potential adverse effects. In this context, intramuscular benzathine penicillin G, oral potassium penicillin V and oral amoxicillin are the recommended antimicrobial agents for the treatment of GAS pharyngitis in people without penicillin allergy (Table 10.8). GAS resistance to penicillin has never been documented, and penicillin potentially prevents primary attacks of RF even when started nine days after the onset of infection.^550,551^

**Table 10.8:** Primary and secondary prophylaxis regimens for acute rheumatic fever and rheumatic heart disease

Recommendation	Recommendation level	Level of evidence	Reference
**Primary prophylaxis**			
Penicillins:			
**Amoxicillin** 50 mg/kg (maximum 1 g) VO 1x/day for 10 days **Penicillin G Benzatin** Patients up to 27 kg: 600,000 IU IM in single dose; patients > 27kg: 1,200,000 IU IM in single dose **Penicillin V Potassium** Patients up to 27 kg: 250 mg OR 2 or 3x/day for 10 days; patients > 27 kg: 500 mg OR 2 or 3x/day for 10 days	I	B	^549,550^
Allergic to Penicillin:			
**Low Spectrum Cephalosporins (Cephalexin, Cefadroxil)** Variable **Azithromycin** 12 mg/kg (maximum 500 mg) OR 1x/day for 10 days **Clarithromycin** 15 mg/kg OR per day, divided into 2 doses (maximum 250 mg 2x/day) for 10 days **Clindamycin** 20 mg/kg OR/day (maximum 1.8 g per day) divided into 3 doses for 10 days	IB IIa IIa IIa	B B B B	^549,550^
**Secondary Prophylaxis:**			
**Penicillin G Benzatin** Patients up to 27 kg: 600,000 IU IM every 3 to 4 weeks †; patients > 27 kg: 1,200,000 IU IM every 3 to 4 weeks † **Penicillin V Potassium** 250 mg OR 2x/day **Sulfadiazine** Patients up to 27 kg: 0.5 g OR 1x/day; patients > 27 kg: 1 g OR 1x/day **Macrolide or azalide (for penicillin and sulfadiazine allergic patients) ‡** Variable	1 1 1 1	A B B C	^549,550^

† Administration every 3 weeks is recommended in certain high risk situations.

‡ Macrolide antibiotics should not be prescribed to patients using other cytochrome P450 3A inhibiting drugs such as azole antifungals, human immunodeficiency virus protease inhibitors, and some selective serotonin reuptake inhibitors. IM: intramuscular; IU: international units; OR: orally.

In recent decades, the long asymptomatic period of RHD and the possibility of early interventions in the subclinical phase have led to the increased role of echocardiography in disease management, with the development of population screening studies and the publication of the 2015 revised Jones criteria.^552^ In addition to the incorporation of detected subclinical carditis on echocardiography, patients were stratified according to population risk for RHD,^552-554^ with different criteria for endemic and non-endemic regions (Chart 10.4).

**Chart 10.4:** Summary of Jones Criteria for Acute Rheumatic Fever (2015 Review), highlighting major changes from 1992 review

**Jones criteria revised for diagnosis of ARF^6^**		
ARF Risk	Low risk population: Incidence of ARF ≤ 2 per 100,000 school-age children or prevalence at all ages ≤ 1 per 1000 per year	Moderate to high risk population: Children not included in low risk populations
**Major criteria:**		
*Carditis*	Clinical and/or subclinical*	Clinical and/or subclinical*
*Arthritis*	Polyarthritis	Monoartrite, poliartrite e/ou poliartralgia
	Korea Marked Erythema Subcutaneous nodules	Korea Marked Erythema Subcutaneous nodules
**Minor criteria:**		
Carditis Arthralgia Fever Inflammatory markers	Extended PR Range† Polyarthralgia ≥ 38.5°C ESR peak ≥ 60 mm in 1 hr and / or CRP ≥ 3.0 mg/dL	Extended PR Range† Monoarthralgia ≥ 38°C ESR peak ≥ 30 mm in 1hr and/or CRP ≥ 3.0 mg/dL

Changes from the 1992 revision are highlighted in bold.

* Subclinical carditis: seen only on echocardiography, without auscultatory findings.

† Considering variability by age and only if carditis is NOT counted as a major criterion. ARF: acute rheumatic fever; CRP: C-reactive protein; ESR: erythrocyte sedimentation rate.

Once RHD is diagnosed, prevention strategies should focus on preventing recurrences that are associated with worsening or developing RHD. A GAS infection does not necessarily have to be symptomatic to trigger a recurrence, and RHD can recur even when a symptomatic infection is correctly treated. Therefore, prevention requires continuous antimicrobial prophylaxis rather than simply recognizing and treating acute episodes of pharyngitis.^548^ Therefore, continuous prophylaxis is recommended in patients with well-documented history of RHD and in those with evidence of RHD. Prophylaxis should be started as soon as RHD or RF is diagnosed. In order to eradicate GAS in the oropharynx, a complete penicillin cycle should be given to patients with RHD, even for those with a negative oropharyngeal culture.^548,550,551^

Patients diagnosed with rheumatic carditis with or without valvular disease are at high risk for recurrence and presumably a progressive risk of more severe cardiac involvement at each episode.^555^ These patients should receive long-term antibiotic prophylaxis until adulthood and, in selected cases, for life. Patients with persistent valvular disease should receive prophylaxis for 10 years after the last episode of RHD or until the age of 40, whichever one is longer. The severity of valvular disease and the potential for day-to-day GAS exposure should be determined, and lifetime prophylaxis should be considered in those at high risk (e.g, permanent contact with children in schools and day care centers, care for institutionalized patients, work in health facilities etc.).^550,551^ In non-endemic regions, administration of benzathine penicillin G every 4 weeks is the recommended regimen for secondary prophylaxis in most situations. In higher-risk populations, administration every 3 weeks is warranted because serum antimicrobial levels may fall below protection levels before 4 weeks after the initial dose (Table 10.9).

**Table 10.9:** Duration of secondary prophylaxis regimens for acute rheumatic fever and rheumatic heart disease

Type	Duration after last episode	Class	Level of evidence	Reference
ARF with carditis and residual heart disease (persistent valve disease)^†^	10 years or up to 40 years old (whichever is longer); Lifelong prophylaxis may be required	I	C	^549,550^
ARF with carditis but no residual heart disease (absence of persistent valve disease)^†^	10 years or up to 21 years old (whichever is longer)	I	C	^549,550^
FRA without carditis	5 years or up to 21 years old (whichever is longer)	I	C	^549,550^

ARF: acute rheumatic fever.

† Clinical or echocardiographic evidence.

Regarding echocardiographic screening studies in high-risk populations have shown that its accuracy is arguably higher than auscultation for detection of subclinical RF,^554^ and its application at the research level has grown exponentially in the last decade. Based on screening programs involving more than 100,000 patients, in 2012, the World Heart Federation (WHF) published the first evidence-based consensus standardizing the criteria for echocardiographic diagnosis of RF (borderline and definitive).^555^ The concepts of subclinical (echocardiographic findings without alterations on clinical examination) and latent (a broader spectrum encompassing RHD present on echocardiography, with no known prior history of RF or RHD) were defined.^555^

The population echocardiographic screening strategy has already been tested in Brazil, and its implementation has proved feasible in schools - especially the public schools in regions with low socioeconomic indices - and primary health care, with diagnostic support by telemedicine.^556,557^ In addition, non-physician imaging using the WHF simplified protocol was effective, including the basic identification of changes related to RHD.^558^ There was a high prevalence of subclinical RHD in low-income regions of 4.5% (4.0% borderline and 0.5% definitive).^556,557^

However, despite the various cohorts involving these patients, the clinical significance and prognostic implication of these findings has not been well established so far. Recently, a score derived from large population studies in Brazil and Uganda has been proposed to stratify patients according to the risk of RHD progression, based on weights attributed to the echocardiographic variables in the WHF criteria.^559^ However, it has also been shown that giving a child a diagnosis of latent RHD can potentially worsen their quality of life and create stigmas,^560^ which raises important questions about the risk-benefit ratio of large screening programs. For these reasons, there is no indication for the use of echocardiographic screening outside the research field until further studies on its impact on disease progression are completed.

## 11. Child and Adolescence

### 11.1. Introduction

Childhood and adolescence are the phases with the most potential for the prevention of atherosclerosis. There is robust evidence, based on analyzes of the aortas and coronary arteries, that atherosclerosis begins at fetal age. However, more recent studies show that atherosclerosis may regress in children more easily than in adults, since their lesions are less complex and fixed. CVD risk factors respect the tracking phenomenon, i.e., a child who has some risk factor will probably have the same factor in adulthood, with similar intensity. Coupled with the fact that health habits are formed in childhood and adolescence, there is a clear need and possibility to prevent atherosclerosis from an early age.^561^ Therefore, we will present strategies to control the main habits and risk factors that can be controlled in this age group. (Recommendation Level IIa; level of evidence B).

### 11.2. Childhood and Adolescent Nutrition

Nutrition is the basis of health promotion in childhood and adolescence. In addition, eating habits are mainly formed by 7 years of age, reinforcing the importance of food education from an early age. Population studies show that almost all children ingest larger amounts of poor quality fat and added sugar or lower amounts of fiber than recommended for their age. The following principles are recommended for good child growth and development to prevent atherosclerosis from childhood:^562-564^ (Recommendation Level IIa; level of evidence C).

1. Exclusive breast milk up to 6 months, and introducing other foods up to 2 years old.
2. Eating fresh and whole foods from 6 months of age, starting with pureed foods and then eating the family diet, which should be as healthy as possible.
3. Age-appropriate caloric intake, taking into account their basal metabolic rate, as well as growth and exercise needs, except in children with special conditions, or inadequate growth and body composition.
4. Offer the child the most varied and colorful food possible, respecting the proportionality between protein (10 to 20% of total daily caloric volume), fat (30 to 40%) and carbohydrates (30 to 50%) in each age, provided there are not any risk factors that require different proportions.
5. Encourage the daily intake of fruits and vegetables by offering this type of food at every meal. The child should eat the equivalent of his age + 5 in grams of fiber.
6. Avoid sugar (ideally less than 5% of total daily calories), coffee, canned goods,fried foods, soft drinks, candies, snacks and other treats throughout development, these foods should be banned in infants. Replace, whenever possible, processed and ultra-processed foods with fresh or minimally processed foods, regardless of age and body composition.
7. Use salt sparingly. Children’s food must have a less spice and salt than an adult’s; 1.2 to 1.5 g/day of salt for children up to preschool age and up to 2 g/day in school children and adolescents.
8. Associate proteins of animal and vegetal origin, eat whole grains and vegetables at least 5 times a week, in the ratio of 3:1. Animal proteins should be of varied origins, encouraging the consumption of fish.
9. Frequent water intake throughout the day, limiting the intake of juices, even if natural and without added sugar: Ideally, only provide juices from 1 year of age, and at most 120 mL, 180 mL and 240 mL, for infants, preschoolers and schoolchildren, respectively.
10. Offer high nutritional value fats, such as nuts (nuts, almonds, walnuts, among others) and vegetable oils, as long as they are safe (avoid fresh nuts in children under 3 years due to the risk of aspiration) and according to age-appropriate amount. Avoid the intake of trans fats as much as possible.

For children with dyslipidemia, fat intake should be limited to about 25-30% of their total daily calories, while maintaining a proportion of < 7 to 10% saturated fat and 20% mono and polyunsaturated fat, similar to recommendations for adults. The addition of sugar should be avoided and the intake of omega-3 in the form of fish rich in these fatty acids ideally 2 or 3 times a week should be encouraged. Follow-up with a nutritionist or nutrologist is recommended when there is a risk of malnutrition or impaired growth and development.^565^ (Recommendation Level IIa, evidence level A).

For children with SAH, the DASH diet should be used, as in adults, which includes increasing the proportion of fresh foods, especially fruits and vegetables, and reducing salt intake.^566^ (Recommendation Level IIa, level of evidence B).

Control of the food environment is of utmost importance in childhood and adolescence, especially the school environment, which should be protected by public policies that encourage the supply of foods with high nutritional value and restrict ultra-processed, high-calorie or high-density foods with added sugar and trans fats.^567^ (Recommendation Level I, level of evidence C).

### 11.3. Physical Activity in Childhood and Adolescence

Physical activity is considered an independent protective factor in the primary prevention of coronary artery disease since childhood, because of its effect on modulating traditional risk factors and promoting normal endothelial function. Higher levels of physical activity are associated with improved bone health, nutritional status, cardiometabolic health, cognitive function, and reduced risk of depression.^568^ Intervention programs to increase physical activity in children are associated with improved blood pressure and lipid profile.^569^

Physical activity is considered any body movement that results in energy expenditure. Physical exercise consists of planned, structured and repetitive physical activity.

In Brazil, the prevalence of physical inactivity was assessed in a sample of 74,589 adolescents in the Study of Cardiovascular Risks in Adolescents (ERICA). The prevalence of leisure-time physical inactivity reached 54.3%, being especially worrying in female adolescents (70.7%). More than a quarter of adolescents reported no leisure-time physical activity.^570^

The discussion about childhood physical activity has two important aspects for cardiovascular prevention. The first is the tracking phenomenon described above, highlighting the importance of establishing healthy habits at a time when the child is developing, which is much easier to intervene than after the sedentary lifestyle has been established and excessive screen time (more than 2 hours/day). The second aspect is the accumulation of risk or protective factors over the course of life, which can determine different levels of risk over many years of exposure.

Regarding these concepts, in 2016, the American Heart Association published the Cardiovascular Health Promotion in Children document: Challenges and Opportunities for 2020 and Beyond The Scientific Statement From the American Heart Association, stating that maintaining optimal CV health from birth to young adulthood is critical part of reducing CVD disease in adulthood.^571^

The physical activity level considered ideal for children and adolescents aged 6 to 17 years is 60 minutes or more per day of intense to vigorous aerobic activity. The document also recommends performing muscle strength activity and muscle-strengthening and bone-loading1 at least three times a week (Recommendation Level IIa, Evidence Level B).^568,571^

Preschoolers (3-5 years old) should remain active throughout the day to encourage growth, development and to acquire a repertoire of motor skills. Caregivers should aim to achieve a total of at least 3 active hours per day, diversifying from mild to vigorous intensities (Recommendation Level IIa, level of evidence B).

Although there is no consensus on the amount of activity or exercise needed to treat CV risk factors such as dyslipidemia, hypertension or obesity in childhood, it is known that even without effective control of their CV risk, physical activity is one of the most important pillars in the prevention of atherosclerosis, with improvement of endothelial function and even regression of intimal thickening, markers of subclinical atherosclerosis.^571,572^ (Recommendation Level IIa, level of evidence B).

Current evidence for adults shows that total activity volume is more important than the duration of each individual session.^573^

Recommendations for all age groups emphasize increasing overall physical activity (moving more) and reducing sedentary activity (avoiding long sitting periods) whenever possible. For children, this means encouraging outdoor play whenever possible, activities with different levels of intensity, such as walking the dog, storing toys, walking to school, among others. It also means, from a public policy point of view, ensuring safe spaces for children and adolescents to play sports or jogging, an urban layout that encourages walking or cycling, and the structure and availability of qualified physical exercise teachers, schools and other community locations such as parks and gyms.^568,573^ (Recommendation Level I, level of evidence C).

### 11.4. Smoking in children and Adolescence

About 18.5% of Brazilian adolescents have tried cigarette smoking. Smoking increases CV risk in childhood, even when it is passive: low birth weight, higher risk of childhood obesity; it also determines endothelial dysfunction as early as childhood, in addition to all pulmonary neurological risks.^574,575^ Childhood is the most important phase for smoking prevention, as about 90% of people start smoking by age 18. In addition, it is an ideal moment for parental smoking cessation, as they may change their habits if the harmful effects of secondhand smoke are shown to their children. This intervention should occur in different environments, 2 of which may be directly addressed by the physician:^576^ (Recommendation level I, level of evidence C).

At the pediatrician clinic:

1. Ask about your child’s passive exposure to smoking during childcare consultations and in consultations regarding potentially smoking-related illnesses. Ask about caregiver and environment smoking, electronic cigarettes and cannabis use.
2. Include smoking prevention in your childcare agenda. Clarification about the harms of smoking in consultations from 5 years of age. For teens, talk about the effects on appearance, sports performance and costs. Discuss electronic cigarette.
3. Recommend treatment for caregivers who smoke. Refer to specialized smoking cessation services.
4. Offer treatment to adolescent smokers users who want to quit smoking. Moderate or severe adolescent users may benefit from drug treatment. Periodic follow-up should occur due to the high chance of relapse.
5. Closely assess the risk of psychiatric symptoms during treatment. Suicidal ideation and suicide may occur, which must be monitored and treated.
6. Do not recommend the use of electronic cigarettes. The harmful effects are similar.
7. If second-hand smoke cannot be eliminated, agree on measures that minimize exposure.

**In medical schools:**

At all levels of teaching and learning and for all health professionals, smoking cessation training should be provided. The prevention of active and passive smoking, as well as forms of intervention in smoking cessation should be part of the curriculum of pediatric and family medicine residency programs, due to the great importance of abuse in the general population. (Recommendation Level I, level of evidence C).

### 11.5. Obesity in Childhood and Adolescence

Between 1975 and 2016 the prevalence of obesity between 5 and 19 years increased on average from 0.7% to 5.6% in girls and from 0.8% to 7.8% in boys in all geographic regions of the world. The study estimated that in 2016 there were 50 million obese girls and 74 million obese boys worldwide.^577^ In Brazil, the 2015 National School Health Survey identified a prevalence of overweight and obesity in 23.3 % and 8.5% in students from 13 to 17 years old, respectively.^578^

#### 11.5.1. Diagnosis

BMI is used as the standard measure of overweight and obesity in children from two years of age,^579^ using World Health Organization reference curves. (https://www.who.int/childgrowth/standards/bmi_for_age/en/). Overweight is defined as between the 85^th^ and 94^th^ BMI percentile; obesity above the 95^th^ percentile; severe obesity, when BMI is greater than or equal to 120% of the 95th percentile or BMI equal to or above 35 kg/m^2^. (Recommendation Level IIa, level of evidence C).

#### 11.5.2. Consequences

Childhood obesity is associated with dyslipidemia (high triglyceride levels and low HDL-cholesterol), hypertension, hyperglycemia, hyperinsulinemia, inflammation and oxidative stress, favoring the evolution of fatty striae in the aorta and coronary arteries, as well as other atherosclerotic lesions.^580^

About 50% of obese children aged 6 years and one obese parent will have obesity in adulthood; 80% of obese adolescents in this condition, will be an obese adult.^580^

#### 11.5.3. Etiology

It is the result of the interaction between genetic factors and environmental factors; sedentary lifestyle and excessive calorie consumption, the focus of treatment strategies, are among the latter.^580,581^ The secondary causes of childhood obesity are described in Chart 11.1.

**Chart 11.1:** Causes of secondary obesity in childhood and adolescence

Cause Type	Examples
Medicines	Psychoactive drugs (olanzapine, risperidone), antiepileptic drugs, corticosteroids
Endocrine Diseases	Cortisol excess, hypothyroidism, growth hormone deficiency, pseudohypoparathyroidism, hypothalamic obesity
Genetic syndromes	Prader-Willi, Bardet-Biedl, melanocortin or leptin receptor mutation
*Programming*	Epigenetic changes in vulnerable phases of pregnancy and childhood
Other	Intestinal microbiome, individual response to viruses and toxins

#### 11.5.4. Treatment

The therapeutic approach for overweight in children and adolescents should be multiple and gradual, with progressive evaluation of the results obtained and involve better diet quality, reduced calorie intake, increased physical activity and meal replacements. Pharmacotherapy (Orlistat is currently the only one approved for use in adolescents) and bariatric surgery has only been used in severely obese adolescents when dietary and physical activity strategies are not effective in weight control.^580,581^ (Recommendation Level IIa, level of evidence B).

### 11.6. Systemic Arterial Hypertension in Childhood and Adolescence

BP screening data in childhood and adolescence show a prevalence of SAH of up to 8.2%,^582,583^ which decreases to approximately 3.5% when measurements are repeated at clinical follow-up. Prehypertension is observed in approximately 2.2 to 3.5% of the population; in overweight and obese adolescents, it can reach 24.8%. It is also associated with sleep disorders (3.6 to 14%), chronic kidney disease (up to 50%), diabetes mellitus (9.5%); aorta narrowing (17 to 77%), endocrine alterations (0.05 to 6%) and prematurity 7.3%.^584^ Although hypertension in children is more often due to a secondary cause, with a defined etiology, there has been an increase in the diagnoses of primary hypertension, especially in older children and adolescents, when other risk factors are associated, such as overweight and obesity. Blood pressure measurement is considered mandatory from the age of three, on an annual basis, or before this age when the child has a neonatal history, history of prematurity, history of aortic narrowing, kidney disease, diabetes mellitus or is using medication that can increase blood pressure. SAH is defined by the blood pressure percentile in relation to age, sex and height. The tables with gender, age and height percentiles (https://pediatrics.aappublications.org/content/pediatrics/140/3/e2017) have been redefined in the American Clinical Practice Guideline for Screening and Management of High Blood Pressure in Children and Adolescents, facilitating the adoption of a single table containing the three parameters used and the assigned percentile. As we do not have specific tables for the Brazilian population, this criterion is used for our population. The first blood pressure measurement can be performed by the oscillometric method on the right arm using an appropriate cuff. If the result of this measurement is greater than or equal to the 90th percentile, another measurement must be taken; If the mean of these two measurements is still ≥ 90^th^ percentile, two measurements by auscultatory method should be performed. Table 11.1 shows blood pressure levels in normal and hypertensive children and adolescents. (Recommendation Level I; level of evidence B).

**Table 11.1:** Blood pressure classification in children and adolescents^563^

Up to 13 years old	Systolic or diastolic blood pressure percentile
Normal (1-13 years old)	< 90
High blood pressure	≥ 90 to <95 or PA 120 x 80 mmHg at < 95 (whichever is lower)
SAH stage 1	≥ 95 to < 95 + 12 mmHg or 130 x 80 mmHg to 139 x 89 mmHg (whichever is lower)
SAH stage 2	≥ 95 + 12 mmHg or ≥ 140 x 90 mmHg (whichever is lower)

In children and adolescents > 13 years of age, blood pressure is considered normal when: < 120/80 mmHg; elevated when between 120/< 80 and 129/< 80 mmHg, HAS stage 1 when between 130/80 and 139/89 mmHg and stage 2 when ≥ 140/90 mmHg. (Recommendation Level I; level of evidence B).

When BP remains persistently at or above the 90th percentile, measured 6 and 12 months after initial diagnosis, the initial assessment should attempt to identify the etiology, if any, based on information on sleep habits, family history, and risk factors, diet, smoking and alcohol intake. It is important that BP is measured in both upper limbs and one lower limb. The initial complementary exams should include: blood count, urea dosage, creatinine, sodium, potassium, calcium, uric acid, lipid profile, urine summary, renal ultrasound when < 6 years of age or with impaired renal function. For children with a BMI percentile greater than the 95th percentile, glycosylated hemoglobin, liver enzymes, blood glucose and fasting lipid profile should also be ordered.^584,585^ (Recommendation Level IIa; level of evidence C).

When BP indicates stage 1 or 2 hypertension in asymptomatic children, it should be confirmed by three measurements and ABPM. Non-pharmacological measures should be initiated and, only if necessary, drug treatment should be started. If the child is symptomatic or the BP is 30 mm Hg above the 95th percentile or > 180 x 120 mmHg in adolescents, the patient should be referred to an emergency room service.^584^^.^^585^ (Recommendation level IIa; Level of Evidence B).

ABPM is indicated in children above 5 years of age when the diagnosis of elevated BP continues after one year from the initial diagnosis or after three measurements in patients with stage 1 hypertension, it is very important to investigate white coat and masked hypertension as well as for diagnosis in obese patients. Additional tests are needed when there is a suspected disease with elevated BP, these include: polysomnography, renin dosage or plasma renin activity; renal scintigraphy with captopril administration; dosage of plasma and urinary catecholamines; dosage of steroids in plasma and urine; nuclear magnetic resonance; digital angiography and renal arteriography. Echocardiography should be performed when drug treatment is indicated for target organ injury evaluation.^586^ (Recommendation level IIa; Level of Evidence B).

The drug treatment for hypertension in childhood and adolescence is similar to that of adults. Due to the ease of supply in SUS in Brazil, the most used drugs among these groups are described in Chart 11.2. Treatment should be initiated on its own with one of the above drugs and when necessary a second drug, with hydrochlorothiazide bring the prefered choice.^586^ (Recommendation Level I; Level of Evidence B).

**Chart 11.2:** Antihypertensive drugs most frequently used in the treatment of hypertension in children and adolescents in Brazil

Medicine	Dose
Captopril	0,5-6 mg/kg/day
Enalapril	0,08-0,6 mg/kg/day
Losartan (> 6 years old)	0,7-1,4 mg/kg/day (max 100 mg/day)
Amlodipine (1-5 years old) (> 6 years old)	0,1-0,6 mg/kg/dia (max 5 mg/day) 2,5-10 mg/day
Hydrochlorothiazide	1-2 mg/kg/day (max 37,5 mg/day)

### 11.7. Dyslipidemia in Childhood and Adolescence

Dyslipidemia is known to be one of the CV risk factors with the greatest impact on accelerating the progression of atherosclerosis. Considering all lipid fractions, the prevalence of dyslipidemia in childhood and adolescence has remained between 30-40%.^587^ According to the ERICA study, which evaluated 38,000 adolescents in Brazil, the prevalence of dyslipidemia in this group was as follows: 46% had HDL-cholesterol concentrations below 45 mg/dL, 20.1% had total cholesterol concentrations greater than 170 mg/dL, 7.8% had triglyceride concentrations greater than 130 mg/dL, and 3.5% LDL-cholesterol concentrations greater than 130 mg/dL.^588^

#### 11.7.1. Causes

The causes of primary or secondary dyslipidemia are similar in adults and children. It is worth mentioning some specificities in childhood, such as the higher prevalence of more severe primary types that do not allow survival until adulthood if not treated intensively and early, such as familial hypercholesterolaemia (heterozygous or homozygous), and lipoprotein lipase deficiency (monogenic hypertriglyceridemia). Among the secondary causes, ketogenic diet, used in refractory epilepsy, has been identified more frequently, in addition to obesity, physical inactivity and inadequate diet, considered at epidemic levels in the country.^589^

#### 11.7.2. Normal Values

The lipid profile should be measured between 9 and 11 years of age. At the population level, fasting-free dosing can be of great value, due to its practicality and cost, especially in these cases measuring HDL and non-HDL levels. In younger children, it should be done in children 2 years and older when there is an early family history of atherosclerosis, any CV risk factor or habits (Table 11.2) or clinical signs compatible with monogenic severe primary dyslipidemia. Normal values are described in Table 11.3.^590,591^

**Table 11.2:** Cardiovascular diseases and risk factors, according to risk intensity, in children and adolescents

Type and intensity of injuries	Health problems
High risk diseases	*Diabetes mellitus, renal failure, heart or kidney transplantation, Kawasaki disease with aneurysm*
Moderate risk diseases	Chronic inflammatory diseases, HIV infection, Early coronary insufficiency in the family
High risk factors	Blood pressure above the 99th percentile medicated, smoking, body mass index above the 97th percentile
Moderate risk factors	Hypertension without indication for drug treatment, obesity between 95 and 97 percentile, HDL < 40 mg/dL

**Table 11.3:** Reference values for lipids and lipoproteins in children and adolescents

Lipids	Fasting (mg/dL)	Not fasting (mg/dL)
Total cholesterol	< 170	< 170
HDL-cholesterol	> 45	> 45
Triglycerides (0-9 years old) (10-19 years old)	< 75 < 90	< 85 < 100
LDL-cholesterol	< 110	< 110
Non-HDL-cholesterol	> 145	> 145

Adapted from "Expert panel on integrated guidelines for cardiovascular health and risk reduction in children and adolescents: summary report".

#### 11.7.3. Treatment

The treatment is initially based on intensive lifestyle modification for at least 6 months, with weight control, diet and physical activity, as already described.^7^

The goal of LDL-cholesterol for drug use varies according to the risk profile of the child or adolescent following unsuccessful lifestyle changes (Table 11.4). The drug arsenal is similar to that of adults by age group as described in Table 11.5.^7,592^

**Table 11.4:** LDL-cholesterol targets in children and adolescents, according to cardiovascular risk profile

LDL-cholesterol levels	Risk
< 190 mg/dL	Without another risk factor
< 160 mg/dL	Early coronary insufficiency in the family Or other risk factor
< 130 mg/dL	Established coronary insufficiency OR 2 diseases or high risk factors OR 1 disease or high risk factor AND 2 diseases or moderate risk factors (Table 11.3)

**Table 11.5:** Medicines used to treat dyslipidemia in childhood and adolescence

Medicine	Dose	Observations
Lovastatin, pravastatin, simvastatin and atorvastatin	10-40 mg/day	Pravastatin for HIV and atorvastatin for HF (> 7years)
Rosuvastatin	5-20 mg/day	Mainly in HF (> 7 years)
Cholestyramine	4-16 g/day	Any age
Ezetimibe	10 mg/day	> 4 years old
Bezafibrate, fenofibrate	200-600 mg/day	TG persistently > 500 mg/dL
Omega 3	2-4 g/day	Variable effect
Phytosterols	1,2-1,5 g/day	Variable effect

There is no robust evidence on the use of medications in cases of hypertriglyceridemia. However, those of the fibrate class can be used in children older than 12 years, similarly to adults, when triglyceride levels reach concentrations of 700 mg/dL or persistently above 500 mg/dL even with all conventional control measures.^593^ Table 11.6 shows the recommendations for approaching children and adolescents.

**Table 11.6:** Recommendations for approaching children and adolescents

Recommendation			Recommendation class	Level of evidence	Reference
Blood pressure classification in children and adolescents					
**Up to 13 years old**	**Systolic or diastolic blood pressure percentile**				
Normal (1-13 years old)	< 90				
High blood pressure	≥ 90 to < 95 or PA 120 x 80 mmHg at < 95 (whichever is lower)		I	B	^590-593^
SAH stage 1	≥ 95 to < 95 + 12 mmHg or 130 x 80 mmHg to 139 x 89 mmHg (whichever is lower)				
SAH stage 2	≥ 95 + 12 mmHg or ≥ 140 x 90 mmHg (whichever is lower)				
Antihypertensives most frequently used in the treatment of hypertension in children and adolescents in Brazil: captopril, enalapril, hydrochlorothizide, amlodipine and losartan in children over 6 years old			I	A	^590-593^
Reference values for lipids and lipoproteins in children and adolescents					
**Lipids**	**Fasting (mg/dL)**	**Not fasting (mg/dL)**			
Total cholesterol	< 170	< 170			
HDL-cholesterol	> 45	> 45			
Triglycerides (0-9 years old) (10-19 years old)	< 75 < 90	< 85 < 100	I!a	C	^590-593^
LDL-cholesterol	< 110	< 110			
Non-HDL-cholesterol	> 145	> 145			
LDL-cholesterol targets in children and adolescents according to cardiovascular risk profile					
**LDL-cholesterol levels**	**Risk**				
< 190 mg/dL	without another risk factor		IIa	C	^590-593^
< 160 mg/dL	Early coronary insufficiency in the family Or other risk factor				
< 130 mg/dL	Established coronary insufficiency OR 2 diseases or high risk factors OR 1 disease or high risk factor AND 2 diseases or moderate risk factors (Table 11.3)				
Drugs used to treat dyslipidemia in childhood and adolescence					
**Medicine**	**Dose**	**Observations**			
Lovastatin, pravastatin, simvastatin and atorvastatin	10-40 mg/day	Pravastatin for HIV and atorvastatin for HF (> 7years)			
Rosuvastatin	5-20 mg/day	Mainly in HF (> 7 years)	IIa	A	^590-593^
Cholestyramine	4-16 g/day	Any age			
Ezetimibe	10 mg/day	> 4 years old			
Bezafibrate, fenofibrate	200-600 mg/day	TG persistently > 500 mg/dL			
Omega 3	2-4 g/day	Variable effect			
Phytosterols	1,2-1,5 g/day	Variable effect			

LDL-a: low-density lipoprotein cholesterol; SAH: systemic arterial hypertension.

## 12. Populational Approach to Risk Factors for Cardiovascular Diseases

### 12.1. Introduction

The population is aging, in Brazil and in the world. The Brazilian population has maintained an aging trend in recent years and has gained 4.8 million elderly people since 2012, surpassing the 30.2 million mark in 2017, according to the National Household Sample Survey - PNAD.^594^

In 2012, there were 25.4 million people aged 60 and over. The 4.8 million new elderly in five years correspond to an 18% growth in this age group, which has become increasingly representative in Brazil. Women are the majority in this group, with 16.9 million (56% of the elderly), while elderly men are 13.3 million (44% of the group).^594^

**Figure 12.1:**
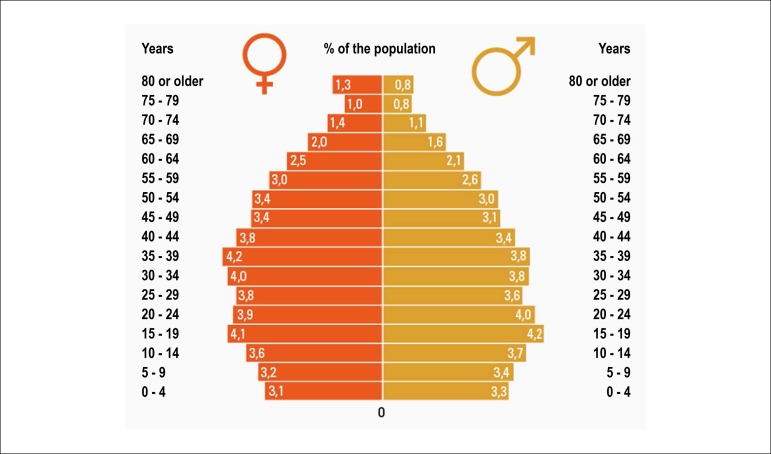
Population distribution by sex and age group - 2017. Source: Number of elderly grows 18% in 5 years and exceeds 30 million in 2017. IBGE.^1^ https://agenciadenoticias.ibge.gov.br/agencia-noticias/2012-agencia-de-noticias/noticias/20980-numero-de-idosos-cresce-18-em-5-anos-e-ultrapassa-30-milhoesem-2017.html

Between 2012 and 2017, the number of elderly grew in all units of the federation, with Rio de Janeiro and Rio Grande do Sul being the states with the highest proportion of elderly, both with 18.6% of their populations within this age group. Amapá, in turn, is the state with the lowest percentage of the elderly, with only 7.2% of the population (Figure 12.1).^594^

According to the WHO, the world’s population of elderly people is increasing, and in the coming decades the world’s population of people over 60 years of age will grow from the current 841 million to 2 billion by 2050, making chronic diseases and well-being new global public health challenges.^595^

“By 2020 we will have for the first time in history more people over 60 than children under five,” reported the WHO in a health and aging series in The Lancet medical journal, noting that 80% of older people will live in low- and middle-income countries.^595^

The WHO also states that the increase in longevity, especially in high-income countries, is mainly due to the decline in CVD deaths - such as stroke and ischemic heart disease, through simple and cost-effective interventions to reduce smoking and high BP.^595^

**Figure 12.2:**
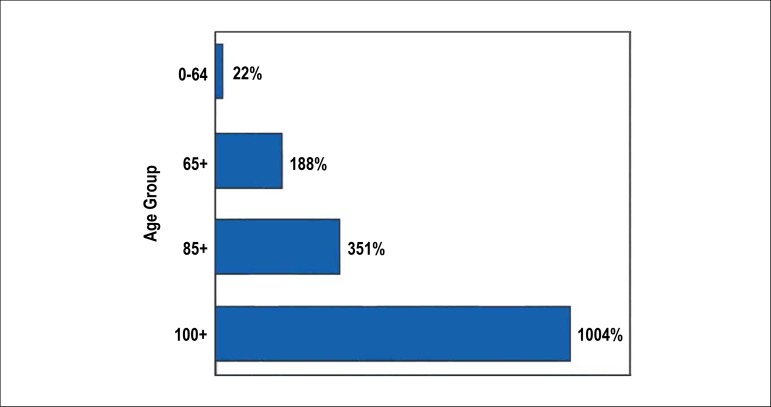
Percentage of change in world population according to age: 2010-2050.596 Adapted from United Nations, World Population Prospects: The 2010 Revision.

Old people or very old people (aged 85 and over) will increase by 351% between 2010 and 2050, compared with an increase of 188% for the population aged 65 and over and an increase of 22% for the 65-year-old population (Figure 12.2).^596^

Over the next 10 to 15 years, people in every region of the world will suffer more deaths and disabilities from noncommunicable diseases such as heart disease, cancer, and diabetes.^596^

These data are directly linked to inadequate lifestyles of the population, such as physical inactivity, obesity and stress, leading to an increased prevalence of risk factors such as hypertension, smoking, diabetes and dyslipidemia, with consequent increase in mortality and CV morbidity.

**Figure 12.3:**
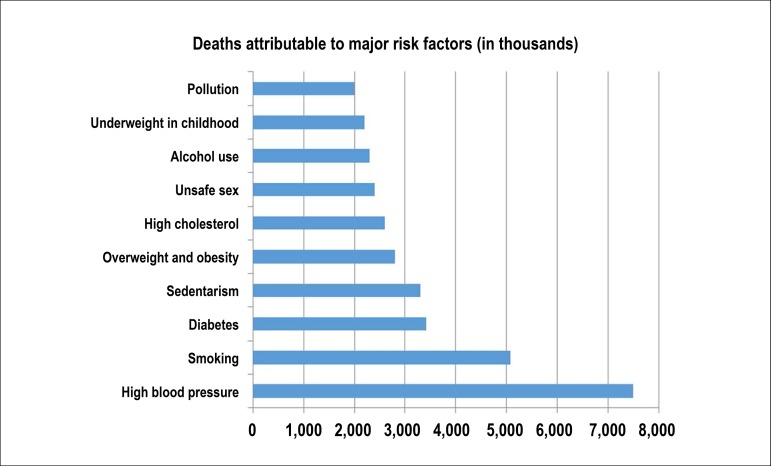
Deaths attributable to major risk factors.^597-599^

AH is the leading risk factor for death and CVD worldwide^597,598^ Figure 12.3.^597-599^ The deaths attributable to CV risk factors can be seen in Figure 12.3.

Based on the above data, strategies to address these risk factors, as well as disease prevention and health promotion actions for at-risk populations should be implemented as soon as possible, and start as early as possible.

Some considerations related to the population aspects of risk factors will be discussed specifically in this chapter and others.

### 12.2. Population Aspect of Smoking

Cigarette smoking is one of the leading and preventable causes of mortality in the world. This habit accounts for 12% of adult mortality worldwide, which corresponds to 5 million people; if this persists, 10 million people per year will die and 70% of these deaths will occur in developing countries.

Specifically in Brazil, the National Congress approved the text of the Global Framework Convention on Tobacco Use (FCTC) through Legislative Decree 1012 of October 28, 2005, and the Brazilian government ratified the 2005 Convention which entered into force on February 1, 2006.

The primary goal of the FCTC is to preserve present and future generations from the devastating health, social, environmental and economic consequences of smoking and exposure to tobacco smoke. It establishes some of its obligations to draft and update smoking control policy, establish an international coordination and cooperation mechanism with other States Parties, and protect national policies against the interests of the tobacco industry.

World No Tobacco Day was created in 1987 by the member states of the World Health Organization to draw the world’s attention to the epidemic of smoking and preventable tobacco-related diseases and deaths; there are over 1 billion smokers in the world and 80% of them are in low- and middle-income countries where the effects of smoking-related illnesses and deaths is highest; current smokers are presumed to consume around 6 trillion cigarettes every year.

### 12.3. May 31^st^ - World No Tobacco Day

Federal Law 12546/2011 in force since December 2014 was published and must be known and respected by all, as well as be adequately enforced the Health Surveillance Sectors in particular.^601^ Data on VIGITEL released in April 2012 revealed a 14.8% drop in smokers in Brazil in people over 18 years.

Among men, the percentage of smokers was 18.1% and in women 12%. The capitals with the most smokers are: Porto Alegre 23%, Curitiba 20% and São Paulo 19%; and Maceió 8%, João Pessoa, Aracaju and Salvador are the capitals with the lowest incidence of smoking in the Northeast with 9%.^602^

There are strategies for tobacco control:

**a) Prevention**

It is essential to prevent young people from trying cigarettes, because if they do, there is a 50% chance that they will become addicted.

Hence the importance of education from family and schools.

Enforcement of the Anti-smoking Act which bans the advertisement of tobacco products and other actions directed at underage young people.^603^

**b) Protection**

Protect the population from the effects of environmental tobacco smoke and the influences that lead to smoking, particularly those related to the social group.

Strictly enforce the anti-smoking law, which among other rules, prohibited smoking in public environments.^603^

### 12.4. Population Aspects of Obesity and Overweight

Relationships between sociodemographic characteristics and lifestyle habits such as income, socioeconomic status, nutritional status, and physical inactivity with weight gain have been established.^604-610^ In addition, especially in the last two decades, international authorities have strongly recommended the implementation of effective obesity prevention policies. However, no country in the world has succeeded in reversing the obesity epidemic.^611,612^

A number of factors may explain the failure to combat obesity, but perhaps the most important is the way it is still understood by most people. Instead of being perceived as a chronic, complex disease, the result of the interaction of genetic and environmental variables, strongly influenced by socio-economic and cultural factors with a highly obesogenic environment, obesity is seen as a personal failure. Obese people are often blamed for their disease, being judged lazy, undisciplined, unmotivated and negligent.^611,612^

From a population standpoint several measures have already been tested and found to be successful locally or for a predetermined period of time. The great challenge is to institute these measures in a more comprehensive and lasting way, and to identify cultural and regional particularities that allow adaptations of these policies to each of the realities.^613,614^

Interventions in schools are the most common and most promising, precisely because of their educational and anti-obesity character in the early stages of life. Modifications in school lunches, sedentary lifestyle, health education are examples of measures that have been beneficial not only for the children and adolescents involved, but also for adults in the same circle.

Fighting physical inactivity in an organized way, with mass campaigns, in addition to more regionalized actions, focused on a particular exercise practice has also been shown to be beneficial for reducing obesity. There is also a lot of research focused on reducing the amount of physical activity required to achieve the weight reduction benefit. Such studies arise precisely from the lack of time to devote to exercise, which ends up being the justification for the sedentary lifestyle of most people.^615^ Finally, there are still population actions aimed at improving people’s diets. Such measures are extremely varied, but ultimately use mostly financial interventions to target dietary choices and habits that are associated with overweight/obesity. There are examples of taxing sweetened beverages, financial incentives to purchase healthy foods, types of financial penalties for buying unhealthy foods, reductions in health-care-related health insurance costs, and maintaining healthy diets.^618^

A concern that should be highlighted in view of population aging is the high prevalence of obesity in older populations. Such individuals should be evaluated very carefully, since the identification of obesity is not so simple, but mainly because of its association with musculoskeletal diseases, diabetes and AMI.^619^

### 12.5. Population Aspects of Hypertension

The treatment of hypertension is known to be effective in relation to the individual, but from the population point of view, it has been frustrating for many reasons.^620-624^ These begin with the lack of education in general and particularly in health, which prevents knowledge about the disease and its importance as one of the main CV risk factors.^594-599^ They go through the difficulty of accessing health services for the correct diagnosis, proper treatment with guidance on lifestyle and medication use and end with the great challenge of treatment compliance.^620-624^ These assumptions alone are sufficient to definitively indicate the actions that can effectively modify the natural history of hypertension and interfere with the equation CV risk, morbidity and mortality. These actions, at the collective level and focusing on primary and primordial prevention, will have a great interface with other CVD risk factors.^10,620-626^

Primordial (prevention of risk factors) and primary (actions on installed risk factors) prevention actions, although presenting much better cost effective results, demand more time for their appearance. For this reason, huge amounts are spent on secondary or even primary prevention measures, but with a focus on medicalization which, in an misleading way, shows favorable changes in short-term statistics and may even give political results with immediatist benefits.^10,620-626^

It is evident that population intervention must involve the involvement of society as a whole. It should be part of a government policy, and linked in a partnership with organized civil society, non-governmental organizations, and industries in general, especially food producers and beneficiaries. Every action will only achieve the expected objectives if it is developed collectively, with multidimensional action.^10,620-626^

It must be highlighted, particularly for hypertension, but has a strong interface with other CV risk factors:

- Education as a whole and, in particular, health education for the dissemination of knowledge about CV risk factors and the understanding of the importance of healthy lifestyles;^10,620-626^
- Legislation enforcement which encourages the production of healthy foods, and discourages and foods harmful to health;^10,624,625^
- The encouragement of families to have healthy lifestyles, with the possibility of monetary benefits in case of lifestyle changes (maintenance of ideal weight, decreased sodium intake, regular physical activity, increased fruit intake, vegetables and cereals, smoking cessation);
- The provision of safe areas for the practice of regular physical activity;
- Provision of a simplified means for basic assessment of key CV risk factors (BP, body mass index, blood glucose, cholesterol and smoking status);^2,620^
- Access to basic medicines when preventive measures fail and the use of drugs to prevent disease is needed.^10,620^

### 12.6. Population Aspects of Dyslipidemia

Scientific knowledge leaves no doubt about the relevance of dyslipidemias as an important risk CV factor.^2,628^ There is also a general recognition that individual or even collective actions aimed at treatment, although useful and beneficial, are very expensive and much lower cost effective, even in developed countries.^2,627-631^

From these premises we have opened a huge door of opportunity. It is unthinkable to admit that the health system, especially in developing countries, such as Brazil, can adequately afford the high costs of treating established diseases.^2,629,630,632^

Thus, population-based primary prevention becomes a cost-effective and absolutely sustainable long-term alternative.^633-636^

This must be the fundamental mission of any government.

Public policies for food quality control, health education at all levels, with priority for young people and, finally, a health system that allows universal access to care and when necessary, drugs as the last option.^633,637,638^

It should be noted that small reductions in each of the risk factors may promote large reductions in CV events. Additional benefit can be obtained through the adoption of healthy lifestyles in society that will bring benefits to all risk factors that are completely interconnected (smoking, poor diet, overweight, dyslipidemia, AH and physical inactivity).^632,634,636^

Thus the entire population, with an initial focus on children and adolescents, should be encouraged to adopt healthy diets, maintain adequate weight or decrease weight for this purpose, practice regular physical activity with at least moderate intensity and smoking cessation.

The government should offer political, legal and financial conditions for the implementation of these actions in the educational field for the entire population.^2,627,628,636^

#### 12.6.1. General Practice Measures^639^

- Encourage exclusive breastfeeding up to at least 6 months;
- Decrease salt content in the preparation of processed and industrialized foods;
- Encourage the consumption of fruit and vegetables, as well great supply and accessibility;
- Decrease intake of saturated and trans fats, replacing with unsaturated fats;
- Decrease sugar content in industrialized beverages;
- Reduce food portion sizes and limit excessive caloric intake;
- Healthy food supply in all public institutions;
- Incentive and collaboration policies with producers for the production and commercialization of healthy foods;
- Incentives and continuous health education policy for the population as a whole (with emphasis on children and adolescents;
- Improved labeling of processed and industrialized foods.

### 12.7. Population Aspects of Physical Activity

Physical activity includes all forms of human movement and active life, including walking, exercise, as well as sports, and is a natural behavior that confers many benefits.^648,649^

The urgency of addressing NCD, including CVD, which contribute to a significant burden of premature death, disease, disability, and economic burden for all countries is emphasized.^648,649^ To reaffirm that physical inactivity is a major factor in modifiable risks to NCD. As an important point of the strategy to reduce the burden of NCD, as articulated in the WHO Global Action Plan for NCD prevention and control, 2013-2020.^627,648,649^

Recognizing this strong link between physical activity and major noncommunicable diseases, WHO member states agreed on a relative 10% reduction in the prevalence of physical inactivity by 2025 as one of nine global targets for improving the prevention and treatment of noncommunicable diseases.

In Brazil, according to VIGITEL 2017, physical activities practiced in four domains (leisure, occupational activity, commuting and domestic activities), which allow building multiple indicators of physical activity standard.^650^

In addition, the frequency of adults who, in their free time spend: a) three or more hours of the day watching television; b) three or more hours of the day using computer, mobile or tablet; and c) three or more hours of the day watching television or using a computer, mobile phone or tablet.

The frequency of adults practicing leisure time physical activity equivalent to at least 150 minutes of moderate physical activity per week ranged from 29.9% in São Paulo to 49.6% in the Federal District. Among men, the highest frequencies were found in Macapá (57.1%), São Luís (54.1%) and Distrito Federal (53.8%) and the lowest in São Paulo (36.0%), João Pessoa. (39.5%) and Fortaleza (42.1%). Among women, the highest frequencies were observed in the Federal District (45.9%), Palmas (41.9%) and Curitiba (37.7%). The smallest were in São Paulo (24.8%), Porto Alegre (26.7%) and Recife (28.1%), 650 showing a high prevalence of sedentary individuals.

Physical activity in leisure time is increasing. In 2009, the indicator was 30.3%, and in 2016, 37.6%. Prevalence decreases with age, being more common among young people from 18 to 24 years old.^650^

The situation is not different from other countries, whether developed or developing. Prevalence in 2016 was more than twice as high in high-income countries (36.8%, 35.0-38.3%) than in low-income countries (16.2%, 14.9-9.9) and physical inactivity increased in high-income countries over time (31.6%, 27.1-37.2 in 2001). If current trends continue, the global physical activity target for 2025 (a relative 10% reduction in physical inactivity) will not be met. Policies to increase population levels of physical activity need to be prioritized and expanded urgently.^649^

Physical inactivity is one of the top 10 risk factors for global mortality, causing about 3.2 million deaths each year.^651,652^ Sedentary adults have a 20-30% increase in risk of all mortality causes compared with those who do at least 150 minutes of moderate physical activity per week, or equivalent, as recommended by WHO. Regular physical activity reduces the risk of ischemic heart disease, stroke, diabetes, and breast and colon cancer. In addition, regular physical activity is a major determinant of energy expenditure and is therefore critical to energy balance, weight control and obesity prevention.^651,652^

### 12.8. Population Approach to Increased Physical Activity

The proposed policy options aim to promote the implementation of the global strategy regarding diet, physical activity and health and other relevant strategies, and to promote the additional benefits of increasing physical activity levels in population, such as improved educational performance and social and mental health benefits , coupled with cleaner air, reduced traffic, less congestion and links to healthy child development and sustainable development.^654,655^

In addition, interventions to increase participation in physical activity throughout the population for which favorable cost-effectiveness data are emerging and should be promoted. The objective is to contribute to achieving the voluntary global goals listed below:^654,655^

A relative 10% reduction in the prevalence of physical inactivity.

- It can stop the rise of diabetes and obesity.
- Lead to a 25% relative reduction in the prevalence of hypertension or contain the prevalence of increased BP according to national realities.

Proposed policy options include:

- Adopt and implement national guidelines on physical activity for health.
- Consider establishing a multisectoral committee or similar body to provide strategic leadership and coordination.
- Develop appropriate partnerships and involve all segments of society concerned, levels of government, non-governmental organizations (NGO), civil society, scientific societies and economic operators, in the active and appropriate implementation of actions aimed at increasing physical activity at all ages.
- Develop policy measures in cooperation with relevant sectors to promote physical activity through activities of daily living, including through “active transport”, recreation, leisure and sport, for example: National, state and municipal urban planning and transport policies to improve accessibility, acceptability and safety of support infrastructure for walking and cycling.
- Improvement in the provision of quality physical education in educational settings (for elementary and high school students) including opportunities for physical activity before, during and after the formal school day.

Actions to support and encourage “physical activity for all” initiatives.

- Creation and preservation of natural environments that facilitate physical activity in schools, universities, workplaces, clinics and hospitals, and in the wider community, with a particular focus on providing infrastructure to support active transportation such as walking and cycling, recreation and active play and participation in all types of sports.
- Promote community involvement in implementing local actions to increase physical activity.
- Conduct evidence-based public campaigns through mass media, social media, and community and social marketing initiatives to inform and motivate adults and youth about the benefits of physical activity and facilitate healthy behaviors. Campaigns should be linked to supportive actions across the community for maximum benefit and impact.

Encourage the evaluation of actions aimed at increasing physical activity to contribute to the development of an evidence base for effective and cost-effective actions.^654,655^

### 12.9. Socioeconomic and Environmental Factors and Associated Diseases in Cardiovascular Prevention

The main determinants of population health are multiple and classifiable in the fields of biology, environment (physical, social and economic), behaviors (lifestyle) and health care.^656^ It is estimated that major, socioeconomic determinants , represent 75%, while genetic, biological and behavioral factors together account for approximately 25% of the population’s health^657,658^ (Chart 12.1).

**Chart 12.1:** Examples of health determinants divided by socioeconomic and environmental categories^596^

Environmental determinates	Water and air pollution, biodiversity, global warming, ozone depletion, housing conditions, transport quality, food safety, waste management, energy policy, urban environment
Economic determinates	Country economic performance, per capita income, access to health services, employment conditions, housing, security, transportation
Social determinates	Culture, lifestyles, gender, ethnicity, degree of social inclusion, age, health-related behaviors, living conditions, working conditions, education

**Figure 12.4:**
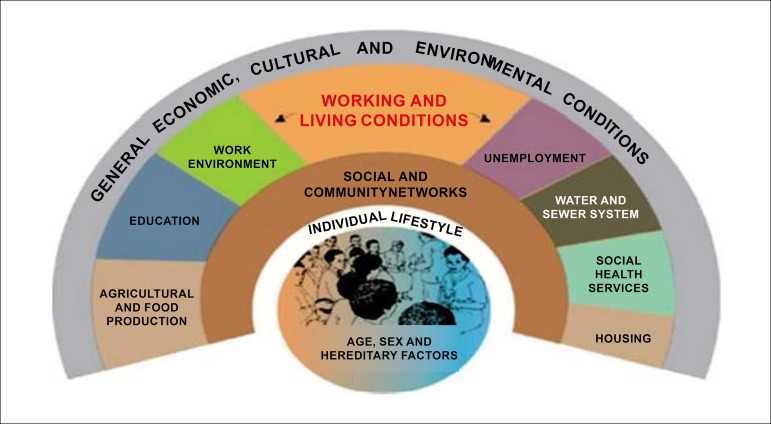
Socioeconomic and environmental determinants: Dahlgren and Whitehead model. Source: Carvalho A.^659^

The literature reports different models that intend to describe the complex relationship between the multiple factors that influence the socioeconomic determinants of health, one of the most mentioned is the Dahlgren and Whitehead^659^ model (Figure 12.4).

According to Rose,^660^ the socioeconomic determinants underlie the pyramid of health inequalities and, consequently, the right to health cannot be guaranteed only by the health sector, requiring economic and social public policies.^660^ Prospective studies have shown that in Brazil and in developed countries, low socioeconomic status defined as low status employment, low educational and income levels, and living in poorer residential areas contribute to increased CV mortality and all-cause death.^664-668^

Through a macroeconomic indicator, represented by the Gross Domestic Product per capita (GDPpc) from 1979 to 2010 of municipalities of the State of Rio de Janeiro, the relationship between this indicator and the reduction in mortality from circulatory system diseases was analyzed. The decrease in mortality was preceded by an increase in GDPpc, with a strong association between the indicator and mortality rates, showing the importance of improving the living conditions of the population in the reduction of CV mortality.^664^ The American Heart Association Document , on the influence of social factors on CVD, revealed that populations with lower educational levels have a higher prevalence of CV risk factors, a higher incidence of CV events, and a higher CV mortality rate, regardless of other demographic factors.^665^

### 12.10. Health and Sustainable Development

Health is a timeless value. Good health is a precondition for work and a measure of sustainable development.^666^ The WHO created the “Commission of Social Determinants of Health” in 2005 to define health promotion directed at “health equity” in populations, and a global movement to reach it. In an extensive evidence-based publication, WHO prioritized the following actions: early childhood education, healthy housing, urban and rural infrastructure, universal access to health and other services, employment, quality social protection, inclusion social, gender equality. Regardless of policy options, it advocated health equity in all Policies, Systems and Programs through fair financing and “Good Global Governance”.

It recommended, as an example, the United Nations (UN) Millennium Project, prepared in 2000 during the Millennium Summit, the largest meeting of world leaders, aimed at establishing a global partnership to reduce extreme poverty.^667,668^ The 2015 goals, known as the “Millennium Development Goals (MDG)”, represented a paradigm shift to improve the health of vulnerable and disadvantaged groups. They are: eradicate extreme poverty and hunger; implement a universal basic education; promote gender equality and empower women; reduce child mortality; improve maternal health; combat AIDS, malaria and other infectious diseases; ensure environmental sustainability and develop a global partnership for development.

Subsequently, in 2015 the WHO Health report “Health in 2015: from MGDs to SDGs” highlighted health progress on the MDG and redefined priority actions to achieve the new “Goals for Sustainable Development” (SDG).^668^ The SDG, which make up Agenda-2030, contain more numerous and ambitious actions (17 goals, 169 goals) than the MDG (8 goals, 21 goals).^668^ It recognizes that improving the health of people depends on social justice, environmental protection (climate change, heat waves, droughts, fires, storms, floods), polluting energies, antibiotic resistant agents, aging, migrations, increased global burden of NCD, indivisible pillars of sustainable development.^669^ (Chart 12.2).

**Chart 12.2:** Sustainable Development Goals, WHO 2015^669^

Sustainable development goals
1. Eradicate poverty
2. End hunger
3. Promote health and well-being
4. Quality and inclusive education
5. Gender equality
6. Clean water and sanitation
7. Clean, renewable energy
8. Employment, decent work and economic growth
9. Innovation in Resilient Infrastructure
10. Reduce inequalities within and between countries
11. Sustainable cities and communities
12. Sustainable production and consumption
13. Tackle climate change
14. Use the seas and marine resources sustainably
15. Promote sustainable use of terrestrial ecosystems;
16. Peace, justice and sound institutions
17. Implement global partnership

In this context, Brazil launched the “Strategic Action Plan for Coping with NCD in Brazil, 2011-2022” at the UN assembly and implemented a CNCD Surveillance System (VIGITEL) over the last decade that allows the national and global monitoring of NCD targets, representing a breakthrough in NCD Surveillance in the country.^670^ Between 2000 and 2011, Brazil recorded an average decline of 2.5% per annum in all major NCD, with a significant decrease of 3.3% in CVD, observed in both sexes and in all regions of the country.^671^ However, between 2015 and 2016 there was a trend of stability in mortality rates due to NCD, which may be a consequence of the change in risk factor behavior and worsening of risk factors, living conditions (access to services, unemployment) caused by the economic and the social crisis.^672,673^ If these trends are maintained, Brazil may not meet the WHO-UN target set for the reduction in premature mortality from NCD in Agenda - 2030.

### 12.11. Cardiovascular Prevention, Environment, Sustainability and Associated Diseases

Hippocrates, the author of “Airs, Waters, and Places” (400 BC) was probably the first to recognize a relationship between disease and the environment, including the effects of climate and lifestyle.^674^ Numerous aspects regardinf the quality of the physical environment (air pollution, cycle paths, green areas, parks) and behavioral factors (smoking, high-fat diets, physical inactivity) are determining factors for increasing or decreasing risks for CVD.^675^ Since 2004, the American Heart Association has recognized exposure to air pollution as an important modifiable risk factor for CVD morbidity and mortality in populations, with a higher risk attributable to particulate matter (PM) over gaseous components.^676^ Particulate matter < 2.5 µm (PM2.5) is the most important environmental risk factor, with higher risk than gaseous components, posing a major threat to public health.^677^ Short-term PM2.5 elevations increase the relative risk of acute CV events by 1% to 3% within a few days. Long-term exposures (years) increase the risk by ± 10%, which is partly attributable to the development of cardiometabolic disorders such as high blood pressure, diabetes mellitus, among others.^677^

The pathophysiological mechanisms of changes caused by PM include: increased blood viscosity, vascular reactivity, induction of a systemic inflammatory state (thrombosis), changes in cardiac autonomic control (arrhythmia, hypertension), development and progression of atherosclerosis (acute myocardial infarction) heart failure and other CVD.^676^

The finer particles are more harmful to CV health given their greater ability to penetrate the airways. When inhaled, they penetrate deep into the lung tissue, inducing oxidative stress and inflammation through the release of IL-6, IL-1b, TNF-a by macrophages. Parallel to the intense oxidative stress that begins in the lung tissue, trophic effects on vascular and cardiac cells occur, increased generation of reactive oxygen species, impairment of nitric oxide-mediated vasodilation, endothelial dysfunction and, consequently, development and/or progression of atherosclerosis.^678^

The Harvard Six Cities study, involving populations from six US cities, revealed that the risk of myocardial infarction in a city with polluted air increases by 5% compared with that of clean air.^679^ In the city of São Paulo it was observed that pollution is so high that it would be the equivalent to smoking two cigarettes a day.^677^ In the Brazilian Amazon, burning biomass (bush) increased mortality from CV and respiratory events among the elderly, especially from acute myocardial infarction.^680^

Particles and gases released at high altitudes circulate throughout the troposphere and can be transported over long distances, with impacts on a global scale.^680^ After forest fires in Canada, high concentrations (up to 30 times higher) of mostly fine PM have been recorded in the city of Baltimore, in the United States. Thus, environmental threats are not limited only to industrial gases and lead particles from motor vehicles in urban areas, but also to the PM generated by biomass burning in rural areas, and it is estimated that air pollution, a growing public health problem, will double CVD mortality by 2050.^680^

The Expert Position Paper on Air Pollution and Cardiovascular Disease of the European Society of Cardiology revealed that in 2010, air pollution accounted for 3.1 million of the 52.8 million deaths, for all causes and ages. The American Heart Association reports that 60,000 Americans and 6,000 Canadians die each year from short- or long-term exposure to airborne pollutants.681 Study shows that living near (50 m) high-traffic roads can increase risk sudden death.^682^

Other factors related to lifestyle, eating habits, and socioeconomic variables may exacerbate the effects of exposure to pollution, such as smoking, a diet high in fats and sugars, physical inactivity, and the use of licit (alcohol) and illicit (marijuana) drugs.

The key recommendations of the Environment & the Heart Campaign campaign, organized in 2015 by the European Society of Cardiology and the European Heart Network (EHN), to European policy makers to promote a healthy environment for a healthy heart were: 1) Include the air and noise pollution in the group of modifiable risk factors for CVD; 2) Include clean air and noise reduction in all areas of health policy; 3) Adopt WHO air quality limits; 4) Intensely reduce the emission of automotive gases; 5) Promote green urban planning to reduce pollution and promote physical activity; 6) Promote clean forms of energy (low emission vehicles and non-combustion renewable energy sources); 7) Guarantee funds for studies on the effects of environmental stress on the CV system; 8) Support events addressing NCD, social, economic and environmental inequalities regarding access to health.^683,684^ Brian Garvey^685^ from the University of Strathclyde, Scotland, in the preface to Larissa Bombardi’s study “Geography of Pesticides in Brazil and Connections with the European Union”, stated that “every sick community, every poisoned field, every polluted watercourse threatens to extinguish an alternative variety of life”.

**Figure 12.5:**
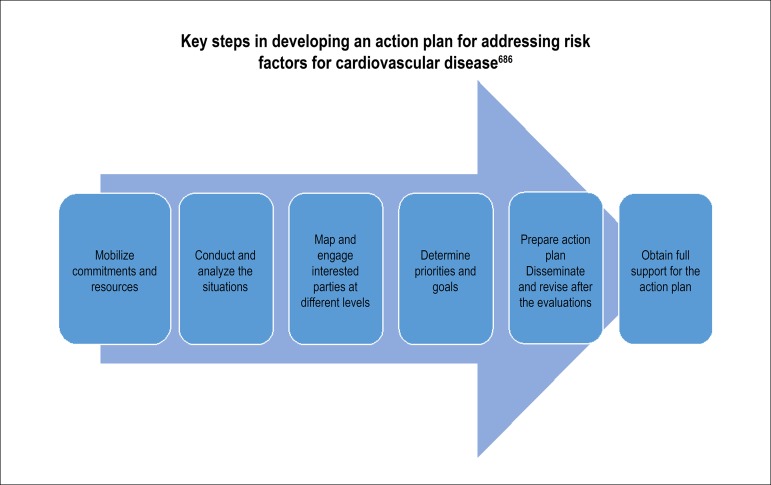
Key steps in developing an action plan for addressing risk factors for cardiovascular disease.685 Adapted from Global status report on NCDs 2014.

The major steps in developing an action plan for addressing CV disease risk factors,^686^ are described in Figure 12.5.

### 12.12. Conclusion

Commitment from universities, scientific societies, organized civil society, state and municipal health secretariats, state and municipal education secretariats, the ministry of health, and federal, state and municipal governments is necessary to implement population-based approaches to address CV disease risk factors. These actions should be state policies aimed at impacting the various related morbidity and mortality indicators, as well as improving the population’s quality of life.

## Figures and Tables

**Table 6.2:** Recommendations for the consumption of and/or supplementation with products rich in omega-3 fatty acids

Recommendation	Recommendation grade	Level of evidence	References
Supplementation with 2-4 grams of marine omega-3 per day or even higher doses should be recommended for severe hypertriglyceridemia (>500 mg/dL in the absence of familial chylomicronemia), with risk for pancreatitis, refractory to non-pharmacological measures and drug treatment	I	A	^235^
At least two fish meals per week should be recommended as part of a healthy diet to decrease the CV risk. This recommendation is particularly aimed at individuals at high risk, such as those who already had myocardial infarction	I	B	^32^
Omega-3 supplementation (EPA) at a dose of 4 g per day can be administered to patients in secondary prevention who use statins and have TG between 150-499 mg/dL	II	B	^227^
Omega-3 supplementation at a dose of 1 g/day (EPA+DHA) can be administered to patients with HF functional class II to IV	II	B	^235^
Supplementation with EPA+DHA is not recommended for individuals in primary prevention, whether or not they are on preventive treatments based on evidence	III	A	^231^

CV: cardiovascular; DHA: docosahexaenoic acid; EPA: eicosapentaenoic acid; HF: heart failure; TG: triglycerides.

**Table 8.3:** Recommended levels of physical exercise to reduce cardiovascular risk

Recommendation	Recommendation class	Level of evidence	Reference
During consultations, doctors should advise their patients about PA	I	B	^341^
Weekly PA of ≥ 150 minutes of moderate intensity exercise or 75 minutes of more intense exercise reduces CV risk	I	A	^341^
Weekly PA of < 150 minutes of moderate intensity exercise or <75 minutes of more intense exercise reduces CV risk	IIa	B	^341^

CV: cardiovascular; PA: physical activity.
